# A chromosomal-level genome assembly for the giant African snail *Achatina fulica*

**DOI:** 10.1093/gigascience/giz124

**Published:** 2019-10-21

**Authors:** Yunhai Guo, Yi Zhang, Qin Liu, Yun Huang, Guangyao Mao, Zhiyuan Yue, Eniola M Abe, Jian Li, Zhongdao Wu, Shizhu Li, Xiaonong Zhou, Wei Hu, Ning Xiao

**Affiliations:** 1 National Institute of Parasitic Diseases, Chinese Center for Disease Control and Prevention; Key Laboratory of Parasite and Vector Biology, Ministry of Health; WHO Collaborating Centre for Tropical Diseases; Chinese Centre for Tropical Diseases Research, Shanghai 200025, P. R. China; 2 State Key Laboratory of Genetic Engineering, Ministry of Education Key Laboratory for Biodiversity Science and Ecological Engineering, Ministry of Education Key Laboratory of Contemporary Anthropology, School of Life Science, Fudan University, Shanghai 200438, China; 3 Department of Parasitology, Zhongshan School of Medicine, Sun Yat-sen University, Guangzhou 510080, China

**Keywords:** giant African snail, *Achatina fulica*, Pacific Biosciences, Hi-C, chromosome assembly

## Abstract

**Background:**

*Achatina fulica*, the giant African snail, is the largest terrestrial mollusk species. Owing to its voracious appetite, wide environmental adaptability, high growth rate, and reproductive capacity, it has become an invasive species across the world, mainly in Southeast Asia, Japan, the western Pacific islands, and China. This pest can damage agricultural crops and is an intermediate host of many parasites that can threaten human health. However, genomic information of *A. fulica* remains limited, hindering genetic and genomic studies for invasion control and management of the species.

**Findings:**

Using a *k-*mer–based method, we estimated the *A. fulica* genome size to be 2.12 Gb, with a high repeat content up to 71%. Roughly 101.6 Gb genomic long-read data of *A. fulica* were generated from the Pacific Biosciences sequencing platform and assembled to produce a first *A. fulica* genome of 1.85 Gb with a contig N50 length of 726 kb. Using contact information from the Hi-C sequencing data, we successfully anchored 99.32% contig sequences into 31 chromosomes, leading to the final contig and scaffold N50 length of 721 kb and 59.6 Mb, respectively. The continuity, completeness, and accuracy were evaluated by genome comparison with other mollusk genomes, BUSCO assessment, and genomic read mapping. A total of 23,726 protein-coding genes were predicted from the assembled genome, among which 96.34% of the genes were functionally annotated. The phylogenetic analysis using whole-genome protein-coding genes revealed that *A. fulica* separated from a common ancestor with *Biomphalaria glabrata* ∼182 million years ago.

**Conclusion:**

To our knowledge, the *A. fulica* genome is the first terrestrial mollusk genome published to date. The chromosome sequence of *A. fulica* will provide the research community with a valuable resource for population genetics and environmental adaptation studies for the species, as well as investigations of the chromosome-level of evolution within mollusks.

## Data Description

### Introduction

**Figure 1:**
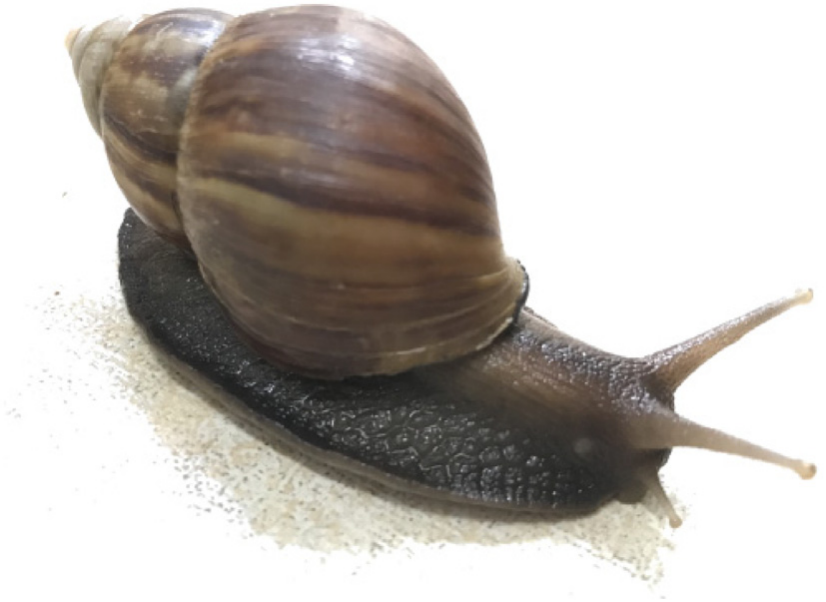
*A. fulica* individual used for genome sequencing and assembly.

The giant African snail, *Achatina fulica* (NCBI:txid6530), is a Gastropod species (Fig. 1). It is the largest terrestrial mollusk, with a voracious appetite, strong environmental adaptability, and high growth and reproduction rate [1–3]. Originating in East Africa, *A. fulica* over the past century has gradually invaded Southeast Asia, Japan, and the western Pacific islands [4–6] with the direct and indirect help of humans [7–9]. In mainland China, the first *A. fulica* invasion event was reported in 1931 [10]. At present, the snail has been found in the wild in Guangdong, Hainan, Guangxi, southern parts of Yunnan Province and Fujian Province, and a county of Guizhou Province [11]. *A. fulica* was included among the first 16 alien invasive species designated in China [12] in 2003 and was also listed by the International Union for Conservation of Nature as among the 100 most threatening alien invasive species [13]. This snail has been recognized as an agricultural and garden pest causing significant damage in both tropical and subtropical regions [9, 13, 14]. In addition, *A. fulica* is also the intermediate host of the parasitic nematode *Angiostrongyl cantonensis*. Human infection with angiostrongyliasis, which occurs mainly through consumption of snails carrying *A. cantonensis* larvae, causes eosinophilic meningoencephalitis [4, 11, 15–19]. As a consequence, *A. fulica* is attracting more and more attention in the fields of agricultural crop protection and human disease control.

To date, a variety of mollusk genomes have been analyzed and published, including those of 2 freshwater gastropod snails, *Pomacea canaliculata* [20] and *Biomphalaria glabrata* [21]. However, no genome has been reported for terrestrial mollusks. *A. fulica* is considered to be a destructive terrestrial gastropod that poses a significant hazard to agriculture, the environment, biodiversity, and human health. A chromosome-level genome of *A. fulica* could provide crucial resources in population genetics and evolution studies based on genomic sequencing data aiming to elucidate its invasion and adaptation history. Furthermore, the genome could also be used to probe gene expression during important biological processes, such as gene expression patterns in various developmental stages and the interaction of *Angiostrongylus* and *A. fulica*. In the present study we applied Illumina, PacBio, and Hi-C techniques to construct the chromosome of *A. fulica*. The genome is the first terrestrial mollusk genome, providing an important reference for the molecular mechanisms underlying its broad environmental adaptability and the development of a control strategy for its worldwide invasion.

### Sample and sequencing

An adult snail (Fig. 1), which was collected in Pingxiang city, Guangxi Autonomous Region, was used for reference genome construction. The snail was dissected and abdominal foot (17.4 g) and liver pancreas (40.4 g) tissues were collected and quickly frozen in liquid nitrogen overnight before transfer to storage at −80°C. DNA was extracted using the traditional phenol/chloroform extraction method and was quality checked using agarose gel electrophoresis, meeting the requirement for library construction for the Illumina X Ten (Illumina Inc., San Diego, CA, USA) and for the PacBio Sequel (Pacific Biosciences, Menlo Park, CA, USA) sequencing platforms.

RNA was extracted from the pallium, liver, foot, spleen, stomach, gut, and heart using TRIzol® Reagent (Life Technologies, Gaithersburg, USA). The RNA quality was checked using the Nanodrop ND-1000 spectrophotometer (LabTech, USA) and 2100 Bioanalyzer (Agilent Technologies, USA) with RNA integrity number >8 (Supplemental Table S1 and Supplemental Fig. S1). The RNA from each sample was equally mixed for the RNA sequencing on the PacBio Sequel platform. First, messenger RNA molecules were reverse-transcribed to complementary DNA (cDNA) using Clontech SMARTer cDNA synthesis kit. After cDNA amplification and purification, 2 SMRTbell libraries of 0–4 and 4–10 kb were generated using the size selection in the BluePippin Size Selection System (Pacific Biosciences) and protocols suggested by the manufacturer. The final libraries were sequenced in the PacBio SEQUEL platform (Pacific Biosciences), resulting in 12,439,996 subreads totaling ∼22.5 Gb PacBio long reads with average length >1,801 bps. Subsequently, a total of 782,613 circular consensus sequences were generated on the basis of the subreads, and 553,889 full-length non-chimeric sequences (FLNC) representing 23,726 gene loci were ultimately obtained. All aforementioned data processing was performed using SMRT Link v5.0 [22]. Moreover, ∼70.37% of the multi-exon FLNCs were really full-length sequences embracing all the exons of the gene locus predicted from the whole-genome sequences.

Using the DNA from the abdominal foot, a library with insertion length of 300 bp was constructed and sequenced using the Illumina sequencing platform according to the manufacturer's protocol. Approximately 202.23 Gb short reads were obtained using Illumina X Ten sequencing technology (Table 1), which was used for the following genome survey analysis, and for final base-level genome sequence correction. Meanwhile, four 20-kb libraries were constructed for PacBio Sequel sequencing. Using 16 sequencing SMRT cells, 104.6-Gb long reads were generated (Table 1). The mean and N50 lengths of the polymerases for sequencing cells ranged from 6.4 to 10.4 kb and from 12.3 to 20.3 kb, respectively. Those long genomic DNA reads were then used for reference genome construction.

**Table 1:** Sequencing data generated for *A. fulica* genome assembly and annotation

Library type	Platform	Library size (bp)	Data size (Gb)	Application
Short reads	HiSeq X Ten	350	202.24	Genome survey and genomic base correction
Long reads	PacBio SEQUEL	20,000	101.63	Genome assembly
Hi-C	HiSeq X Ten	300–500	199.73	Chromosome construction

### Genome feature estimation from *k-*mer method

With sequencing data from the Illumina platform, several genome characteristics could be evaluated for *A. fulica*. To ensure the quality of the analysis, ambiguous bases and low-quality reads were trimmed and filtered using the HTQC package (version 1.92.3) [23]. The following quality controls were performed under the framework of HTQC. First, the quality of bases at 2 read ends were checked. Bases in sliding 5-bp windows were deleted if the average quality of the window was below phred quality score of 20. Second, reads were filtered if the average phred quality score was <20 or the read length was <75 bp. Third, the mate reads were also removed if the corresponding reads were filtered.

The quality-controlled reads were used for genome character estimation. We calculated the number of each 17-mer from the sequencing data using the Jellyfish software (Jellyfish, RRID:SCR_005491; version 2.0) [24], and the distribution was analyzed with GCE software (GCE, RRID:SCR_017332; version 3) [25] and is shown in Supplemental Fig. S2. We estimated the genome size at 2.12 Gb with heterozygosity of 0.47% and repeat content of 71% in the genome. Previous studies have revealed that repeat content varies in mollusks and is correlated with genome size [26]. The large genome size and high proportion of repeat content of *A. fulica* provided additional supporting data for the statistical analysis. Moreover, 10,000 pairs of short reads were extracted randomly and were compared to the nt database and no obvious external contamination was found.

### Genome assembly by third-generation long reads

After removing adaptor sequences in polymerases, 101.6-Gb subreads were generated for the following whole-genome assembly. The average and N50 length of subreads reached 5.25 and 8.80 kb, respectively. To optimize the genome assembly using the PacBio sequencing data, we applied 2 packages in the assembly process, Canu v1.8 (Canu, RRID:SCR_015880) [27] and FALCON v0.2.2 (Falcon, RRID:SCR_016089) [28]. The Canu package was first applied for the assembly using default parameters. As a result, a 1.93-Gb genome was constructed with 10,417 contigs and a contig N50 length of 662.40 kb. FALCON was also employed using the length_cutoff and pr_length_cutoff parameters of 10 and 8 kb, respectively. We obtained 1.85 Gb genome with 8,585 contigs, with a contig N50 of 726.63 kb. We adopted the FALCON assembly as the reference genome for *A. fulica* (Table 2). Compared with the estimated genome size, the assembled version was relatively smaller, which may have resulted from the following 2 possible scenarios: the high repeat content of the genome and the probably larger size estimated from the *k*-mer analysis. The genome sequences were subsequently polished, PacBio long reads using Arrow [29] and Illumina short reads using Pilon [30] to correct base errors. The corrected genome was further applied for the following chromosome assembly construction using Hi-C data.

**Table 2:** Statistics for genome assembly of *A. fulica*

Sample ID	Length (bp)		Number	
	Contig**	Scaffold	Contig**	Scaffold
Total	1,852,282,574	1,855,883,074	8,211	1,010
Max	5,947,392	116,558,012		
N50	721,038	59,589,303	697	13
N60	538,883	58,013,356	995	16
N70	399,612	53,672,006	1,396	20
N80	268,901	50,673,968	1,957	23
N90	141,756	44,109,545	2,888	27

**This indicates the ultimate contigs because they were probably modified during the Hi-C step.

### *In situ* Hi-C library construction and chromosome assembly using Hi-C data

Liver pancreas tissue of *A. fulica* was used for library construction for Hi-C analysis, and the library was constructed using the identical method as previous studies [31]. Finally, the library was sequenced with 150 paired-end mode on the Illumina HiSeq X Ten platform (San Diego, CA, United States). From the Illumina sequencing platform, 1,313.87 million paired-end reads were obtained for the Hi-C library (Table 1). The reads were mapped to the above *A. fulica* genome with Bowtie2 [32], with 2 ends of paired reads being mapped to the genome separately. To increase the interactive Hi-C read ratio, an iterative mapping strategy was performed as in previous studies, and only read pairs with both ends uniquely mapped were used for the following analysis. From the alignment status of the 2 ends, self-ligation, non-ligation, and other sorts of invalid reads, including StartNearRsite, PCR amplification, random break, LargeSmallFragments, and ExtremeFragments, were filtered out by Hi-Clib [33]. Through the recognition of restriction sites in sequences, contact counts among contigs were calculated and normalized.

**Figure 2:**
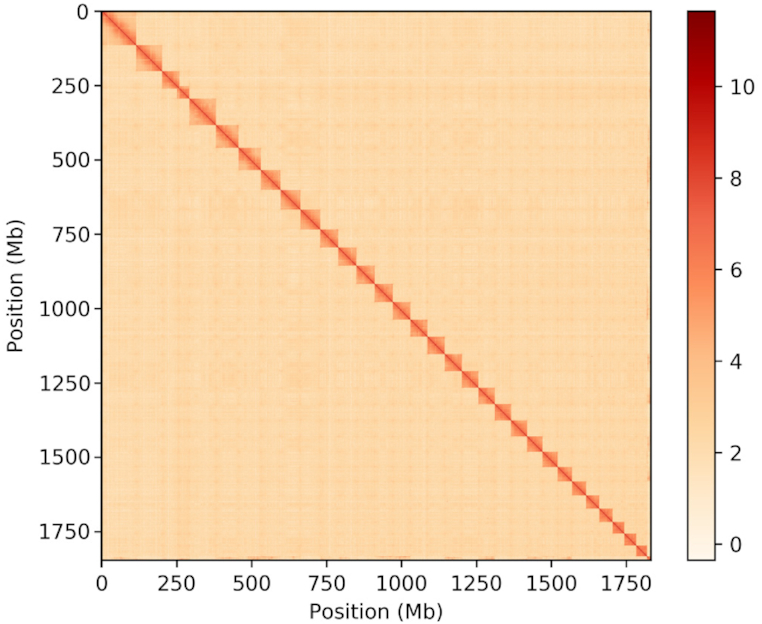
Contact matrix generated from the Hi-C data analysis showing sequence interactions in chromosomes. Color bar indicates the logarithm of the contact density.

According to previous karyotype analyses, *A. fulica* has 31 chromosomes [34]. By clustering the contigs using the contig contact frequency matrix, we were able to correct some minor errors in the FALCON assembly results. Contigs with errors were broken into shorter contigs. We obtained 8,701 contigs, slightly more than the 8,585 contigs in the FALCON assembly. We successfully clustered these contigs into 31 groups in Lachesis [35] using the agglomerative hierarchical clustering method (Fig. 2). Lachesis was further applied to order and orient the clustered contigs according to the contact matrix. As a result, 7,106 contigs were reliably anchored, ordered, and oriented on chromosomes, accounting for 99.32% of the total genome bases. The first near chromosomal-level assembly of *A. fulica* was obtained with 8,211 contigs, a contig N50 of 721.0 kb, and a scaffold N50 of 59.59 Mb (Tables 2 and 3).

**Table 3:** Summary of the genome of *A. fulica* and other published mollusk genomes

Species	Size* (Mb)	Contig N50 (kb)	Scaffold N50 (kb)
*Achatina fulica* (this study)**	2,120	721	59,590
*Pomacea canaliculata* [20]**	570	995	38,000
*Crassostrea gigas* [36]	545	7.5	401
*Pinctada fucata* [37]	1,150	1.6	14.5
*Pinctada fucata new* [38]	1,150	21	324
*Pinctada fucata* V2 [39]	1,150	21	167
*Biomphalaria glabrata* [21]	931	7.3	48
*Ruditapes philippinarum* [40]	1,370	3.3	32.7
*Patinopecten yessoensis* [41]**	1,430	38	41,000
*Radix auricularia* [42]	1,600	0.324	578
*Octopus bimaculoides* [43]	2,800	5.4	470
*Mytilus galloprovincialis* [26]	1,600	2.6	2.9
*Lottia gigantea* [44]	420	96	1,870
*Patella vulgata* [45]	1,460	3.1	3.1
*Aplysia californica*	1,760	9.6	917
*Conus tribblei* [46]	2,760	0.85	215
*Limnoperna fortunei* [47]	1,600	10	312
*Bathymodiolus platifrons* [48]	1,640	13.2	343
*Modiolus philippinarum* [48]	2,380	19.7	100.2
*Chlamys farreri* [49]	1,200	1.2	1.5
*Lingula anatina* [50]	463	55	294
*Argopecten prupruatus* [51]	885	80.1	1,020

* Estimated size of the genome.

** Genomes assembled into near chromosomal level.

### Genome quality evaluation

We assessed the quality of the genome of *A. fulica* after the assembly process. The quality evaluation was carried out in 3 aspects: continuity, completeness, and the mapping rate of next-generation sequencing data.

First of all, we compared the sequence number and contig N50 length of *A. fulica* with publicly available mollusk genomes and found that our assembly has a high quality on contig and scaffold N50 among mollusk genomes (Table 3). Traditional chromosomal genome assembly requires physical maps and genetic maps, which is enormously time- and labor-consuming. With Hi-C data analysis, we successfully assembled the *A. fulica* genome into near chromosome-level with just 1 individual.

Second, the assembled genome was subjected to BUSCO (version 3.0, metazoa_odb9) [52] to assess the completeness of the genome. Approximately 91.7% of the BUSCO genes were identified in the *A. fulica* genome, and >84.7% of the BUSCO genes were single-copy completed in our genome, illuminating a high level of completeness of the genome.

Third, next-generation sequencing short reads were aligned to the genome using the BWA package (BWA, RRID:SCR_010910; version 0.7.17) [53], and ∼98.7% of paired reads were aligned to the genome, of which 98.24% were reads that were pair aligned.

### Repeat element and gene annotation

Tandem Repeat Finder 4.09 (TRF) [54] was used for repetitive element identification in the *A. fulica* genome. A *de novo* method applying RepeatModeler (RepeatModeler, RRID:SCR_015027) was used to detect transposable elements (TEs). The resulting *de novo* data, combined with the known repeat library from Repbase [55], were used to identify TEs in the *A. fulica* genome by means of RepeatMasker4–0-8 (RepeatMasker, RRID:SCR_012954) [56] software. All repetitive elements were masked in the genome before protein-coding gene prediction.

**Figure 3:**
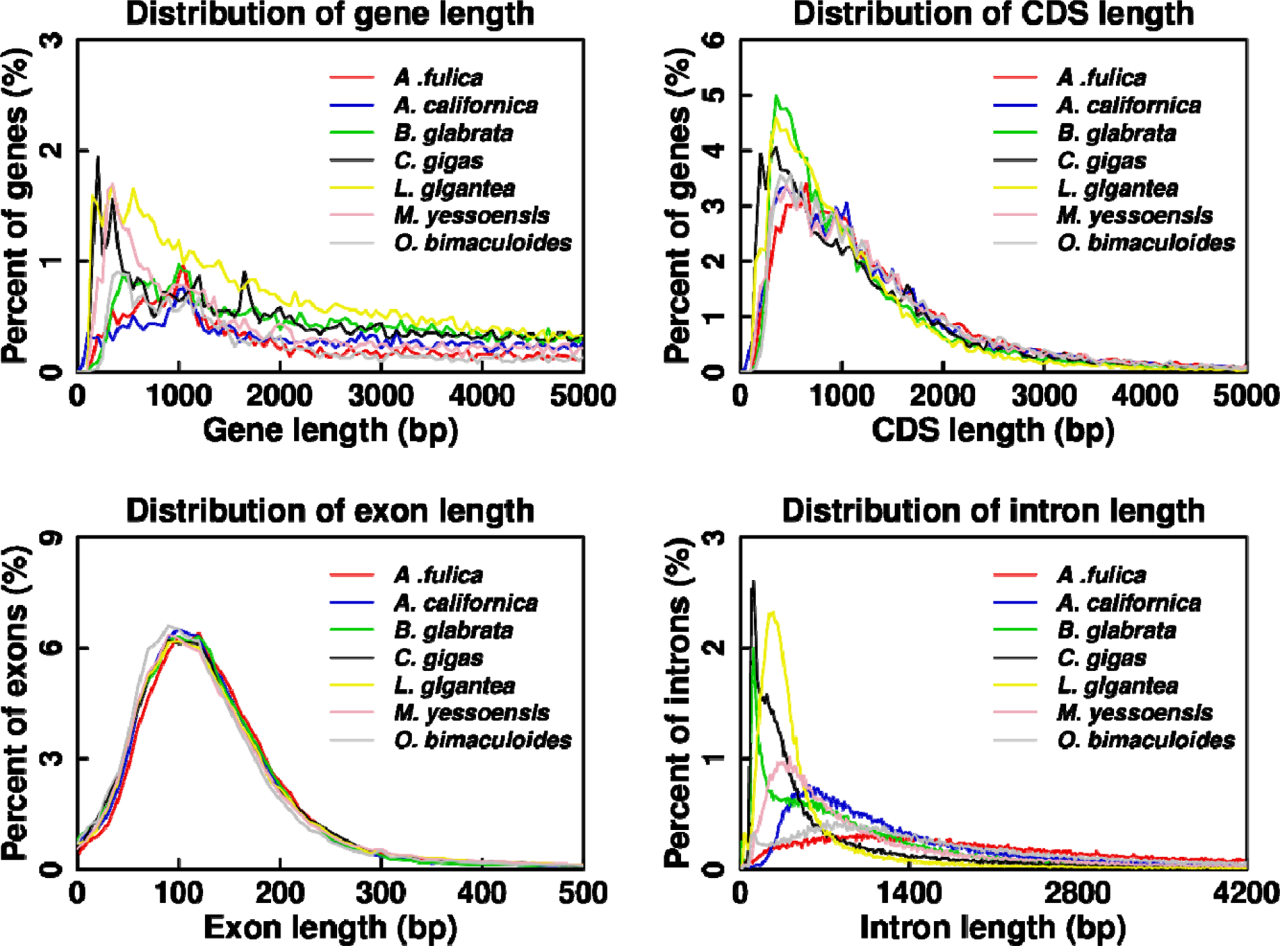
Length distribution comparison of genes (A), CDSs (B), exons (C), and introns (D) for *A. fulica* to those in the closely related mollusk species *A. californica, B. glabrata, C. gigas, L. gigantea, P. yessoensis*, and *O. bimaculoides*.

Protein-coding genes in the *A. fulica* genome were annotated using the *de novo* program Augustus 0.2.1 (Augustus, RRID:SCR_008417) [57]. Protein sequences of closely related species including *Aplysia californica, B. glabrata, Crassostrea gigas, Lottia gigantea*, and *Patinopecten yessoensis* were downloaded from the Ensembl database and aligned to the *A. fulica* genome with TBLASTN2.6.0 (TBLASTN, RRID:SCR_011822). Full-length transcripts obtained using Iso-Seq were mapped to the genome using Genewise (GeneWise, RRID:SCR_015054) [58]. Finally, gene models predicted from all above methods were combined by MAKERv2.31.10 (MAKER, RRID:SCR_005309) [59], resulting in 23,726 protein-coding genes. The gene number, gene length, coding DNA sequence (CDS) length, exon length, and intron length distribution were all comparable to those of the related mollusks (Fig. 3).

To functionally annotate protein-coding genes in the *A. fulica* genome, we searched all predicted gene sequences against the NCBI non-redundant nucleotide (NT) and protein (NR) and Swiss-Prot databases using the BLASTN (BLASTN, RRID:SCR_001598) [60] and BLASTX (BLASTX, RRID:SCR_001653) [61] utilities. Blast2GO (Blast2GO, RRID:SCR_005828) [62] was also used to assign gene ontology (GO) [63] and KEGG [64] pathways. A threshold of e-value of 1e−5 was used for all BLAST applications. Finally, 22,858 (96.34%) genes were functionally annotated (Table 4).

**Table 4:** Statistics for genome annotation of *A. fulica*

Database	Number (%)
InterPro	16,252 (68.50)
GO	12,101 (51.00)
KEGG all	21,325 (89.88)
KEGG Orthology	10,161 (42.83)
Swiss-Prot	17,050 (71.86)
TrEMBL	22,403 (94.42)
NR	22,553 (95.06)
Total	23,726 (100)

### Phylogenetic analysis of *A. fulica* with other mollusks

OrthoMCLv1.2 [65] was used to cluster gene families. First, proteins from *A. fulica* and closely related mollusks, including *A. californica, B. glabrata, C. gigas, Lingula anatina, L. gigantea, P. yessoensis, Octopus bimaculoides, Helobdella robusta, P. canaliculata*, and the outgroup, *Drosophila melanogaster*, were all-to-all blasted by the BLASTP (BLASTP, RRID:SCR_001010) [61] utility with an e-value threshold of 1e−5. Only proteins from the longest transcript were used for genes with alternative isoforms. We identified 25,448 gene families for *A. fulica* and the related species; among them 675 single-copy ortholog families were detected.

**Figure 4:**
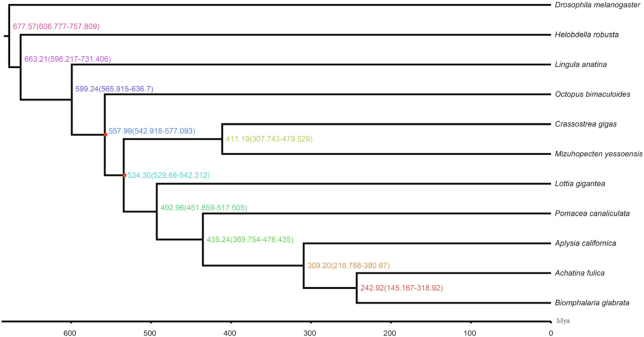
Phylogenetic relationship between *A. fulica* and related species. The divergence times (million years ago [MYA]) with 95% confidence intervals are labeled at branch sites. Red dots in the tree denote the fossil recalibration sites, with a maximum and minimum age of Bivalve/Gastropod divergence of 543 and 530 MYA and maximum age of Mollusk crown group divergence of 549 MYA.

Using single-copy orthologs, we could probe the phylogenetic relationships for *A. fulica* and other mollusks. To this end, protein sequences of single-copy genes were aligned using CLUSTALX2.0 (Clustal X, RRID:SCR_017055) [66]. Guided by the protein multi-sequence alignment, the alignment of the CDSs for those genes was generated and concatenated for the following analysis. The phylogenetic relationships were constructed using PhyML3.0 (PhyML, RRID:SCR_014629) [67] using the concatenated nucleotide alignment with the JTT+G+F model. The MCMCtree program in PAML4 [67] was used to estimate the species divergent time scales for the mollusks using the approximate likelihood method and calibrated according to the fossil records. We found that *A. fulica* was most closely related to *B. glabrata*, and the 2 species diverged from their common ancestor ∼243 million years ago (MYA) (Fig. 4).

## Conclusion

We reconstructed the first chromosome-level assembly for *A. fulica* using an integrated sequencing strategy combining PacBio, Illumina, and Hi-C technologies. Using the long reads from the PacBio Sequel platform and short reads from the Illumina X Ten platform, we successfully constructed a contig assembly for *A. fulica*. Leveraging contact information among contigs from Hi-C technology, we further improved the assembly to near chromosome-level quality (Table 3 and Fig. 2). We predicted 23,726 protein-coding genes in the *A. fulica* genome, and 22,858 of the genes were functionally annotated with putative functions. With 675 single-copy orthologs from *A. fulica* and other related mollusks, we constructed the phylogenetic relationship of these mollusks and found that *A. fulica* might have diverged from its common ancestor *B. glabrata* ∼177.1–187.1 MYA. Given the increasing interest in mollusk genomic evolution and the biological importance of *A. fulica* as an invasive animal, our genomic and transcriptome data will provide valuable genetic resources for follow-on functional genomics investigations by the research community.

## Ethics Statement

This study was approved by the Animal Care and Use committee of the National Institute of Parasitic Diseases, Chinese Center for Disease Control and Prevention.

## Availability of Supporting Data and Materials

The Illumina, PacBio, and Hi-C sequencing data are available from NCBI via the accession numbers SRR8369706, SRR8369311, and SRR8371669, respectively. The Illumina transcriptome sequencing data were deposited to NCBI via the accession numbers SRR8371872 and SRR8371873. The genome, annotation, and intermediate files have been uploaded to the*GigaScience* GigaDB Database [68].

## Additional Files

**Supplemental Table S1**. Summary of RNA quality of samples. The high-quality samples are highlighted in red for the PacBio library construction and sequencing.

**Supplemental Figure S1**. Bioanalyzer summary reports for samples used in the transcriptome sequencing.

**Supplemental Figure S2**. The distribution of *k*-mer species estimated for *A. fulica*. The total number of *k*-mer species is 178,847,565,204, with the peak value (depth) being 76.

giz124_GIGA-D-19-00006_Original_Submission

giz124_GIGA-D-19-00006_Revision_1

giz124_GIGA-D-19-00006_Revision_2

giz124_GIGA-D-19-00006_Revision_3

giz124_Response_to_Reviewer_Comments_Original_Submission

giz124_Response_to_Reviewer_Comments_Revision_1

giz124_Response_to_Reviewer_Comments_Revision_2

giz124_Reviewer_1_Report_Original_Submission Takeshi Takeuchi, Ph.D -- 2/7/2019 Reviewed

giz124_Reviewer_1_Report_Revision_1 Takeshi Takeuchi, Ph.D -- 5/31/2019 Reviewed

giz124_Reviewer_1_Report_Revision_2 Takeshi Takeuchi, Ph.D -- 7/17/2019 Reviewed

giz124_Reviewer_2_Report_Original_Submission Reuben William Nowell, Ph.D -- 2/18/2019 Reviewed

giz124_Reviewer_2_Report_Revision_1 Reuben William Nowell, Ph.D. -- 6/3/2019 Reviewed

giz124_Reviewer_3_Report_Original_Submission Marcela Uliano da Silva, Ph.D -- 2/25/2019 Reviewed

giz124_Supplemental_File

## Abbreviations

BLAST: Basic Local Alignment Search Tool; BUSCO: Benchmarking Universal Single-Copy Orthologs; BWA: Burrows-Wheeler Aligner; cDNA: complementary DNA; CDS: coding DNA sequence; FLNC: full-length non-chimeric sequence; Gb: gigabase pairs; GCE: Genomic Character Estimator; GO: gene ontology; kb: kilobase pairs; KEGG: Kyoto Encyclopedia of Genes and Genomes; Mb: megabase pairs; MYA: million years ago; NCBI: National Center for Biotechnology Information; PacBio: Pacific Biosciences; SMRT: Single-Molecule Real-Time; TE: transposable element; TrEMBL: Translation of European Molecular Biology Laboratory.

## Competing Interests

The authors declare that they have no competing interests.

## Funding

This work was supported by the National Key Research and Development Program of China (Nos. 2016YFC1200500 and 2016YFC1202000).

## Authors' Contributions

Y.G., X.Z., W.H. and N.X. conceived the project; Y.G., Y.Z., Y.H., G.M., Z.Y., E.A. and J.L. collected the samples and extracted the genomic DNA. Y.G., Y.Z., Q.L., Z.W. and S.L. performed the genome assembly and data analysis. Y.G., X.Z., W.H., and N.X. wrote the manuscript. W.H. and N.X. read and approved the final version of the manuscript.
