# Supplementary material for: A chromosomal-level genome assembly for the giant African snail Achatina fulica
Source: Gigascience. 2019 Oct 21;8(10):giz124. doi: 10.1093/gigascience/giz124 (PMC6802634; doi:10.1093/gigascience/giz124)
Supplement: giz124_GIGA-D-19-00006_Original_Submission [file giz124_giga-d-19-00006_original_submission.pdf]

GigaScience

A chromosomal-level genome assembly for the giant African snail Achatina fulica

--Manuscript Draft--

**Manuscript Number:**	GIGA-D-19-00006
**Full Title:**	A chromosomal-level genome assembly for the giant African snail Achatina fulica
**Article Type:**	Data Note
**Funding Information:**	This work was supported by the National Key Research and Development Program of China (No. 2016YFC1200500 and 2016YFC1202000) — Dr Ning Xiao
**Abstract:**	Background:<br>Achatina fulica (A. fulica), also called giant African snail, is the largest species in the reported terrestrial mollusks. Due to its greedy appetite, wide environmental adaptability, high growth rate and reproduction capacity, the species caused world-wide invasion, mainly in Southeast Asia, Japan, the western Pacific islands and China. A. fulica is a pest to damage the agricultural crops, as well as an intermediate host of many parasites to threaten human health. However, genomic information of A. fulica is still limited, hindering the genetic and genomic studies with the aim to invasion control and management of the species.<br><br>Finding:<br>Using Kmer-based method, we estimated the A. fulica genome size of 2.12 Gb with a high repeat content up to 71%. About 101.6 Gb genomic long-read data of A. fulica were generated from the PacBio sequencing platform and assembled to the first A. fulica genome of 1.85 Gb with a contig N50 length of 726 kb. Using contact information from the Hi-C sequencing data, we successfully anchored 99.32% contig sequences into 31 chromosomes, leading to the final contig and scaffold N50 length of 721 kb and 59.6 Mb, respectively. The continuity, completeness and accuracy were evaluated by genome comparison with other mollusk genomes, BUSCO assessment and genomic read mapping. 23,726 protein-coding genes were predicted from the assembled genome, among which 96.34% of the genes were functionally annotated. The phylogenetic analysis using whole-genome protein-coding genes revealed that A. fulica separated from the common ancestor with Biomphalaria glabrata around 182 million years ago.<br><br>Conclusion:<br>As our best knowledge, the A. fulica genome was the first terrestrial mollusk genome reported so far. The chromosome sequences of A. fulica will not only provided the research community valuable genome resource for the population genetics and environmental adaptation studies for the species, as well as, for the chromosome level evolution investigation with other mollusks.<br><br>Key Words: Giant African snail, Achatina fulica, PacBio, Hi-C, chromosome assembly
**Corresponding Author:**	ning xiao<br><br>CHINA
**Corresponding Author Secondary Information:**	
**Corresponding Author's Institution:**	
**Corresponding Author's Secondary Institution:**	
**First Author:**	Yunhai Guo
**First Author Secondary Information:**	
**Order of Authors:**	Yunhai Guo

Powered by Editorial Manager® and ProduXion Manager® from Aries Systems Corporation

	Yi Zhang
	Qin Liu
	Yun Huang
	Guangyao Mao
	Zhiyuan Yue
	Eniola M. Abe
	Jian Li
	Zhongdao Wu
	Shizhu Li
	Xiaonong Zhou
	Wei Hu
	Ning Xiao
**Order of Authors Secondary Information:**	
**Additional Information:**	
**Question**	**Response**
Are you submitting this manuscript to a special series or article collection?	No
**Experimental design and statistics**<br><br>Full details of the experimental design and statistical methods used should be given in the Methods section, as detailed in our Minimum Standards Reporting Checklist. Information essential to interpreting the data presented should be made available in the figure legends.<br><br>Have you included all the information requested in your manuscript?	Yes
**Resources**<br><br>A description of all resources used, including antibodies, cell lines, animals and software tools, with enough information to allow them to be uniquely identified, should be included in the Methods section. Authors are strongly encouraged to cite Research Resource Identifiers (RRIDs) for antibodies, model organisms and tools, where possible.	Yes

Powered by Editorial Manager® and ProduXion Manager® from Aries Systems Corporation

Have you included the information requested as detailed in our Minimum Standards Reporting Checklist?	
**Availability of data and materials**<br><br>All datasets and code on which the conclusions of the paper rely must be either included in your submission or deposited in publicly available repositories (where available and ethically appropriate), referencing such data using a unique identifier in the references and in the “Availability of Data and Materials” section of your manuscript.<br><br>Have you have met the above requirement as detailed in our Minimum Standards Reporting Checklist?	Yes

Manuscript
Click here to download Manuscript 20190109_Achatina_fulica_genome_manuscript.doc

A chromosomal-level genome assembly for the giant African snail *Achatina fulica*

Guo Yunhai[1,2,#], Zhang Yi[1,2,#], Liu Qin[1,2], Huang Yun[1,2], Mao Guangyao[1,2], Yue Zhiyuan[1,2], Eniola M. Abe[1,2], Li Jian[3], Wu Zhongdao[4], Li Shizhu[1,2], Zhou Xiaonong[1,2], Hu Wei[1,2,3,*], Xiao Ning[1,2,*]

[1]National Institute of Parasitic Diseases, Chinese Center for Disease Control and Prevention

[2]Key Laboratory of Parasite and Vector Biology, Ministry of Health, Shanghai, China

[3]Department of Microbiology and Microbial Engineering , School of Life Sciences , Fudan University , Shanghai 200438 , China

[4]Department of Parasitology, Zhongshan School of Medicine, Sun Yat-sen University, Guangzhou 510080, China

Abstract

Background:

Achatina fulica (A. fulica), also called giant African snail, is the largest species in the reported terrestrial mollusks. Due to its greedy appetite, wide environmental adaptability, high growth rate and reproduction capacity, the species caused world-wide invasion, mainly in Southeast Asia, Japan, the western Pacific islands and China. *A. fulica* is a pest to damage the agricultural crops, as well as an intermediate host of many parasites to threaten human health. However, genomic information of *A. fulica* is still limited, hindering the genetic and genomic studies with the aim to invasion control and management of the species.

Finding:

Using *K*mer-based method, we estimated the *A. fulica* genome size of 2.12 Gb with a high repeat content up to 71%. About 101.6 Gb genomic long-read data of *A. fulica* were generated from the PacBio sequencing platform and assembled to the first *A. fulica* genome of 1.85 Gb with a contig N50 length of 726 kb. Using contact information from the Hi-C sequencing data, we successfully anchored 99.32% contig sequences into 31 chromosomes, leading to the final contig and scaffold N50 length of 721 kb and 59.6 Mb, respectively. The continuity, completeness and accuracy were evaluated by genome comparison with other mollusk genomes, BUSCO assessment and genomic read mapping. 23,726 protein-coding genes were predicted from the assembled genome, among which 96.34% of the genes were functionally annotated. The phylogenetic analysis using whole-genome protein-coding genes revealed that *A. fulica* separated from the common ancestor with *Biomphalaria glabrata* around 182 million years ago.

Conclusion:

As our best knowledge, the *A. fulica* genome was the first terrestrial mollusk genome reported so far. The chromosome sequences of *A. fulica* will not only provided the research community valuable genome resource for the population genetics and environmental adaptation studies for the species, as well as, for the chromosome level evolution investigation with other mollusks.

Key Words: Giant African snail, *Achatina fulica*, PacBio, Hi-C, chromosome assembly

Data description

Introduction

The giant African snail, *A. fulica*, is a Gastropod species (**Figure 1**). It is the largest interrestrial mollusks with greedy appetite, strong environmental adaptability, and high growth and reproduction rate[1-3]. Originating from East Africa, *A. fulica* gradually invaded Southeast Asia, Japan and the western Pacific islands in the last century[4-6] with the direct or indirect help fromhumans[7-9].In mainland China, the first *A. fulica* invasion event was reported in 1931[10]. At present, the snail's natural distribution in the wild has been found in Guangdong, Hainan, Guangxi, southern parts of Yunnan Province and Fujian Province, and a county of Guizhou Province[11]. *A. fulica* was included as the first 16 alien invasive species in China (http://www.mee.gov.cn/gkml/zj/wj/200910/t20091022_172155.htm) in 2003, and was also listed by International Union for Conservation of Nature (IUCN) as the 100 most threatening alien invasive species[12]. This snail has been recognized as an agricultural and garden pest that has caused significant damages in both tropical and subtropical regions[9,12,13]. In addition, *A. fulica* is also the intermediate host of *Angiostrongyl cantonensis*. Human infection with angiostrongyliasis, which occurs mainly through consumption of snails carrying *A. cantonensis* larvae, causes eosinophilic meningoencephalitis[4,11,14-18]. As a consequence, *A. fulica* is attracting more and more attention in fields of both agricultural crops protection and human disease control.

To date, a variety of mollusk genomes have been analyzed and published, including two freshwater gastropods snails *Pomacea canaliculata*[19] and *Biomphalaria glabrata*[20]. However, no genome has been reported for terrestrial mollusks. *A. fulica* is considered to be one of the most serious threat and a destructive terrestrial gastropod which poses a significant hazard to agriculture, the environment, biodiversity and human health. In this work, we applied Illumina, PacBio and Hi-C techniques to construct the chromosome of *A. fulica*. The genome is the first terrestrial mollusk

genome, providing an important reference for the molecular mechanism investigations for its broad environmental adaptability and the development of control strategy of the world-wide invasion.

Sample and sequencing

An adult snail (**Figure 1**), which was collected in Pingxiang city, Guangxi Autonomous Region, was used for reference genome construction. The snail was dissected and abdominal foot (17.4 g) and liver pancreas (40.4 g) tissues were collected and quickly frozen in liquid nitrogen overnight before transferring to -80 °C for storage. DNA was extracted using the traditional phenol/chloroform extraction method and was quality checked using agarose gel electrophoresis, meeting the requirement for library construction for the Illumina X Ten (Illumina Inc., San Diego, CA, USA) and for the PacBio Sequel (Pacific Biosciences of California, Menlo Park, CA, USA) sequencing platforms.

Using the DNA molecules from abdominal foot, a library with the insertion length of 300 bp were constructed and sequenced for Illumina sequencing platform according to the manufacturer's protocol. About 195.4 Gb short reads were obtained from the Illumina X Ten sequencing technology (**Table 1**), which was used for the following genome survey analysis, and for final base-level genome sequence correction. Meanwhile, four 20 kb libraries were constructed for PacBio Sequel sequencing. Using 16 sequencing SMRT cells, 101.6 Gb long reads were generated (**Table 1**). The mean and N50 lengths of the polymerases for sequencing cells ranged from 6.4 kb to 10.4 kb and from 12.3 kb to 20.3 kb for cells, respectively. Those long genomic DNA reads were used for reference genome construction.

Genome features estimation from *K*mer method

With sequencing data from the Illumina platform, several genome characters could be evaluated form *A. fulica.* To ensure the quality of the analysis, ambiguous bases and low-quality reads were trimmed and filtered using the HTQC package[21]. The following

quality control were performed under the framework of HTQC. First, the quality of bases at two read ends were checked. Bases in sliding 5 bp windows were deleted if the average quality of the window was below phred quality score of 20. Second, reads were filtered if the average phred quality score were smaller than 20 or the read length was shorter than 75 bp. Third, the mate reads were also removed if the corresponding reads were filtered.

The quality-controlled reads were used for genome character estimation. We calculated the number of each 17-mer from the sequencing data using the jellyfish software[22], and the distribution was analyzed with GCE software[23]. We estimated the genome size of 2.12 Gb with the heterozygosity of 0.47% and repeat content of 71% in the genome. Previous studies revealed that repeat content varies in mollusks, and that repeat content is correlated with genome size[24]. The large genome size and high proportion of repeat contents of *A. fulica* provided additional supporting data for the statically analysis.

Genome assembly by third-generation long reads

After removing adaptor sequences in polymerases, 101.6 Gb subreads were generated for the following whole genome assembly. The average and N50 length of subreads reached 5.25 kb and 8.80 kb, respectively. To optimize the genome assembly using the PacBio sequencing data, we applied two packages in the assembly process, Canu[25] and FALCON[26]. Canu package was first applied for the assembly with the default parameters. As a result, a 1.93 Gb genome was constructed with 10,417 contigs and a contig N50 length of 662.40 kb. FALCON was also employed using the length_cutoff and pr_length_cutoff parameters of 10 kb and 8 kb, respectively. We obtained 1.85 Gb genome with 8,585 contigs, with a contig N50 of 726.63 kb. We adopted the FALCON assembly as the reference genome for *A. fulica* (**Table 2**). The genome sequences were subsequently polished by PacBio long reads using arrow[27] and Illumina short reads by pilon[28] to correct base errors. The

corrected genome was further applied for the following chromosome assembly construction using Hi-C data.

In situ Hi-C library construction and chromosome assembly using Hi-C data

Liver pancreas tissue of *A. fulica* was used for library construction for Hi-C analysis and the library was constructed using the identical method in previous studies[29]. Finally, the library was sequenced with 150 paired-end mode on the Illumina HiSeq X Ten platform (San Diego, CA, United States). From the Illumina sequencing platform, 1,313 million paired-end reads were obtained for the Hi-C library (**Table 1**). The reads were mapped to the above *A. fulica* genome with Bowtie[30], with two ends of paired reads being mapped to the genome separately. To increase the interactive Hi-C reads ratio, an iterative mapping strategy was performed as previous studies, and only read pairs that both ends uniquely mapped were used for the following analysis. From the alignment status of two ends, self-ligation, non-ligation and other sorts of invalid reads, including StartNearRsite, PCR amplification, random break, LargeSmallFragments and ExtremeFragments, were filtered out by Hi-Clib[31]. Through the recognition of restriction sites in sequences, contact counts among contigs were calculated and normalized.

According to previous karyotype analyses, *A. fulica* had 31 chromosomes[32]. By clustering the contigs using the contig contact frequency matrix, we were able to correct some minor errors in the FALCON assembly results. Contigs with errors were broken into shorter contigs. We obtained 8,701 contigs, slightly more than the 8,585 contigs in the FALCON assembly. We successfully clustered these contigs into 31 groups in Lachesis[33] using the agglomerative hierarchical clustering method (**Figure 2**). Lachesis was further applied to order and orient the clustered contigs according to the contact matrix. As a result, 7,106 contigs were reliably anchored, ordered and orientated on chromosomes, accounting for 99.32% of the total genome bases. Then,

we applied PBJelly[34] to fill the gap using PacBio long reads to merge the contig sequences. Finally, the first chromosomal-level assembly of *A. fulica* was obtained with 8,211 contigs, a contig N50 of 721.0 kb and a scaffold N50 of 59.59 Mb (**Table 2** and **Figure 3**).

Genome quality evaluation

We assessed the quality of genome of *A. fulica* after the assembly process. The quality evaluation was carried out in three aspects: continuity, completeness and base level accuracy.

First of all, we compared the sequence number and N50 length of contig of *A. fulica* with public genome of mollusks and found that our assembly has a high quality on contig and scaffold N50 among mollusk genomes. (**Figure 3**) As previous studies, genomic heterozygosity of mollusk was one of the biggest challenges for genome assembly, both in terms of contig and scaffold assembly[35]. Our work illustrated that the genome assembly using PacBio long sequencing data was affordable and effective to overcome the difficulty of mollusk genome assembly. Traditional chromosomal genome assembly requires physical maps and genetic maps, which is enormously time-and labor-consuming. With Hi-C data analysis, we successfully assembled *A. fulica* genome into chromosome-level with just one individual.

Second, the assembled genome was subjected to the BUSCO (version 3.0)[36] to assess the completeness of the genome. 91.7% of the BUSCO genes were identified in *A. fulica* genome. More than 84.7% BUSCO gene were single-copy completed in our genome, illuminating a high level of completeness of the genome.

Third, NGS short reads were aligned to the genome using BWA package[37]. About 98.7% of paired reads were aligned to the genome, of which 98.24% were reads paired aligned. From the NGS reads alignment, we detected 128,998 homologous SNP loci using the GATK pipeline[38], demonstrating the high base-level accuracy of 99.33%.

Repeat element and gene annotation

Tandem Repeat Finder (TRF)[39] was used for repetitive element identification in the *A. fulica* genome. A *de novo* method applying RepeatModuler was used to detect transposable elements (TEs). The resulted *de novo* data, combined with known repeat library from Repbase[40], were used to identify TEs in the*A. fulica* genome by RepeatMasker[41] software. All repetitive elements were masked in the genome for the protein-coding gene prediction.

Protein-coding genes in the *A. fulica* genome were annotated using the *de novo* program Augustus[42]. Protein sequences of the closely related species including *Aplysia californica*, *Biomphalaria glabrata*，*Crassostrea gigas*，*Lottia gigantea* and *Patinopecten yessoensis*, were downloaded from the Ensembl database, and aligned to the *A. fulica* genome with TBLASTN. Full-length transcripts obtained using Iso-Seq were mapped to the genome using Genewise[43]. Finally, gene models predicted from all above methods were combined by MAKER[44], resulting in 23,726 protein-coding genes. The gene number, gene length, CDS length, exon length and intron length distribution were all comparable with the related mollusks (**Figure 4**).

To functionally annotate protein-coding genes in the *A. fulica* genome, we searched all predicted gene sequences to NCBI non-redundant nucleotide (NT) and protein (NR), Swiss-Prot databases by BLASTN[45] and BLASTX[46] utility. Blast2GO[47] was also used to assign gene ontology (GO)[48] and Kyoto Encyclopedia of Genes and Genomes (KEGG)[49] pathways. A threshold of e-value of 1e-5 was used for all BLAST applications. Finally, 22,858 (96.34%) genes were functionally annotated (**Table 3**).

Phylogenetic analysis of *A. fulica* with other mollusks

OrthoMCL[50] was used to cluster gene families. First, proteins from *A. fulica* and the closely related mollusks, including *Aplysia californica*, *Biomphalaria glabrata*, *Crassostrea gigas*, *Lingula anatina*, *Lottia gigantea*, *Patinopecten yessoensis*, *Octopus bimaculoides*, *Helobdella robusta*, *Drosophila melanogaster* and *Pomacea*

canaliculata were all-to-all blasted by BLASTP[46] utility with an e-value threshold of 1e-5. Only proteins from the longest transcript were used fro genes with alternative splices. We identified 25,448 gene families for *A. fulica* and the related species, among them 675 single-copy orthologs families were detected.

Using single-copy orthologs, we could probe the phylogenetic relationships for the *A. fulica* and other mollusks. To this end, protein sequences of single-copy genes were aligned using CLUSTALX[51]. Guided by the protein multi-sequence alignment, the alignment of the coding DNA sequences (CDS) for those genes were generated and concatenated for the following analysis. The phylogenetic relationships were constructed using PhyML[52] using the concatenated nucleotide alignment with the JTT+G+F model. The PAML MCMCtree program[52] was used to estimate the species divergent time scales for the mollusks using approximate likelihood method. We found that *A. fulica* was most closely related to *Biomphalaria glabrata*, and the two species diverged from their common ancestor around 177.1-187.1 million years ago (MYA) (**Figure 5**).

Conclusion

We reconstructed the first chromosome level assembly for *A. fulica* using an integrated strategy of PacBio, Illumina and Hi-C technologies. Using the long reads from PacBio Sequel platform and short reads from the Illumina X Ten platform, we successfully constructed contig assembly for *A. fulica*. Leveraging contact information among contigs from Hi-C technology, we further improved the assembly to the chromosome-level quality (**Figure 2** and **Figure 3**). We annotated 23,726 protein-coding genes in the *A. fulica* genome and 22,858 of genes were functionally annotated. With 675 single-copy orthologs from *A. fulica* and other related mollusks, we construct the phylogenetic relationship of these mollusks, and found that *A. fulica* might have diverged from its common ancestor of *Biomphalaria glabrata* around 177.1-187.1 MYA. Given the increasing interests in mollusk genomic evolution and

the biological importance of *A. fulica* as an invasive animal, our genomic and transcriptome data provide valuable genetic resource for the following functional genomics investigations for the research community.

Ethics Statement

This study was approved by the Animal Care and Use committee of National Institute of Parasitic Diseases, Chinese Center for Disease Control and Prevention. All participates consent the study under the 'Ethics, consent and permissions' heading. All participants consent to publish the work under the 'Consent to publish' heading.

Availability of supporting data

The Illumina, PacBio and Hi-C sequencing data are available from NCBI via the accession number ofSRR8369706, SRR8369311 and SRR8371669, respectively. The Illumina transcriptome sequencing data were deposited to NCBI via the accession number of SRR8371872 and SRR8371873. The genome, annotation and intermediate files were uploaded to GigaScience FTP server.

Competing interests

The authors declare that they have no competing interests.

Acknowledgement

This work was supported by the National Key Research and Development Program of China (No. 2016YFC1200500 and 2016YFC1202000). The authors thank Frasergen Bioinformatics for providing technique supports for this work.

Author Contributions

Z.X, H.W and X.N conceived the project. G.Y, Z.Y, L.Q collected the samples and extracted the genomic DNA. G.Y, Z.Y and L.Q performed the genome assembly and data analysis. G.Y, Z.X, H.W and X.N wrote the paper.

References

1 Schreurs, J. Investigations on the biology, ecology and control of Giant African Snail 290 in West New Guinea. (Manokwari Agricultural Research Station., 1963).

2 Albuquerque, F. S., Peso-Aguiar, M. C. & Assunção-Albuquerque, M. J. Distribution,feeding behavior and control strategies of the exotic land snail *Achatina fulica* (Gastropoda:Pulmonata) in the Northeast of Brazil. *Braz.J.Biol.* **68**, 6 (2008).

3 Thiengo, S. C., Fernandez, M. A., Torres, E. J., Coelho, P. M. & Lanfredi, R. M. First record of anematode Metastrongyloidea (Aelurostrongylus abstrusus larvae) in *Achatina* (Lissachatina) *fulica* (Mollusca,Achatinidae) in Brazil. *J. Invertebr. Pathol.* **98**, 6 (2008).

4 Lv, S., Zhang, Y. & Liu, H. X. Invasive Snails and an Emerging Infectious Disease: Results from the First National Survey on Angiostrongylus cantonensis in China. *BioOne*, doi:10.1371/journal.pntd.0000368 (2009).

5 Cowie, R. H. Non-indigenous land and freshwater molluscs in the islands of the Pacific: Conservation impacts and threats. 143-172 (2000).

6 Cowie, R. H. Can snails ever be effective and safe biocontrol agents? *Int J Pest Manage* **47**, 18 (2001).

7 Cowie, R. H. & Robinson, D. G. *Pathways of introduction of nonindigenous land and freshwater snails and slugs*. 93-122 (Island Press, 2003).

8 Kotangale, J. P. Giant African snail (*Achatina fulica* Bowdich). **J Environ Sci Eng**, 6 (2011).

9 Raut, S. K. & Barker, G. M. *Achatina fulica Bowdich and Other Achatinidae as Pests in Tropical Agriculture*. (CABI International, 2002).

10 Jarreit, V. H. C. The spread of the snail *Achatina fulica* to south China. *Hong Kong Nat* **2**, 3 (1931).

11 Shan, L., Yi, Z. & Peter, S. Emerging Angiostrongyliasis in Mainland China. *Emerging Infectious Diseases* **14**, 4 (2008).

12 Lowe, S., Browne, S. M., Boudjrlas, S. & De Poorter, M. *100 of the world's worst invasive alien species: A selection from the global invasive species database. The Invasive Species Specialists Group of the Species Survival Commission of the World Conservation Union.*, (Hollands Printing, 2000).

13 Mead, A. R. *Pulmonates volume 2B.* Economic malacology with particular reference to *Achatina fulica*. (Academic Press, 1979).

14 Alicata, J. E. The discovery of Angiostrongylus cantonensis as a cause of human eosinophilic meningitis. *Parasitol Today* **7**, 151-153 (1991).

15 Prociv, P., Spratt, D. M. & Carlisle, M. S. Neuro-angiostrongyliasis: unresolved issues. *Int J Parasitol* **30**, 1295-1303 (2000).

16 Deng, Z. H., Zhang, Q. M., Huang, S. Y. & Jones, J. L. First provincial survey of Angiostrongylus cantonensis in Guangdong Province, China. *Trop Med Int Health* **17**, 4 (2012).

17 Maldonado, J. A. *et al.* First report of Angiostrongylus cantonensis (Nematoda: Metastrongylidae) in *Achatina fulica* (Mollusca: Gastropoda) from Southeast and South Brazil. *Mem Inst Oswaldo Cruz* **105**, 4 (2010).

18 Vitta, A., Polseela, R., Nateeworanart, S. & Tattiyapong, M. Survey of Angiostrongylus cantonensis in rats and giant African land snails in Phitsanulok Province, Thailand. *Asian Pac J Trop Med* **4**, 3 (2011).

19 Liu, C. *et al.* The genome of the golden apple snail *Pomacea canaliculata* provides insight into

stress tolerance and invasive adaptation. *Gigascience* **7**, doi:10.1093/gigascience/giy101 (2018).

20 Adema, C. M. *et al.* Whole genome analysis of a schistosomiasis-transmitting freshwater snail. *Nature communications* **8**, 15451, doi:10.1038/ncomms15451 (2017).

21 Neff, K. L. *et al.* Mojo Hand, a TALEN design tool for genome editing applications. *BMC Bioinformatics* **14**, 1, doi:10.1186/1471-2105-14-1 (2013).

22 Marcais, G. & Kingsford, C. A fast, lock-free approach for efficient parallel counting of occurrences of k-mers. *Bioinformatics* **27**, 764-770, doi:10.1093/bioinformatics/btr011 (2011).

23 Binghang Liu, Y. S., Jianying Yuan,Xuesong Hu,Hao Zhang,Nan Li,Zhenyu Li,Yanxiang Chen,Desheng Mu,Wei Fan. Estimation of genomic characteristics by analyzing k-mer frequency in de novo genome projects. *Quantitative Biology* **35**, 62-67 (2013).

24 Murgarella, M. *et al.* A First Insight into the Genome of the Filter-Feeder Mussel Mytilus galloprovincialis. *PLoS One* **11**, e0151561, doi:10.1371/journal.pone.0151561 (2016).

25 Koren, S. *et al.* Canu: scalable and accurate long-read assembly via adaptive k-mer weighting and repeat separation. *Genome Res* **27**, 722-736, doi:10.1101/gr.215087.116 (2017).

26 Chin, C. S. *et al.* Phased diploid genome assembly with single-molecule real-time sequencing. *Nat Methods* **13**, 1050-1054, doi:10.1038/nmeth.4035 (2016).

27 Chin, C.-S. *et al.* Nonhybrid, finished microbial genome assemblies from long-read SMRT sequencing data. *Nature methods* **10**, 563 (2013).

28 Walker, B. J. *et al.* Pilon: an integrated tool for comprehensive microbial variant detection and genome assembly improvement. *PloS one* **9**, e112963 (2014).

29 Gong, G. *et al.* Chromosomal-level assembly of yellow catfish genome using third-generation DNA sequencing and Hi-C analysis. *Gigascience*, doi:10.1093/gigascience/giy120 (2018).

30 Langmead, B., Trapnell, C., Pop, M. & Salzberg, S. L. Ultrafast and memory-efficient alignment of short DNA sequences to the human genome. *Genome Biol* **10**, R25, doi:10.1186/gb-2009-10-3-r25 (2009).

31 Burton, J. N. *et al.* Chromosome-scale scaffolding of de novo genome assemblies based on chromatin interactions. *Nature biotechnology* **31**, 1119 (2013).

32 Sun, T. Chromosomal studies in three land snails. *Sinozoologia* **12**, 154-162 (1995).

33 Near, T. J. *et al.* Phylogeny and tempo of diversification in the superradiation of spiny-rayed fishes. *Proceedings of the National Academy of Sciences of the United States of America* **110**, 12738 (2013).

34 English, A. C. *et al.* Mind the gap: upgrading genomes with Pacific Biosciences RS long-read sequencing technology. *PloS one* **7**, e47768 (2012).

35 Zhang, G. *et al.* The oyster genome reveals stress adaptation and complexity of shell formation. *Nature* **490**, 49 (2012).

36 Simão, F. A., Waterhouse, R. M., Ioannidis, P., Kriventseva, E. V. & Zdobnov, E. M. BUSCO: assessing genome assembly and annotation completeness with single-copy orthologs. *Bioinformatics* **31**, 3210-3212 (2015).

37 Li, H. & Durbin, R. Fast and accurate short read alignment with Burrows–Wheeler transform. *bioinformatics* **25**, 1754-1760 (2009).

38 McKenna, A. *et al.* The Genome Analysis Toolkit: a MapReduce framework for analyzing next-generation DNA sequencing data. *Genome research* (2010).

39 Benson, G. Tandem repeats finder: a program to analyze DNA sequences. *Nucleic Acids Res* **27**, 573-580 (1999).

40 Bao, W., Kojima, K. K. & Kohany, O. Repbase Update, a database of repetitive elements in eukaryotic genomes. *Mob DNA* **6**, 11, doi:10.1186/s13100-015-0041-9 (2015).

41 Chen, N. Using RepeatMasker to identify repetitive elements in genomic sequences. *Current protocols in bioinformatics* **5**, 4.10. 11-14.10. 14 (2004).

42 Stanke, M. *et al.* AUGUSTUS: ab initio prediction of alternative transcripts. *Nucleic acids research* **34**, W435-W439 (2006).

43 Birney, E., Clamp, M. & Durbin, R. GeneWise and genomewise. *Genome research* **14**, 988-995 (2004).

44 Cantarel, B. L. *et al.* MAKER: an easy-to-use annotation pipeline designed for emerging model organism genomes. *Genome research* **18**, 188-196 (2008).

45 Gertz, E. M., Yu, Y. K., Agarwala, R., Schaffer, A. A. & Altschul, S. F. Composition-based statistics and translated nucleotide searches: improving the TBLASTN module of BLAST. *BMC Biol* **4**, 41, doi:10.1186/1741-7007-4-41 (2006).

46 Camacho, C. *et al.* BLAST+: architecture and applications. *BMC Bioinformatics* **10**, 421, doi:10.1186/1471-2105-10-421 (2009).

47 Conesa, A. *et al.* Blast2GO: a universal tool for annotation, visualization and analysis in functional genomics research. *Bioinformatics* **21**, 3674-3676 (2005).

48 Consortium, G. O. The Gene Ontology (GO) database and informatics resource. *Nucleic acids research* **32**, D258-D261 (2004).

49 Kanehisa, M. & Goto, S. KEGG: kyoto encyclopedia of genes and genomes. *Nucleic acids research* **28**, 27-30 (2000).

50 Li, L., Stoeckert, C. J. & Roos, D. S. OrthoMCL: identification of ortholog groups for eukaryotic genomes. *Genome research* **13**, 2178-2189 (2003).

51 Thompson, J. D., Gibson, T. J. & Higgins, D. G. Multiple sequence alignment using ClustalW and ClustalX. *Current protocols in bioinformatics*, 2.3. 1-2.3. 22 (2003).

52 Guindon, S., Lethiec, F., Duroux, P. & Gascuel, O. PHYML Online—a web server for fast maximum likelihood-based phylogenetic inference. *Nucleic acids research* **33**, W557-W559 (2005).

Tables and Figures

Table 1: Sequencing data generated for *A.fulica* genome assembly and annotation

Source	Library type	Platform	Library size (bp)	Data size (Gb)	Application
Genome	Short reads	HiSeq X Ten	350	195.4	Genome survey and base correction
	Long reads	PacBio SEQUEL	20,000	101.6	Genome assembly
	Hi-C	HiSeq X Ten	300-500	208.9	Chromosome construction
Transcriptome	Long reads	PacBio SEQUEL	3000, 5000	22.5	Genome annotation

Table 2: Statistics for genome assembly of *A. fulica*

Sample ID	Length		Number	
	Contig** (bp)	Scaffold (bp)	Contig**	Scaffold
Total	1,852,282,574	1,855,883,074	8,211	1,010
Max	5,947,392	116,558,012	-	-
N50	721,038	59,589,303	697	13
N60	538,883	58,013,356	995	16
N70	399,612	53,672,006	1,396	20
N80	268,901	50,673,968	1,957	23
N90	141,756	44,109,545	2,888	27

Table 3: Statistics for genome annotation of *A. fulica*

Database	Number	Percent
InterPro	16,252	68.50
GO	12,101	51.00
KEGG ALL	21,325	89.88
KEGG KO	10,161	42.83
Swissprot	17,050	71.86
TrEMBL	22,403	94.42
NR	22,553	95.06
Total	23,726	

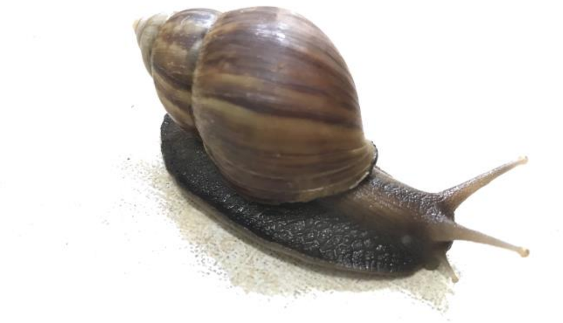

Figure 1. A picture of *A. fulica* that used for genome sequencing and assembly.

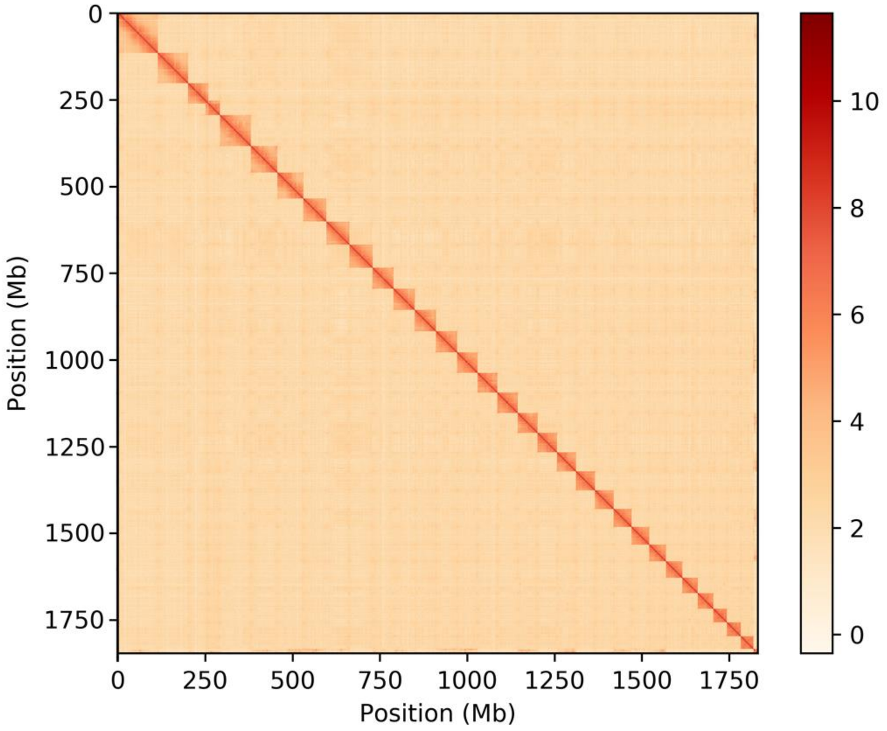

Figure 2. Contact matrix generated from the Hi-C data analysis showing sequence interactions in chromosomes. The logarithm of the contact density was showed in the color bar.

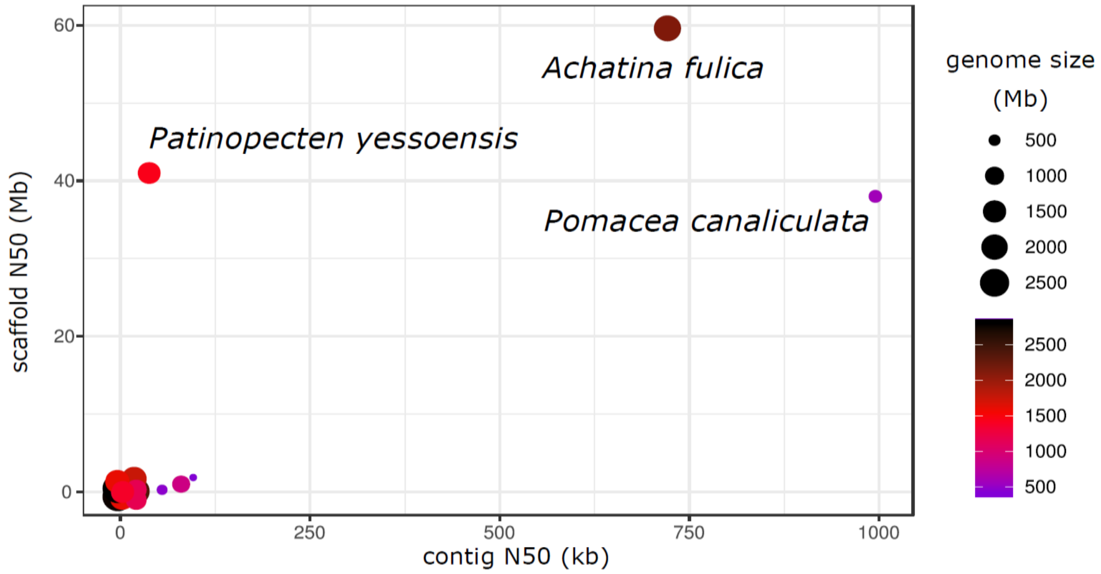

Figure 3: Genome assembly comparison of *A. fulica* with other sequenced mollusk genomes. The x- and y-axis represent the contig and scaffold N50s, respectively. The genomes assembled into chromosomal level are labeled with names.

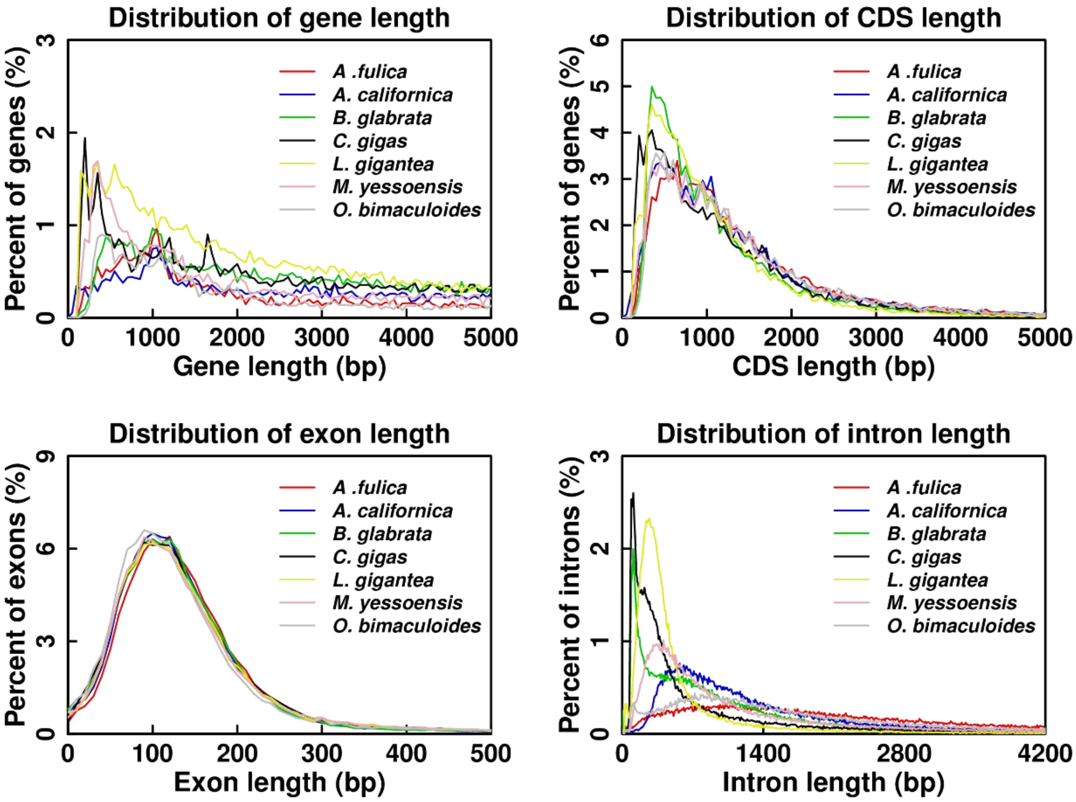

Figure 4. Length distribution comparison on total gene, CDS, exon, and intron of annotated gene models of *A. fulica* with other closely related species. The comparison of length distribution of genes (A), CDS (B), exon (C) and intron (D) for *A. fulica* to those in *A.californica* , *B. glabrata* , *C. gigas* , *L. gigantea* , *P. yessoensis* and *O. bimaculoides*.

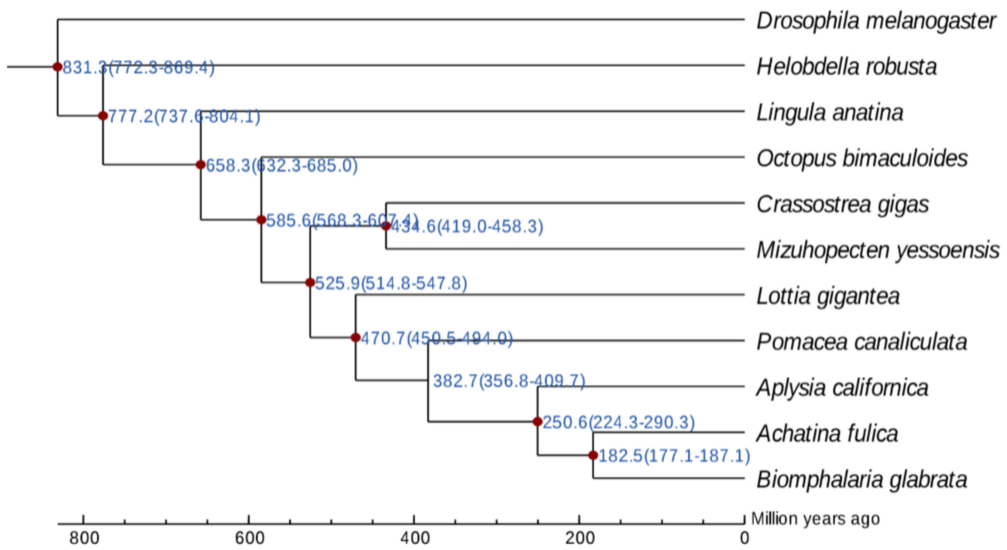

Figure 5. Phylogenetic relationship between *A. fulica* and related species. The divergence time (million years ago) and the 95% confidential intervals are labeled at branch sites and the red dots in the tree illuminated the speciation for the time recalibration.
