# Supplementary material for: A chromosomal-level genome assembly for the giant African snail Achatina fulica
Source: Gigascience. 2019 Oct 21;8(10):giz124. doi: 10.1093/gigascience/giz124 (PMC6802634; doi:10.1093/gigascience/giz124)
Supplement: giz124_GIGA-D-19-00006_Revision_1 [file giz124_giga-d-19-00006_revision_1.pdf]

GigaScience

A chromosomal-level genome assembly for the giant African snail Achatina fulica

--Manuscript Draft--

**Manuscript Number:**	GIGA-D-19-00006R1
**Full Title:**	A chromosomal-level genome assembly for the giant African snail Achatina fulica
**Article Type:**	Data Note
**Funding Information:**	This work was supported by the National Key Research and Development Program of China (No. 2016YFC1200500 and 2016YFC1202000) — Dr Ning Xiao
**Abstract:**	Background:<br>Achatina fulica (A. fulica), also called the giant African snail, is the largest species in the reported terrestrial mollusks. Due to its voracious appetite, wide environmental adaptability, high growth rate and reproductive capacity, the species caused a world-wide invasion, mainly in Southeast Asia, Japan, the western Pacific islands and China. A. fulica is a pest that is able to damage agricultural crops, as well as an intermediate host of many parasites that can threaten human health. However, genomic information of A. fulica is still limited, hindering genetic and genomic studies with the aim to invasion control and management of the species.<br>Finding:<br>Using Kmer-based method, we estimated the A. fulica genome size to be 2.12 Gb with a high repeat content up to 71%. About 101.6 Gb genomic long-read data of A. fulica were generated from the PacBio sequencing platform and assembled to the first A. fulica genome of 1.85 Gb with a contig N50 length of 726 kb. Using contact information from the Hi-C sequencing data, we successfully anchored 99.32% contig sequences into 31 chromosomes, leading to the final contig and scaffold N50 length of 721 kb and 59.6 Mb, respectively. The continuity, completeness and accuracy were evaluated by genome comparison with other mollusk genomes, BUSCO assessment and genomic read mapping. 23,726 protein-coding genes were predicted from the assembled genome, among which 96.34% of the genes were functionally annotated. The phylogenetic analysis using whole-genome protein-coding genes revealed that A. fulica separated from the common ancestor with Biomphalaria glabrata around 182 million years ago.<br>Conclusion:<br>As our best knowledge, the A. fulica genome was the first terrestrial mollusk genome reported so far. The chromosome sequences of A. fulica will provide the research community a valuable resource for the population genetics and environmental adaptation studies for the species, and furthermore, for the chromosome level of evolution investigation within mollusks.
**Corresponding Author:**	ning xiao<br><br>CHINA
**Corresponding Author Secondary Information:**	
**Corresponding Author's Institution:**	
**Corresponding Author's Secondary Institution:**	
**First Author:**	Yunhai Guo
**First Author Secondary Information:**	
**Order of Authors:**	Yunhai Guo
	Yi Zhang
	Qin Liu
	Yun Huang

Powered by Editorial Manager® and ProduXion Manager® from Aries Systems Corporation

	Guangyao Mao
	Zhiyuan Yue
	Eniola M. Abe
	Jian Li
	Zhongdao Wu
	Shizhu Li
	Xiaonong Zhou
	Wei Hu
	Ning Xiao
**Order of Authors Secondary Information:**	
**Response to Reviewers:**	Reviewer reports:<br><br>Reviewer #1: In this study the authors sequenced the genome of the giant African snail Achatina fulica using short and long read technologies as well as a Hi-C scaffolding method, and succeeded to develop chromosomal-level genome assembly. I think the data will contribute to our understanding of the biology of the species.<br>Reply: We thanks a lot for the reviewer's positive comments for our manuscript.<br><br>At the same time I found description of methods is not sufficient in the present manuscript, therefore it should be revised before publication.<br>In the Introduction the authors mentioned that it is important to study the biology of A. chatina because the species is one of the most threatening invasive species, and is the intermediate host of Angiostrongylus. However, I could not find how the present chromosomal-level genome assembly is useful to address these issues. I would like to request the authors to discuss the point more specifically. This will emphasize the importance of the study.<br>Reply: Thanks a lot for the suggestion. The chromosome genome of A. chatina could provide an important framework in the following population genetics using next-generation sequencing data. Meanwhile, the predicted genes in the genome of A. chatina could be used for the transcriptome analysis for the interaction of Angiostrongylus and A. chatina. We have added the information into the revised manuscript. (lines 85-91 in the revised ms)<br><br>The information about transcriptome is absent despite the data might be used for gene model prediction (lines 206-207). The authors should describe in detail about the transciptome. For example, from which tissues was RNA extracted? How was the quality of the RNA? How was the stats of RNA-Seq (number of reads, average length, etc.)? In addition, mapping rate of the transcriptome to the genome assembly and gene models will be informative to evaluate the completeness of the assembly and model prediction, respectively.<br>Reply: Thanks for the reviewer's reminding. The detailed information for the RNA sequencing has been added in the revised manuscript. (lines 106-124 in the revised ms)<br><br>Lines 178-180<br>High rate of heterozygosity (>1%) have been reported in bivalve genomes (oysters, scallops, etc.) but not the case in gastropods.<br>Reply: Thanks for the reviewer's reminding. Previous genome study of Pomacea canaliculata, belonging to gastropods, revealed the high heterozygosity among 1%-2%. (doi: 10.1093/gigascience/giy101) To avoid the confusion, we have deleted the sentence in the manuscript.<br><br>Fig. 3<br>I would suggest to show the genome assembly comparison data in a table, not in a scatter plot.<br>In general, scatter plot is used to see the correlation between two variables. This figure is not adequate to compare genome assemblies because 1) correlation between contig and scaffold N50s is not meaningful 2) most of the dots are put at the lower left and

Powered by Editorial Manager® and ProduXion Manager® from Aries Systems Corporation

indistinguishable.
In addition, references should be cited when the authors used these genome data in the study.
Reply: Thanks a lot for the suggestion. We have changed the Figure 3 into Table 3 and added the references in the revised manuscript.

Lines 232-235, Fig. 5
What kinds of fossil record were used for molecular clock calibration? Honestly speaking, I cannot believe the result (Fig.5), showing Spiralia diverged from Ecdysozoa 831 Mya (200 million years before the Ediacaran Period).
Reply: Thank you very much for the reminding. However, we re-estimated the divergence time among these species using the records for Protostomia and Mollusca downloaded from www.timetree.org and obtained the similar results (the figure below was downloaded from the place). Thus we believe the results might be reliable. The new results and the calibration information were updated in the revised ms. (lines 258-261 and fig 5)

Version information of all software used are needed.
Reply: Thank you very much for the reminding. All the version information available has been added in the revised ms.
Reviewer #2: Please see attached Review.
Overall, this appears to be a well put together genome encompassing large amounts of data from different sources, including long reads from PacBio and additional scaffolding from Hi-C. It is quite well presented and I'm sure this work will be useful to the community as a genomics resource. Nonetheless there are a few issues that I'd like to see resolved before the manuscript can be accepted for publication or the assembly is released into the public repositories.

Major comments
Contamination. There is no mention in the text of filters for possible contamination from non-target organisms in the sequencing data. I consider such an analysis to be a vital and necessary component of any genome project, to eliminate (as much as possible) errors from contaminating sequencing reads in sequence databases. Tools such as Blobtools (https://drl.github.io/blobtools/) are easy to implement and are highly informative as to the quality of the raw data and the final genome.
Reply: Thanks for the reviewer's reminding. Actually we did the contamination analysis at the step Survey since the DNA samples in Survey and Assembly was identical. In the survey step, we randomly extracted 10,000 pairs of short reads, and compared them to the nt database, and find no obvious external contamination from other species. This method has been described elsewhere (https://doi.org/10.1016/j.molp.2014.12.011) and we did not mention it since it performed as expected. The result of contamination analysis has been added in the revised ms (lines 154-155 in the revised ms).

Kmer analysis. There is much discussion about estimation of genome size from kmer analysis, but there is no kmer spectra presented. I would find this figure much more informative and useful than some of the figures that are included (e.g. 2 and 3).
Reply: Thank you very much for your suggestions. The kmer spectra has been added in the revised version (Figure 2).

Heterozygosity. Related to the above point: how did the authors resolve any regions containing heterozygous sites in the assembly? E.g., divergent allelic regions that might be co-assembled and both present in the final scaffolds?
Reply: Thank you very much for you reminding. By mapping the subreads back to the genome, we estimated the sequencing depth for each region of the assembly and the results were shown below (the GC content were also shown, 10k window). It shows that the distribution of the depth is unimodal, which means that almost all sites were homozygous, actually the heterozygosity of the species is not very high (<0.5%). And if there are too much divergent allelic regions, two peaks will be obvious.
-
Transcriptome / RNA-seq. Table 1 shows 22.5Gb of transcriptomic reads but very little information is given about these data. How they were generated and filtered, and then how they were used during the annotation process needs more details.

Powered by Editorial Manager® and ProduXion Manager® from Aries Systems Corporation

Reply: Thank you very much for your reminding. The information has been added in the revised version (lines 106-124, line 229 in the revised ms).

Language. Overall the manuscript is well written, but there were many cases of grammatical errors and/or small typos, too many to catch them all in the minor comments below (I mostly stopped after the abstract). Thus, the manuscript would benefit from a proof-read to correct these small mistakes in English, it would not be a big task.
Reply: Thank you very much for your reminding. We corrected errors and typos thorough the manuscript in the revised version.

Finally, what is the criteria for "chromosome level assembly", a description that is used throughout the manuscript for their genome? I find it a bit puzzling that the final assembly has ~1000 scaffolds (~8000 contgs) and is described as chromosome level, but we are told there are 31 chromosomes for this species. By all accounts the authors have done a good job with such a large and repeat-rich genome, but to call it chromosome level is perhaps a bit misleading.
Reply: Thank you very much for your reminding. At last, based on the Hic technology, more than 99% of the total length were reliably anchored, ordered and orientated on the 31 chromosomes using Lachesis, and result in a scaffold N50 of 59.59 Mb of the assembly. This is comparable to the size of the chromosome, thus we call it chromosome level.

Minor comments
Line 28: "also called THE giant African snail…"
Reply: We have added the "the" in the revised ms.

Line 29 and elsewhere: the word "greedy" is a bit casual; suggest to use "extensive", "voracious" or other synonym
Reply: We have changed it into "voracious".

Line 30: "reproductIVE capacity"
Reply: We have corrected the mistake.

Line 30: "caused A world-wide…"
Reply: We have added the "a" in the revised version.

Line 32: "a pest THAT IS ABLE TO damage the agricultural crops"
Reply: We have corrected it according to your instructions.

Line 33: "many parasites THAT CAN threaten"
Reply: We have corrected it according to your instructions.

Line 34: "hindering the genetic"
Reply: We have deleted the "the" in the revised ms.

Line 37: "genome size TO BE 2.12 Gb"
Reply: We have changed it into "to be" in the revised ms.

Line 52: sentence has numerous grammatical errors, please rewrite.
Reply: We have rewritten it in the revised ms.

Line 66: "direct or indirect" – which is it?
Reply: We have changed it into "direct and indirect", which means both.

Line 71: the link provided is in Chinese and is difficult to navigate to the aforementioned list of invasive species
Reply: We apologize for the inconvenience, however, there is no English version for the list and we have marked the link as "in Chinese".

Line 75: mention what kind of animal Angiostrongylcantonensis is, e.g. "In addition, A. fulica is also the intermediate host of THE PARASITIC NEMATODE Angiostrongylcantonensis"
Reply: We have changed it in the revised ms.

Line 83: "…considered to be one of the most serious threat and a destructive terrestrial gastropod…"
Reply: We have deleted it in the revised ms.

Line 87: "molecular mechanismS UNDERLYINGinvestigations for its broad environmental adaptability"
Reply: We have corrected it in the revised ms.

Line 93: why these tissues specifically?
Reply: These tissues were used for DNA extraction and subsequent high-throughput sequencing, they were selected since these tissues were not easy to be contaminated by exogenous DNA from other species and the relatively high quantity of DNA.

Line 123: how does this estimate of heterozygosity (0.47%) compare to other mollusks?
Reply: High rate of heterozygosity (>1%) have been reported in bivalve genomes, and a previous genome study of Pomacea canaliculata revealed a high heterozygosity of 1%-2%. Thus a heterozygosity of 0.47% may be much lower than other molluscs.

Line 127: "provided additional supporting data for the statically STATISTICAL analysis"
Reply: We have corrected it in the revised ms.

Line 127: what statistical analysis is being referred to here?
Reply: It means the statistical analysis mentioned in the previous sentence, the correlation between repeat content and genome size.

Line 153: "pairsthat WITH both ends uniquely mapped"
Reply: We have corrected it in the revised ms.

Line 155: "StartNearRsite", "ExtremeFragments" etc – the detail is good but some of these parameters could be explained to tell readers what filtering was performed and why
Reply: These are parameters regarding invalid read pairs defined by hiclib and can be filtered with default settings. Actually these parameters have been used extensively (https://doi.org/10.1093/molbev/msw108, https://doi.org/10.1093/gigascience/giy120). The details are as follows:
ExtremeFragments: removes fragments with most and/or least # counts (the top 0.005 and bottom 0 were removed)
-StartNearRsite:Removes reads that start within x bp near rsite (5 bp near the rsite)
-LargeSmallFragments: removes very large and small fragments (100bp- 100000bp were retained)

Line 159: "had" -> "has"
Reply: We have corrected it in the revised ms.

Line 169: how many scaffolds? From Table 2 there are ~1000, which is way more that 31 expected number of chromosomes, so I suppose "chromosomal level" is a bit misleading? "near chromosomal level" might be more accurate
Reply: We have corrected it according to your instructions in the revised ms.

Line 186: which BUSCO gene set was used here?
Reply: We used the metazoa_odb9, and it has been added in the revised ms (line 210).

Line 188: so ~15% of detected BUSCO genes were found in multiple copy; is this a reflection of unresolved heterozygosity, or genuine gene duplications / paralogs? If the former, what has been done to remove these uncollapsed regions from the assembly? For example, their inclusion might upwardly bias the total assembly size or number of genes
Reply: Thank you very much for your reminding. The possibility can not be ruled out. However, as mentioned above, the sequencing depth shows that almost all regions of the assembly are homozygous, together with the fact that we used metazoa_odb9 as reference, we suspect that the detected multiple copy should be genuine gene

Powered by Editorial Manager® and ProduXion Manager® from Aries Systems Corporation

duplications / paralogs because of lineage-specific duplication. Moreover, a number of published genomes like Sillago sinica, Protosalanx hyalocranius, etc, detected multiple copy of BUSCO genes, which should be lineage-specific duplications, too.

Line 192: "From the NGS reads alignment, we detected 128,998 homologous SNP loci using the GATK pipeline, demonstrating the high base-level accuracy of 99.33%." I don't understand this statement: how does variant calling demonstrate a high base-level accuracy? What exactly does the 99.33% pertain to? How is "base-level" accuracy defined?
Reply: Thank you very for your reminding. The "homologous" should be "homozygous" and we are very sorry for the mistake. Generally, homozygous SNP means assembly error and heterozygous SNP means the assembly maybe right, and it has been used in many genome projects like Sillago sinica, Glyptosternon maculatum, etc, although the theory is not too serious. To avoid the confusion, we have deleted the sequence in the revised ms.

Line 197: RepeatModeler
Reply: We have corrected it in the revised ms.
Line 200: "All repetitive elements were masked in the genome for the BEFORE protein-coding gene prediction"
Reply: We have corrected it in the revised ms.

Line 206: "Full-length transcripts WERE obtained using Iso-Seq were mapped to the genome using Genewise" Also this sentence is slightly confusing – is Iso-Seq a tool that has generated 'transcripts' from the TBLASTN results in the previous sentence? I did not see any mention of RNA-seq data in the text, but there is some mentioned in Table 2. Please explain in more detail.
Reply: Iso-Seq is a technology and its full name is "isoform-sequencing", which can generate "full-length" isoforms of the transcripts from the same gene locus, and the details have been added in the revised ms. (lines 106-124 in the revised ms)

Line 221: Drosophila melanogaster is not a mollusc…
Reply: Drosophila melanogaster is used as an outgroup here and we corrected the mistake in the revised ms (lines 245-246 in the revised ms).

Line 223: "Only proteins from the longest transcript were usedfroFOR genes with alternative splices ISOFORMS"
Reply: We have corrected it in the revised ms.

Line 234: is this phylogenetic relationship unexpected?
Reply: The relationship (Aplysia_californica,(Achatina_fulica,Biomphalaria_glabrata)) is supported by a paper published in THE NAUTILUS (Title:On the phylogenetic relationships of the genus Mexistrophia and of the family Cerionidae (Gastropoda: Eupulmonata),
https://repository.si.edu/bitstream/handle/10088/27780/Harasewych%20et%20al.%202015.pdf?sequence=1&isAllowed=y), and the relationship between other species is in accord with a paper published in Gigascience (Title: The genome of the golden apple snail Pomacea canaliculata provides insight into stress tolerance and invasive adaptation, https://doi.org/10.1093/gigascience/giy101).

Line 243: "We annotatedPREDICTED 23,726 protein-coding genes in the A. fulica genome and 22,858 of genes were annotated WITH PUTATIVE FUNCTIONS." Functions based on sequence similarity, BLAST etc are of course putative
Reply: We have corrected it in the revised ms.

Table 2: what do the asterisks** represent?
Reply: It means the ultimate contigs since they were probably changed during the Hic step. We have added the statement in the revised ms.
Figure 1: "Figure 1. A picture of A. fulicathat INDIVIDUAL used for genome sequencing and assembly"
Reply: We have corrected it in the revised ms.

Figure 2: I struggle to extract anything useful from this figure, but I am not familiar with Hi-C data so maybe it's just me

Powered by Editorial Manager® and ProduXion Manager® from Aries Systems Corporation

Reply: The assumption of Hic is that the crosslinking signals are more strong as the loci located in a chromosome are more closer. Thus ideally the contact matrix should be around the diagonal line, just as is shown in the figure (figure3 in the revise ms).

Figure 3: Again, I'm not convinced this figure is very informative, as it currently is. For example, the majority of (unlabelled) points all overlap somewhere near the X-Y intercept, with only three outwith this cluster. Then the size of the points and their colour appear to convey the same information – why twice? I think the point of the figure is to demonstrate the high contiguity of A. fulica genome compared to other mollusc genomes, but does plotting scaffold N50 versus contig N50 really achieve this? Better would be to plot cumulative assembly span curves, i.e. number of scaffolds on X vs cumulative span on Y
Reply: Thank you very much for your suggestions. We have deleted the figure and listed these parameters such as scaffold N50 and contig N50 in Table 3 for comparison in the revised ms.

Figure 4: It is interesting that exon length is so conserved, but intron lengths are much more variable. Is there any evidence that intron lengths are bimodally distributed?
Reply: Bimodal distribution of the intron lengths was rarely reported. It is not surprise that the intron lengths is more variable than exon since the latter one is much more conservative than the former.

Reviewer #3: I thank the authors for the work presented on the manuscript "A chromosomal-level genome assembly for the giant African snail Achatina fulica". It is a great contribution for future studies of mollusk genomics and for the study of the molecular basis of invasiveness. I just have a few recommendations and comments.

1-) I would like to see the kmer distribution plot presented on the manuscript. It helps future researchers to understand the composition of this mollusk genome, and to plan future projects.
Reply: Thank you very much for your suggestions. In the revised ms, we have added the kmer spectra as Figure 2.

2-) On lines 133-137: Canu and Falcon are both good assemblers generating high quality data. After deciding to move forward with the Falcon assembly, I would like to know why the authors have decided not to run FALCON-Unzip on the assembly? The phasing of haplotypes has been shown to help avoid assembly errors in genomic areas of complex structural variation between haplotypes. Even though the further analysis (mapping quality, etc) show the assembled genome to be in good shape, it would be a good standard practice to run Falcon-Unzip before HiC scaffolding.
Reply: Thank you very much for your suggestions and we strongly agree with you. We believe that using Falcon-Unzip will generate a high-quality genome, especially the heterozygosity of the species is very high (>1% for example). However, we used FALCON here by considering that the heterozygosity of the species is not very high (0.47%).

3-) After Lanchesis, around 1000 contigs were not placed into chromosomes. Have you investigated the composition of such contigs? Can you present also the size distribution of them?
Reply: Thank you very much for your suggestions. We found that the average gene length is much shorter for contigs unanchored to chromosomes than the anchored ones (67.6 bp/kb vs 341.5 bp/kb), whereas the average length of repeat length is just the reverse. Out of the 1467 unanchored contigs, a total of 210 are longer than 10kb, with the longest one is 6,839 kb. And the size distribution of the unanchored contigs short than 10 kb is as follows:

4-) The sequencing of the transcriptome with IsoSeq technology was only briefly mentioned. Could you describe the evaluation of such transcripts in a few lines? For example, was it possible to find full-length transcripts sequenced?
Reply: Thank you very much for your suggestions. In this study, a number of 553,889 Full-length Non-chimeric sequences (FLNC) representing 23,726 gene loci were obtained. However, the 5' end of the mRNA might be degraded before sequencing and

Powered by Editorial Manager® and ProduXion Manager® from Aries Systems Corporation

	we could not detect it as we did for the 3' end since a polyA tail is a sign of completeness for the latter one. To evaluate the completeness of the isoforms, we compared them to the predicted mRNAs from genome sequences and found that 70.37% of the multi-exon FLNCs were really full-length sequences. ((lines 106-124 in the revised ms))<br><br>5-) Finally, just a last read to review the English would be advised. Two examples of misspelling: The tittle on line 409. And 'fro' on line 223.<br>Reply: Thank you very much for your reminding. We hope that all mistakes have been corrected in the revised version.
**Additional Information:**	
**Question**	**Response**
Are you submitting this manuscript to a special series or article collection?	No
**Experimental design and statistics**<br><br>Full details of the experimental design and statistical methods used should be given in the Methods section, as detailed in our Minimum Standards Reporting Checklist. Information essential to interpreting the data presented should be made available in the figure legends.<br><br>Have you included all the information requested in your manuscript?	Yes
**Resources**<br><br>A description of all resources used, including antibodies, cell lines, animals and software tools, with enough information to allow them to be uniquely identified, should be included in the Methods section. Authors are strongly encouraged to cite Research Resource Identifiers (RRIDs) for antibodies, model organisms and tools, where possible.<br><br>Have you included the information requested as detailed in our Minimum Standards Reporting Checklist?	Yes
**Availability of data and materials**<br><br>All datasets and code on which the	Yes

conclusions of the paper rely must be either included in your submission or deposited in publicly available repositories (where available and ethically appropriate), referencing such data using a unique identifier in the references and in the "Availability of Data and Materials" section of your manuscript.<br><br>Have you have met the above requirement as detailed in our Minimum Standards Reporting Checklist?	

Powered by Editorial Manager® and ProduXion Manager® from Aries Systems Corporation

Manuscript
Click here to access/download;Manuscript;manuscript_r1-1.doc
Click here to view linked References

A chromosomal-level genome assembly for the giant African snail *Achatina fulica*

Guo Yunhai[1, #], Zhang Yi[1, #], Liu Qin[1], Huang Yun[1], Mao Guangyao[1], Yue Zhiyuan[1], Eniola M. Abe[1], Li Jian[2], Wu Zhongdao[3], Li Shizhu[1], Zhou Xiaonong[1], Hu Wei[1,2,*], Xiao Ning[1,*]

[1] Key Laboratory of Parasite and Vector Biology of MOH, WHO Cooperation Center for Tropical Diseases, Joint Research Laboratory of Genetics and Ecology on Parasite-host Interaction, Chinese Center for Disease Control and Prevention & Fudan University, National Institute of Parasitic Diseases, Chinese Center for Diseases Control and Prevention, Shanghai 200025, China

[2] State Key Laboratory of Genetic Engineering, Ministry of Education Key Laboratory of Contemporary Anthropology, Department of Microbiology and Microbial Engineering, School of Life Science, Fudan University, Shanghai 200433, China

[3] Department of Parasitology, Zhongshan School of Medicine, Sun Yat-sen University, Guangzhou 510080, China

Abstract

Background:

Achatina fulica (A. fulica), also called the giant African snail, is the largest species in the reported terrestrial mollusks. Due to its voracious appetite, wide environmental adaptability, high growth rate and reproductive capacity, the species caused a world-wide invasion, mainly in Southeast Asia, Japan, the western Pacific islands and China. *A. fulica* is a pest that is able to damage agricultural crops, as well as an intermediate host of many parasites that can threaten human health. However, genomic information of *A. fulica* is still limited, hindering genetic and genomic studies with the aim to invasion control and management of the species.

Finding:

Using *K*mer-based method, we estimated the *A. fulica* genome size to be 2.12 Gb with a high repeat content up to 71%. About 101.6 Gb genomic long-read data of *A. fulica* were generated from the PacBio sequencing platform and assembled to the first *A. fulica* genome of 1.85 Gb with a contig N50 length of 726 kb. Using contact information from the Hi-C sequencing data, we successfully anchored 99.32% contig sequences into 31 chromosomes, leading to the final contig and scaffold N50 length of 721 kb and 59.6 Mb, respectively. The continuity, completeness and accuracy were evaluated by genome comparison with other mollusk genomes, BUSCO assessment and genomic read mapping. 23,726 protein-coding genes were predicted from the assembled genome, among which 96.34% of the genes were functionally annotated. The phylogenetic analysis using whole-genome protein-coding genes revealed that *A. fulica* separated from the common ancestor with *Biomphalaria glabrata* around 182 million years ago.

Conclusion:

As our best knowledge, the *A. fulica* genome was the first terrestrial mollusk genome reported so far. The chromosome sequences of *A. fulica* will provide the research community a valuable resource for the population genetics and environmental adaptation studies for the species, and furthermore, for the chromosome level of evolution investigation within mollusks.

Key Words: Giant African snail, *Achatina fulica*, PacBio, Hi-C, chromosome assembly

Data description

Introduction

The giant African snail, *A. fulica*, is a Gastropod species (**Figure 1**). It is the largest terrestrial mollusks with voracious appetite, strong environmental adaptability, and high growth and reproduction rate[1-3]. Originating from East Africa, *A. fulica* gradually invaded Southeast Asia, Japan and the western Pacific islands in the last century[4-6] with the direct and indirect help from humans[7-9].In mainland China, the first *A. fulica* invasion event was reported in 1931[10]. At present, the snail's natural distribution in the wild has been found in Guangdong, Hainan, Guangxi, southern parts of Yunnan Province and Fujian Province, and a county of Guizhou Province[11]. *A. fulica* was included as the first 16 alien invasive species in China (http://www.mee.gov.cn/gkml/zj/wj/200910/t20091022_172155.htm, in Chinese) in 2003, and was also listed by International Union for Conservation of Nature (IUCN) as the 100 most threatening alien invasive species[12]. This snail has been recognized as an agricultural and garden pest that has caused significant damages in both tropical and subtropical regions[9, 12, 13]. In addition, *A. fulica* is also the intermediate host of the parasitic nematode *Angiostrongyl cantonensis*. Human infection with angiostrongyliasis, which occurs mainly through consumption of snails carrying *A. cantonensis* larvae, causes eosinophilic meningoencephalitis[4, 11, 14-18]. As a consequence, *A. fulica* is attracting more and more attention in fields of both agricultural crops protection and human disease control.

To date, a variety of mollusk genomes have been analyzed and published, including two freshwater gastropods snails *Pomacea canaliculata*[19] and *Biomphalaria glabrata*[20]. However, no genome has been reported for terrestrial mollusks. *A. fulica* is considered to be a destructive terrestrial gastropod which poses a significant hazard to agriculture, the environment, biodiversity and human health. A chromosome genome of *A. chatina* could provide crucial resources in the population

genetics and evolution studies based on genomic sequencing data aiming to discover the invasion and adaptation history of *A. chatina*. Meanwhile, the genome could also be used to probe gene expression during the important biological processes, such as gene expression patterns in various developmental stages and the interaction of *Angiostrongylus* and *A. chatina*. In this work, we applied Illumina, PacBio and Hi-C techniques to construct the chromosome of *A. fulica*. The genome is the first terrestrial mollusk genome, providing an important reference for the molecular mechanisms underlying its broad environmental adaptability and the development of control strategy of the world-wide invasion.

Sample and sequencing

An adult snail (**Figure 1**), which was collected in Pingxiang city, Guangxi Autonomous Region, was used for reference genome construction. The snail was dissected and abdominal foot (17.4 g) and liver pancreas (40.4 g) tissues were collected and quickly frozen in liquid nitrogen overnight before transferring to -80 °C for storage. DNA was extracted using the traditional phenol/chloroform extraction method and was quality checked using agarose gel electrophoresis, meeting the requirement for library construction for the Illumina X Ten (Illumina Inc., San Diego, CA, USA) and for the PacBio Sequel (Pacific Biosciences of California, Menlo Park, CA, USA) sequencing platforms.

RNA was extracted from the pallium, liver, foot, spleen, stomach, gut, heart using TRIZOL reagents. The RNA quality was checked using the Nanodrop ND-1000 spectrophotometer (LabTech, USA) and 2100 Bioanalyzer (Agilent Technologies, USA) with RNA integrity number of 8. The RNA from each samples were equally mixed for the RNA sequencing on PacBio Sequel platform. Firstly, mRNA molecules were reversely transcribed to cDNA using Clontech SMARTer cDNA synthesis kit. After cDNA amplification and purification, two SMRTbell libraries of 0-4 kb and 4-10 kb were generated using the size selection in BluePippin Size Selection System

(Pacific Biosciences of California, Menlo Park, CA, USA) and protocols suggested by manufacturer. The finale libraries were sequenced in the PacBio SEQUEL platform (Pacific Biosciences of California, Menlo Park, CA, USA), resulting 12,439,996 subreads totaling about 22.5 Gb PacBio long reads with average length longer than 1,801 bps. Subsequently, a total of 782,613 circular consensus sequences (CCS) were generated based on the subreads, and a number of 553,889 Full-length Non-chimeric sequences (FLNC) representing 23,726 gene loci were obtained, eventually. All aforementioned data processing were performed using SMRT Link v5.0 (www.pacb.com). Moreover, about 70.37% of the multi-exon FLNCs were really full-length sequences embracing all the exons of the gene locus predicted from the whole genome sequences.

Using the DNA molecules from abdominal foot, a library with the insertion length of 300 bp were constructed and sequenced for Illumina sequencing platform according to the manufacturer's protocol. About 202.23 Gb short reads were obtained from the Illumina X Ten sequencing technology (**Table 1**), which was used for the following genome survey analysis, and for final base-level genome sequence correction. Meanwhile, four 20 kb libraries were constructed for PacBio Sequel sequencing. Using 16 sequencing SMRT cells, 104.6 Gb long reads were generated (**Table 1**). The mean and N50 lengths of the polymerases for sequencing cells ranged from 6.4 kb to 10.4 kb and from 12.3 kb to 20.3 kb for cells, respectively. Those long genomic DNA reads were used for reference genome construction.

Genome features estimation from *K*mer method

With sequencing data from the Illumina platform, several genome characters could be evaluated for *A. fulica.* To ensure the quality of the analysis, ambiguous bases and low-quality reads were trimmed and filtered using the HTQC package (version 1.92.3)[21]. The following quality control were performed under the framework of

HTQC. First, the qualities of bases at two read ends were checked. Bases in sliding 5 bp windows were deleted if the average quality of the window was below phred quality score of 20. Second, reads were filtered if the average phred quality score were smaller than 20 or the read length was shorter than 75 bp. Third, the mate reads were also removed if the corresponding reads were filtered.

The quality-controlled reads were used for genome character estimation. We calculated the number of each 17-mer from the sequencing data using the jellyfish software (version 2.0)[22], and the distribution was analyzed with GCE software (version 3)[23] and was shown in Figure 2. We estimated the genome size of 2.12 Gb with the heterozygosity of 0.47% and repeat content of 71% in the genome. Previous studies revealed that repeat content varies in mollusks, and that repeat content is correlated with genome size[24]. The large genome size and high proportion of repeat contents of *A. fulica* provided additional supporting data for the statistical analysis. Moreover, 10,000 pairs of short reads were extracted randomly and were compared to the nt database and no obvious external contamination were found.

Genome assembly by third-generation long reads

After removing adaptor sequences in polymerases, 101.6 Gb subreads were generated for the following whole genome assembly. The average and N50 length of subreads reached 5.25 kb and 8.80 kb, respectively. To optimize the genome assembly using the PacBio sequencing data, we applied two packages in the assembly process, Canu v1.8 [25] and FALCON v0.2.2 [26]. Canu package was first applied for the assembly with the default parameters. As a result, a 1.93 Gb genome was constructed with 10,417 contigs and a contig N50 length of 662.40 kb. FALCON was also employed using the length_cutoff and pr_length_cutoff parameters of 10 kb and 8 kb, respectively. We obtained 1.85 Gb genome with 8,585 contigs, with a contig N50 of 726.63 kb. We adopted the FALCON assembly as the reference genome for *A. fulica* (**Table 2**). The genome sequences were subsequently polished by PacBio long

reads using arrow[27] and Illumina short reads by pilon[28] to correct base errors. The corrected genome was further applied for the following chromosome assembly construction using Hi-C data.

In situ Hi-C library construction and chromosome assembly using Hi-C data

Liver pancreas tissue of *A. fulica* was used for library construction for Hi-C analysis and the library was constructed using the identical method in previous studies[29]. Finally, the library was sequenced with 150 paired-end mode on the Illumina HiSeq X Ten platform (San Diego, CA, United States). From the Illumina sequencing platform, 1,313.87 million paired-end reads were obtained for the Hi-C library (**Table 1**). The reads were mapped to the above *A. fulica* genome with Bowtie2 [30], with two ends of paired reads being mapped to the genome separately. To increase the interactive Hi-C reads ratio, an iterative mapping strategy was performed as previous studies, and only read pairs with both ends uniquely mapped were used for the following analysis. From the alignment status of two ends, self-ligation, non-ligation and other sorts of invalid reads, including StartNearRsite, PCR amplification, random break, LargeSmallFragments and ExtremeFragments, were filtered out by Hi-Clib[31]. Through the recognition of restriction sites in sequences, contact counts among contigs were calculated and normalized.

According to previous karyotype analyses, *A. fulica* has 31 chromosomes[32]. By clustering the contigs using the contig contact frequency matrix, we were able to correct some minor errors in the FALCON assembly results. Contigs with errors were broken into shorter contigs. We obtained 8,701 contigs, slightly more than the 8,585 contigs in the FALCON assembly. We successfully clustered these contigs into 31 groups in Lachesis[33] using the agglomerative hierarchical clustering method (**Figure 3**). Lachesis was further applied to order and orient the clustered contigs according to the contact matrix. As a result, 7,106 contigs were reliably anchored,

ordered and orientated on chromosomes, accounting for 99.32% of the total genome bases. The first near chromosomal-level assembly of *A. fulica* was obtained with 8,211 contigs, a contig N50 of 721.0 kb and a scaffold N50 of 59.59 Mb (**Table 2** and **Table 3**).

Genome quality evaluation

We assessed the quality of genome of *A. fulica* after the assembly process. The quality evaluation was carried out in three aspects: continuity, completeness and the mapping rate of NGS data.

First of all, we compared the sequence number and contig N50 length of *A. fulica* with public genome of mollusks and found that our assembly has a high quality on contig and scaffold N50 among mollusk genomes. (**Table 3**) Traditional chromosomal genome assembly requires physical maps and genetic maps, which is enormously time- and labor-consuming. With Hi-C data analysis, we successfully assembled *A. fulica* genome into near chromosome-level with just one individual.

Second, the assembled genome was subjected to the BUSCO (version 3.0, metazoa_odb9)[34] to assess the completeness of the genome. About 91.7% of the BUSCO genes were identified in *A. fulica* genome, and more than 84.7% of the BUSCO genes were single-copy completed in our genome, illuminating a high level of completeness of the genome.

Third, NGS short reads were aligned to the genome using BWA package (version 0.7.17)[35], and about 98.7% of paired reads were aligned to the genome, of which 98.24% were reads paired aligned.

Repeat element and gene annotation

Tandem Repeat Finder4.09 (TRF)[36] was used for repetitive element identification in the *A. fulica* genome. A *de novo* method applying RepeatModeler was used to detect transposable elements (TEs). The resulted *de novo* data, combined with known

repeat library from Repbase[37], were used to identify TEs in the *A. fulica* genome by RepeatMasker4-0-8 [38] software. All repetitive elements were masked in the genome before protein-coding gene prediction.

Protein-coding genes in the *A. fulica* genome were annotated using the *de novo* program Augustus0.2.1 [39]. Protein sequences of the closely related species including *Aplysia californica*, *Biomphalaria glabrata*，*Crassostrea gigas*，*Lottia gigantea* and *Patinopecten yessoensis*, were downloaded from the Ensembl database, and aligned to the *A. fulica* genome with TBLASTN2.6.0. Full-length transcripts obtained using Iso-Seq were mapped to the genome using Genewise[40]. Finally, gene models predicted from all above methods were combined by MAKERv2.31.10 [41], resulting in 23,726 protein-coding genes. The gene number, gene length, CDS length, exon length and intron length distribution were all comparable with the related mollusks (**Figure 4**).

To functionally annotate protein-coding genes in the *A. fulica* genome, we searched all predicted gene sequences to NCBI non-redundant nucleotide (NT) and protein (NR), Swiss-Prot databases by BLASTN[42] and BLASTX[43] utility. Blast2GO[44] was also used to assign gene ontology (GO)[45] and Kyoto Encyclopedia of Genes and Genomes (KEGG)[46] pathways. A threshold of e-value of 1e-5 was used for all BLAST applications. Finally, 22,858 (96.34%) genes were functionally annotated (**Table 4**).

Phylogenetic analysis of *A. fulica* with other mollusks

OrthoMCLv1.2 [47] was used to cluster gene families. First, proteins from *A. fulica* and the closely related mollusks, including *Aplysia californica*, *Biomphalaria glabrata*, *Crassostrea gigas*, *Lingula anatina*, *Lottia gigantea*, *Patinopecten yessoensis*, *Octopus bimaculoides*, *Helobdella robusta*, *Pomacea canaliculata*, and the outgroup, *Drosophila melanogaster*, were all-to-all blasted by BLASTP[43] utility with an e-value threshold of 1e-5. Only proteins from the longest transcript were used for genes with

alternative isoforms. We identified 25,448 gene families for *A. fulica* and the related species, among them 675 single-copy orthologs families were detected.

Using single-copy orthologs, we could probe the phylogenetic relationships for the *A. fulica* and other mollusks. To this end, protein sequences of single-copy genes were aligned using CLUSTALX2.0 [48]. Guided by the protein multi-sequence alignment, the alignment of the coding DNA sequences (CDS) for those genes were generated and concatenated for the following analysis. The phylogenetic relationships were constructed using PhyML3.0 [49] using the concatenated nucleotide alignment with the JTT+G+F model. The MCMCtree program in PAML4 [49] was used to estimate the species divergent time scales for the mollusks using approximate likelihood method and calibrated according to the records downloaded from Timetree (www.timetree.org). We found that *A. fulica* was most closely related to *Biomphalaria glabrata*, and the two species diverged from their common ancestor about 179.17 million years ago (MYA) (**Figure 5**).

Conclusion

We reconstructed the first chromosome level assembly for *A. fulica* using an integrated strategy of PacBio, Illumina and Hi-C technologies. Using the long reads from PacBio Sequel platform and short reads from the Illumina X Ten platform, we successfully constructed contig assembly for *A. fulica*. Leveraging contact information among contigs from Hi-C technology, we further improved the assembly to the near chromosome-level quality (**Table 3** and **Figure 3**). We predicted 23,726 protein-coding genes in the *A. fulica* genome and 22,858 of genes were functionally annotated with putative functions. With 675 single-copy orthologs from *A. fulica* and other related mollusks, we constructed the phylogenetic relationship of these mollusks, and found that *A. fulica* might have diverged from its common ancestor of *Biomphalaria glabrata* around 177.1-187.1 MYA. Given the increasing interests in mollusk genomic evolution and the biological importance of *A. fulica* as an invasive animal, our genomic and

transcriptome data provide valuable genetic resource for the following functional genomics investigations for the research community.

Ethics Statement

This study was approved by the Animal Care and Use committee of National Institute of Parasitic Diseases, Chinese Center for Disease Control and Prevention. All participates consent the study under the 'Ethics, consent and permissions' heading. All participants consent to publish the work under the 'Consent to publish' heading.

Availability of supporting data

The Illumina, PacBio and Hi-C sequencing data are available from NCBI via the accession number of SRR8369706, SRR8369311 and SRR8371669, respectively. The Illumina transcriptome sequencing data were deposited to NCBI via the accession number of SRR8371872 and SRR8371873. The genome, annotation and intermediate files were uploaded to GigaScience FTP server.

Competing interests

The authors declare that they have no competing interests.

Acknowledgement

This work was supported by the National Key Research and Development Program of China (No. 2016YFC1200500, 2016YFC1200502, 2016YFC1200503 and 2016YFC1202000). The authors thank Frasergen Bioinformatics for providing technique supports for this work.

Author Contributions

Z.X, H.W and X.N conceived the project. G.Y, Z.Y, L.Q collected the samples and extracted the genomic DNA. G.Y, Z.Y and L.Q performed the genome assembly and data analysis. G.Y, Z.X, H.W and X.N wrote the paper.

References

1. Schreurs J. *Investigations on the biology, ecology and control of Giant African Snail 290 in West New Guinea*. 1963. Manokwari Agricultural Research Station.
2. Albuquerque FS, Peso-Aguiar MC and Assunção-Albuquerque MJ. Distribution,feeding behavior and control strategies of the exotic land snail Achatinafulica (Gastropoda:Pulmonata) in the Northeast of Brazil. BrazJBiol. 2008;68:6.
3. Thiengo SC, Fernandez MA, Torres EJ, Coelho PM and Lanfredi RM. First record of anematode Metastrongyloidea (Aelurostrongylus abstrusus larvae) in Achatina (Lissachatina) fulica (Mollusca,Achatinidae) in Brazil. J Invertebr Pathol. 2008;98:6.
4. Lv S, Zhang Y and Liu HX. Invasive Snails and an Emerging Infectious Disease: Results from the First National Survey on Angiostrongylus cantonensis in China. BioOne. 2009; doi:10.1371/journal.pntd.0000368.
5. Cowie RH. *Non-indigenous land and freshwater molluscs in the islands of the Pacific: Conservation impacts and threats*. 2000.
6. Cowie RH. Can snails ever be effective and safe biocontrol agents? Int J Pest Manage. 2001;47:18.
7. Cowie RH and Robinson DG. Pathways of introduction of nonindigenous land and freshwater snails and slugs. Washington DC: Island Press; 2003.
8. Kotangale JP. Giant African snail (Achatina fulica Bowdich). 2011;J Environ Sci Eng 53:6.
9. Raut SK and Barker GM. Achatina fulica Bowdich and Other Achatinidae as Pests in Tropical Agriculture. UK: CABI International; 2002.
10. Jarreit VHC. The spread of the snail Achatina fulica to south China. Hong Kong Nat. 1931;2:3.
11. Shan L, Yi Z and Peter S. Emerging Angiostrongyliasis in Mainland China. Emerging Infectious Diseases. 2008;14 1:4.
12. Lowe S, Browne SM, Boudjrlas S and De Poorter M. 100 of the world's worst invasive alien species: A selection from the global invasive species database. The Invasive Species Specialists Group of the Species Survival Commission of the World Conservation Union. Auckland: Hollands Printing; 2000.
13. Mead AR. Pulmonates volume 2B. Economic malacology with particular reference to Achatina fulica. London: Academic Press; 1979.
14. Alicata JE. The discovery of Angiostrongylus cantonensis as a cause of human eosinophilic meningitis. Parasitol Today. 1991;7 6:151-3.
15. Prociv P, Spratt DM and Carlisle MS. Neuro-angiostrongyliasis: unresolved issues. Int J Parasitol. 2000;30 12-13:1295-303.
16. Deng ZH, Zhang QM, Huang SY and Jones JL. First provincial survey of Angiostrongylus cantonensis in Guangdong Province, China. Trop Med Int Health. 2012;17:4.
17. Maldonado JA, Simoes RO, Oliveira AP, Motta EM, Fernandez MA, Pereira ZM, et al. First report of Angiostrongylus cantonensis (Nematoda: Metastrongylidae) in Achatina fulica (Mollusca: Gastropoda) from Southeast and South Brazil. Mem Inst Oswaldo Cruz. 2010;105:4.
18. Vitta A, Polseela R, Nateeworanart S and Tattiyapong M. Survey of Angiostrongylus cantonensis in rats and giant African land snails in Phitsanulok Province, Thailand. Asian Pac J Trop Med. 2011;4:3.
19. Liu C, Zhang Y, Ren Y, Wang H, Li S, Jiang F, et al. The genome of the golden apple snail

Pomacea canaliculata provides insight into stress tolerance and invasive adaptation. GigaScience. 2018;7 9 doi:10.1093/gigascience/giy101.

20. Adema CM, Hillier LW, Jones CS, Loker ES, Knight M, Minx P, et al. Whole genome analysis of a schistosomiasis-transmitting freshwater snail. Nature communications. 2017;8:15451. doi:10.1038/ncomms15451.
21. Neff KL, Argue DP, Ma AC, Lee HB, Clark KJ and Ekker SC. Mojo Hand, a TALEN design tool for genome editing applications. BMC Bioinformatics. 2013;14:1. doi:10.1186/1471-2105-14-1.
22. Marcais G and Kingsford C. A fast, lock-free approach for efficient parallel counting of occurrences of k-mers. Bioinformatics. 2011;27 6:764-70. doi:10.1093/bioinformatics/btr011.
23. Binghang Liu YS, Jianying Yuan,Xuesong Hu,Hao Zhang,Nan Li,Zhenyu Li,Yanxiang Chen,Desheng Mu,Wei Fan. Estimation of genomic characteristics by analyzing k-mer frequency in de novo genome projects. Quantitative Biology. 2013;35:62-7.
24. Murgarella M, Puiu D, Novoa B, Figueras A, Posada D and Canchaya C. A First Insight into the Genome of the Filter-Feeder Mussel Mytilus galloprovincialis. PloS one. 2016;11 3:e0151561. doi:10.1371/journal.pone.0151561.
25. Koren S, Walenz BP, Berlin K, Miller JR, Bergman NH and Phillippy AM. Canu: scalable and accurate long-read assembly via adaptive k-mer weighting and repeat separation. Genome Res. 2017;27 5:722-36. doi:10.1101/gr.215087.116.
26. Chin CS, Peluso P, Sedlazeck FJ, Nattestad M, Concepcion GT, Clum A, et al. Phased diploid genome assembly with single-molecule real-time sequencing. Nat Methods. 2016;13 12:1050-4. doi:10.1038/nmeth.4035.
27. Chin C-S, Alexander DH, Marks P, Klammer AA, Drake J, Heiner C, et al. Nonhybrid, finished microbial genome assemblies from long-read SMRT sequencing data. Nature methods. 2013;10 6:563.
28. Walker BJ, Abeel T, Shea T, Priest M, Abouelliel A, Sakthikumar S, et al. Pilon: an integrated tool for comprehensive microbial variant detection and genome assembly improvement. PloS one. 2014;9 11:e112963.
29. Gong G, Dan C, Xiao S, Guo W, Huang P, Xiong Y, et al. Chromosomal-level assembly of yellow catfish genome using third-generation DNA sequencing and Hi-C analysis. Gigascience. 2018; doi:10.1093/gigascience/giy120.
30. Langmead B, Trapnell C, Pop M and Salzberg SL. Ultrafast and memory-efficient alignment of short DNA sequences to the human genome. Genome Biol. 2009;10 3:R25. doi:10.1186/gb-2009-10-3-r25.
31. Burton JN, Adey A, Patwardhan RP, Qiu R, Kitzman JO and Shendure J. Chromosome-scale scaffolding of de novo genome assemblies based on chromatin interactions. Nature biotechnology. 2013;31 12:1119.
32. Sun T. Chromosomal studies in three land snails. Sinozoologia. 1995;12:154-62.
33. Near TJ, Dornburg A, Eytan RI, Keck BP, Smith WL, Kuhn KL, et al. Phylogeny and tempo of diversification in the superradiation of spiny-rayed fishes. Proceedings of the National Academy of Sciences of the United States of America. 2013;110 31:12738.
34. Simão FA, Waterhouse RM, Ioannidis P, Kriventseva EV and Zdobnov EM. BUSCO: assessing genome assembly and annotation completeness with single-copy orthologs. Bioinformatics. 2015;31 19:3210-2.

35. Li H and Durbin R. Fast and accurate short read alignment with Burrows–Wheeler transform. bioinformatics. 2009;25 14:1754-60.
36. Benson G. Tandem repeats finder: a program to analyze DNA sequences. Nucleic Acids Res. 1999;27 2:573-80.
37. Bao W, Kojima KK and Kohany O. Repbase Update, a database of repetitive elements in eukaryotic genomes. Mob DNA. 2015;6:11. doi:10.1186/s13100-015-0041-9.
38. Chen N. Using RepeatMasker to identify repetitive elements in genomic sequences. Current protocols in bioinformatics. 2004;5 1:4.10. 1-4.. 4.
39. Stanke M, Keller O, Gunduz I, Hayes A, Waack S and Morgenstern B. AUGUSTUS: ab initio prediction of alternative transcripts. Nucleic acids research. 2006;34 suppl_2:W435-W9.
40. Birney E, Clamp M and Durbin R. GeneWise and genomewise. Genome research. 2004;14 5:988-95.
41. Cantarel BL, Korf I, Robb SM, Parra G, Ross E, Moore B, et al. MAKER: an easy-to-use annotation pipeline designed for emerging model organism genomes. Genome research. 2008;18 1:188-96.
42. Gertz EM, Yu YK, Agarwala R, Schaffer AA and Altschul SF. Composition-based statistics and translated nucleotide searches: improving the TBLASTN module of BLAST. BMC Biol. 2006;4:41. doi:10.1186/1741-7007-4-41.
43. Camacho C, Coulouris G, Avagyan V, Ma N, Papadopoulos J, Bealer K, et al. BLAST+: architecture and applications. BMC Bioinformatics. 2009;10:421. doi:10.1186/1471-2105-10-421.
44. Conesa A, Götz S, García-Gómez JM, Terol J, Talón M and Robles M. Blast2GO: a universal tool for annotation, visualization and analysis in functional genomics research. Bioinformatics. 2005;21 18:3674-6.
45. Consortium GO. The Gene Ontology (GO) database and informatics resource. Nucleic acids research. 2004;32 suppl_1:D258-D61.
46. Kanehisa M and Goto S. KEGG: kyoto encyclopedia of genes and genomes. Nucleic acids research. 2000;28 1:27-30.
47. Li L, Stoeckert CJ and Roos DS. OrthoMCL: identification of ortholog groups for eukaryotic genomes. Genome research. 2003;13 9:2178-89.
48. Thompson JD, Gibson TJ and Higgins DG. Multiple sequence alignment using ClustalW and ClustalX. Current protocols in bioinformatics. 2003; 1:2.3. 1-2.3. 22.
49. Guindon S, Lethiec F, Duroux P and Gascuel O. PHYML Online—a web server for fast maximum likelihood-based phylogenetic inference. Nucleic acids research. 2005;33 suppl_2:W557-W9.
50. Zhang G, Fang X, Guo X, Li L, Luo R, Xu F, et al. The oyster genome reveals stress adaptation and complexity of shell formation. Nature. 2012;490 7418:49-54. doi:10.1038/nature11413.
51. Takeuchi T, Kawashima T, Koyanagi R, Gyoja F, Tanaka M, Ikuta T, et al. Draft genome of the pearl oyster Pinctada fucata: a platform for understanding bivalve biology. DNA research : an international journal for rapid publication of reports on genes and genomes. 2012;19 2:117-30. doi:10.1093/dnares/dss005.
52. Takeuchi T, Koyanagi R, Gyoja F, Kanda M, Hisata K, Fujie M, et al. Bivalve-specific gene expansion in the pearl oyster genome: implications of adaptation to a sessile lifestyle. Zoological letters. 2016;2:3. doi:10.1186/s40851-016-0039-2.

53. Du X, Fan G, Jiao Y, Zhang H, Guo X, Huang R, et al. The pearl oyster Pinctada fucata martensii genome and multi-omic analyses provide insights into biomineralization. GigaScience. 2017;6 8:1-12. doi:10.1093/gigascience/gix059.
54. Mun S, Kim YJ, Markkandan K, Shin W, Oh S, Woo J, et al. The Whole-Genome and Transcriptome of the Manila Clam (Ruditapes philippinarum). Genome biology and evolution. 2017;9 6:1487-98. doi:10.1093/gbe/evx096.
55. Wang S, Zhang J, Jiao W, Li J, Xun X, Sun Y, et al. Scallop genome provides insights into evolution of bilaterian karyotype and development. Nature ecology & evolution. 2017;1 5:120. doi:10.1038/s41559-017-0120.
56. Schell T, Feldmeyer B, Schmidt H, Greshake B, Tills O, Truebano M, et al. An annotated draft genome for Radix auricularia (Gastropoda, Mollusca). Genome biology and evolution. 2017; doi:10.1093/gbe/evx032.
57. Albertin CB, Simakov O, Mitros T, Wang ZY, Pungor JR, Edsinger-Gonzales E, et al. The octopus genome and the evolution of cephalopod neural and morphological novelties. Nature. 2015;524 7564:220-4. doi:10.1038/nature14668.
58. Simakov O, Marletaz F, Cho SJ, Edsinger-Gonzales E, Havlak P, Hellsten U, et al. Insights into bilaterian evolution from three spiralian genomes. Nature. 2013;493 7433:526-31. doi:10.1038/nature11696.
59. Kenny NJ, Namigai EK, Marletaz F, Hui JH and Shimeld SM. Draft genome assemblies and predicted microRNA complements of the intertidal lophotrochozoans Patella vulgata (Mollusca, Patellogastropoda) and Spirobranchus (Pomatoceros) lamarcki (Annelida, Serpulida). Marine genomics. 2015;24 Pt 2:139-46. doi:10.1016/j.margen.2015.07.004.
60. Barghi N, Concepcion GP, Olivera BM and Lluisma AO. Structural features of conopeptide genes inferred from partial sequences of the Conus tribblei genome. Molecular genetics and genomics : MGG. 2016;291 1:411-22. doi:10.1007/s00438-015-1119-2.
61. Uliano-Silva M, Dondero F, Dan Otto T, Costa I, Lima NCB, Americo JA, et al. A hybrid-hierarchical genome assembly strategy to sequence the invasive golden mussel, Limnoperna fortunei. GigaScience. 2018;7 2 doi:10.1093/gigascience/gix128.
62. Sun J, Zhang Y, Xu T, Zhang Y, Mu H, Zhang Y, et al. Adaptation to deep-sea chemosynthetic environments as revealed by mussel genomes. Nature ecology & evolution. 2017;1 5:121. doi:10.1038/s41559-017-0121.
63. Jiao W, Fu X, Dou J, Li H, Su H, Mao J, et al. High-resolution linkage and quantitative trait locus mapping aided by genome survey sequencing: building up an integrative genomic framework for a bivalve mollusc. DNA research : an international journal for rapid publication of reports on genes and genomes. 2014;21 1:85-101. doi:10.1093/dnares/dst043.
64. Luo YJ, Takeuchi T, Koyanagi R, Yamada L, Kanda M, Khalturina M, et al. The Lingula genome provides insights into brachiopod evolution and the origin of phosphate biomineralization. Nature communications. 2015;6:8301. doi:10.1038/ncomms9301.
65. Li C, Liu X, Liu B, Ma B, Liu F, Liu G, et al. Draft genome of the Peruvian scallop Argopecten purpuratus. GigaScience. 2018;7 4 doi:10.1093/gigascience/giy031.

Tables and Figures

Table 1: Sequencing data generated for *A.fulica* genome assembly and annotation

Library type	Platform	Library size (bp)	Data size (Gb)	Application
Short reads	HiSeq X Ten	350	202.24	Genome survey and genomic base correction
Long reads	PacBio SEQUEL	20,000	101.63	Genome assembly
Hi-C	HiSeq X Ten	300-500	199.73	Chromosome construction

Table 2: Statistics for genome assembly of *A. fulica*

Sample ID	Length		Number	
	Contig** (bp)	Scaffold (bp)	Contig**	Scaffold
Total	1,852,282,574	1,855,883,074	8,211	1,010
Max	5,947,392	116,558,012	-	-
N50	721,038	59,589,303	697	13
N60	538,883	58,013,356	995	16
N70	399,612	53,672,006	1,396	20
N80	268,901	50,673,968	1,957	23
N90	141,756	44,109,545	2,888	27

The two stars (**) means the ultimate contigs since they were probably modified during the Hic step.

Table 3 Summary of the genome of *A. fulica* and other published mollusk genomes.

Species	Size* (Mb)	Contig N50 (kb)	Scaffold N50 (kb)
Achatina fulica (this study)**	2,120	721	59,590
Pomacea canaliculata[19]**	570	995	38,000
Crassostrea gigas[50]	545	7.5	401
Pinctada fucata[51]	1,150	1.6	14.5
Pinctada fucata new[52]	1,150	21	324
Pinctada fucata V2[53]	1,150	21	167
Biomphalaria glabrata[20]	931	7.3	48
Ruditapes philippinarum[54]	1,370	3.3	32.7
Patinopecten yessoensis[55]**	1,430	38	41,000
Radix auricularia[56]	1,600	0.324	578
Octopus bimaculoides[57]	2,800	5.4	470
Mytilus galloprovincialis[24]	1,600	2.6	2.9
Lottia gigantea[58]	420	96	1,870
Patella vulgata[59]	1,460	3.1	3.1
Aplysia californica	1,760	9.6	917
Conus tribblei[60]	2,760	0.85	215
Limnoperna fortunei[61]	1,600	10	312
Bathymodiolus platifrons[62]	1,640	13.2	343
Modiolus philippinarum[62]	2,380	19.7	100.2
Chlamys farreri[63]	1,200	1.2	1.5
Lingula anatina[64]	463	55	294
Argopecten prupruatus[65]	885	80.1	1,020

* Estimated the genome size

** Genomes assembled into near chromosomal level

Table 4: Statistics for genome annotation of *A. fulica*

Database	Number	Percent
InterPro	16,252	68.50
GO	12,101	51.00
KEGG ALL	21,325	89.88
KEGG KO	10,161	42.83
Swissprot	17,050	71.86
TrEMBL	22,403	94.42
NR	22,553	95.06
Total	23,726	

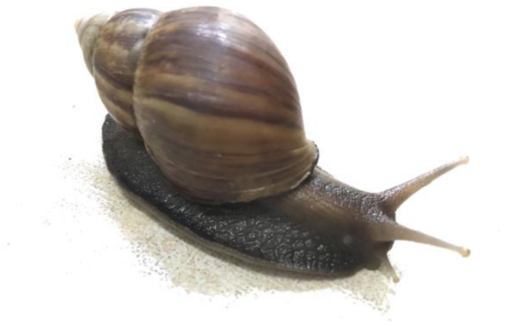

Figure 1. *A. fulica* individual **used for genome sequencing and assembly.**

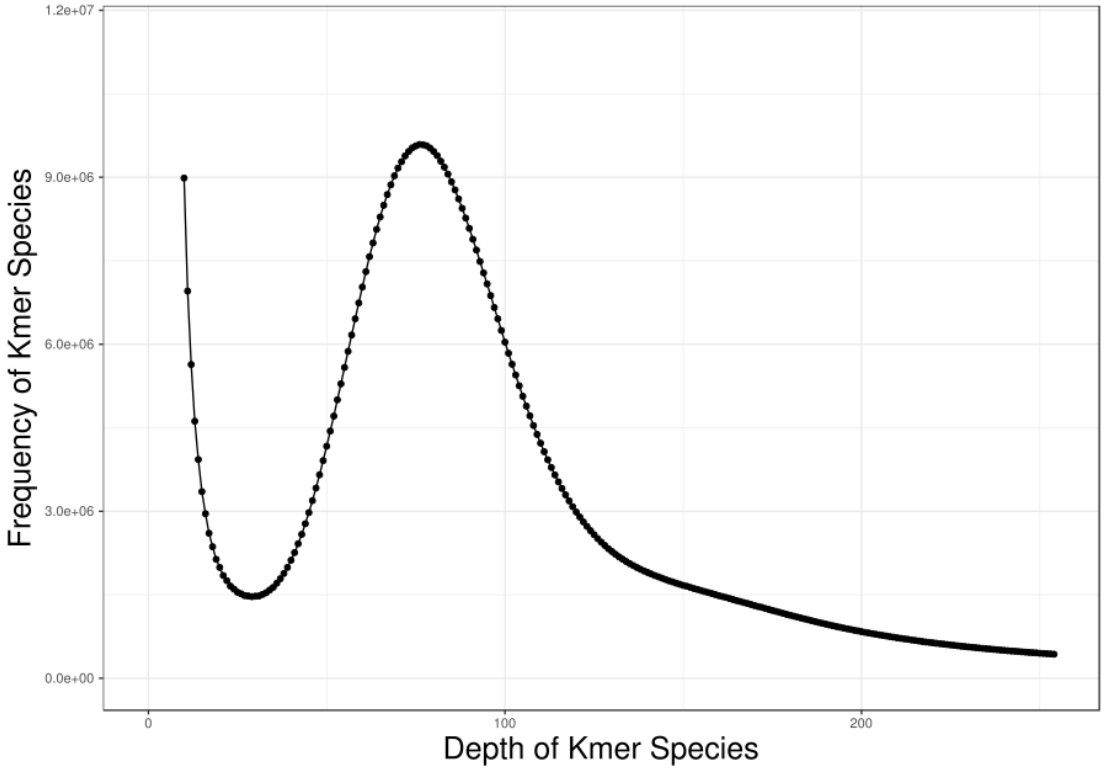

Figure 2. The distribution of *K*mer species estimated for *A. fulica*. The total number of *K*mer species is 178,847,565,204, with the peak value (depth) is 76.

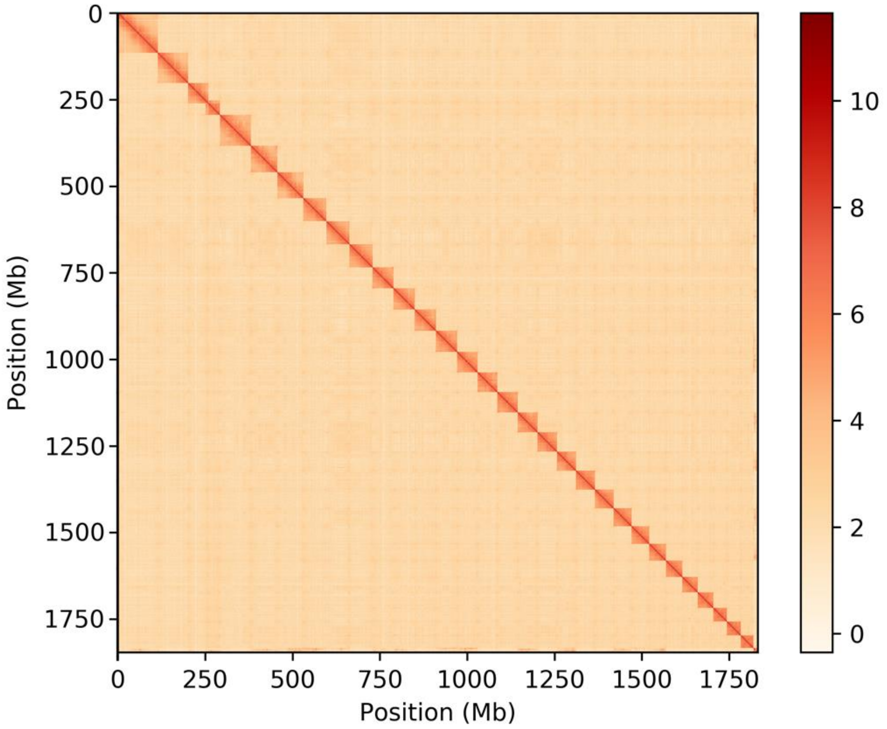

Figure 3. Contact matrix generated from the Hi-C data analysis showing sequence interactions in chromosomes. The logarithm of the contact density was showed in the color bar.

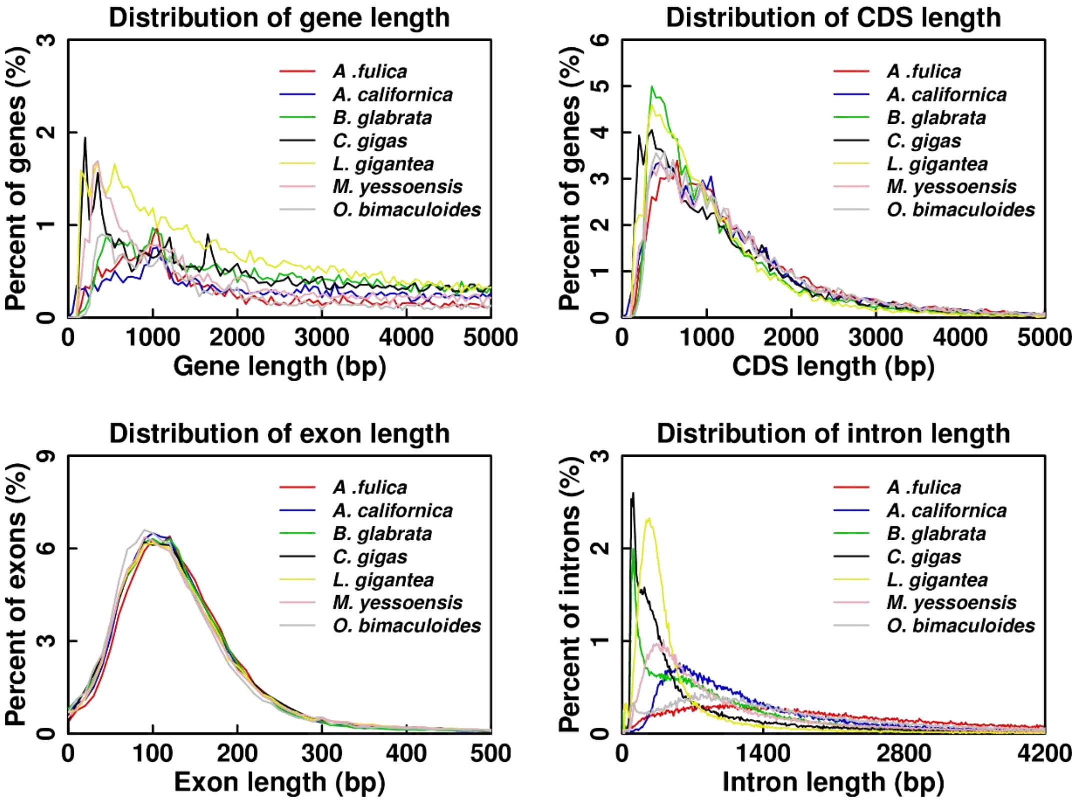

Figure 4. Length distribution comparison on total gene, CDS, exon, and intron of annotated gene models of *A. fulica* with other closely related insect species. The comparison of length distribution of genes (A), CDS (B), exon (C) and intron (D) for *A. fulica* to those in *A. californica* , *B. glabrata* , *C. gigas* , *L. gigantea* , *P. yessoensis* and *O. bimaculoides*.

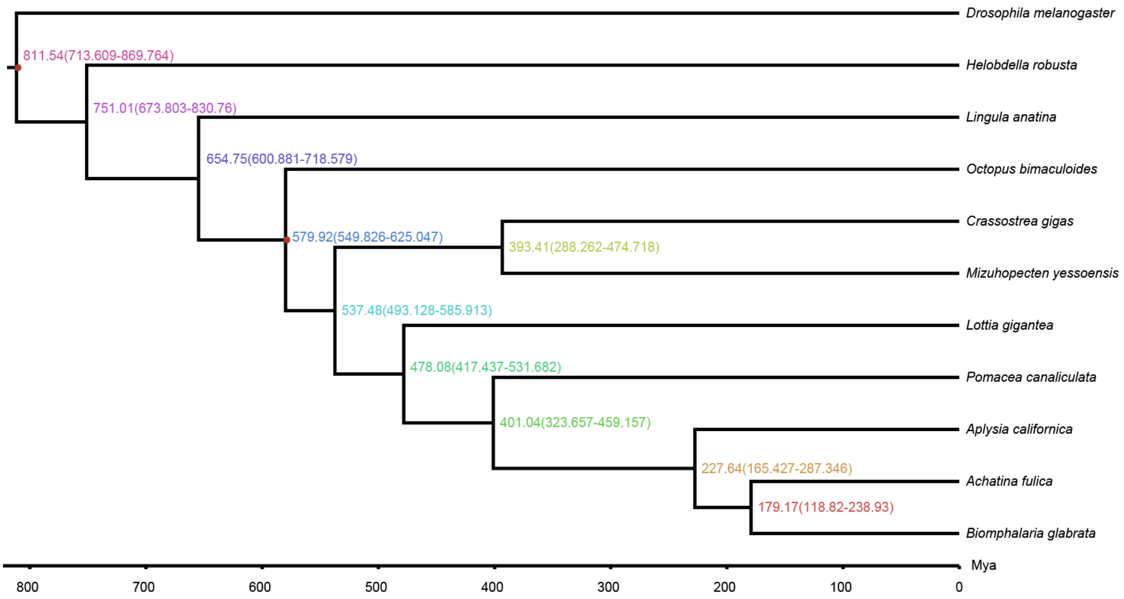

Figure 5. Phylogenetic relationship between A. fulica and related species. The divergence time (million years ago) and the 95% confidential intervals are labeled at branch sites and the red dots in the tree illuminated the speciation for the time recalibration.
