# Supplementary material for: A chromosomal-level genome assembly for the giant African snail Achatina fulica
Source: Gigascience. 2019 Oct 21;8(10):giz124. doi: 10.1093/gigascience/giz124 (PMC6802634; doi:10.1093/gigascience/giz124)
Supplement: giz124_GIGA-D-19-00006_Revision_3 [file giz124_giga-d-19-00006_revision_3.pdf]

GigaScience

A chromosomal-level genome assembly for the giant African snail Achatina fulica

--Manuscript Draft--

**Manuscript Number:**	GIGA-D-19-00006R3
**Full Title:**	A chromosomal-level genome assembly for the giant African snail Achatina fulica
**Article Type:**	Data Note
**Funding Information:**	This work was supported by the National Key Research and Development Program of China (No. 2016YFC1200500 and 2016YFC1202000) — Dr Ning Xiao
**Abstract:**	Background:<br>Achatina fulica (A. fulica), also called the giant African snail, is the largest terrestrial mollusk species. Due to its voracious appetite, wide environmental adaptability, high growth rate and reproductive capacity, it has become an invasive species across the world, mainly in Southeast Asia, Japan, the western Pacific islands and China. A. fulica is a pest that is able to damage agricultural crops, as well as an intermediate host of many parasites that can threaten human health. However, genomic information of A. fulica is still limited, hindering genetic and genomic studies for invasion control and management of the species.<br>Finding:<br>Using a Kmer-based method, we estimated the A. fulica genome size to be 2.12 Gb with a high repeat content up to 71%. Roughly 101.6 Gb genomic long-read data of A. fulica were generated from the PacBio sequencing platform and assembled to produce a first A. fulica genome of 1.85 Gb with a contig N50 length of 726 kb. Using contact information from the Hi-C sequencing data, we successfully anchored 99.32% contig sequences into 31 chromosomes, leading to the final contig and scaffold N50 length of 721 kb and 59.6 Mb, respectively. The continuity, completeness and accuracy were evaluated by genome comparison with other mollusk genomes, BUSCO assessment and genomic read mapping. 23,726 protein-coding genes were predicted from the assembled genome, among which 96.34% of the genes were functionally annotated. The phylogenetic analysis using whole-genome protein-coding genes revealed that A. fulica separated from a common ancestor with Biomphalaria glabrata around 182 million years ago.<br>Conclusion:<br>To the best of our knowledge, the A. fulica genome is the first terrestrial mollusk genome published to date. The chromosome sequence of A. fulica will provide the research community with a valuable resource for population genetics and environmental adaptation studies for the species as well as investigations of the chromosome level of evolution within mollusks.
**Corresponding Author:**	ning xiao<br><br>CHINA
**Corresponding Author Secondary Information:**	
**Corresponding Author's Institution:**	
**Corresponding Author's Secondary Institution:**	
**First Author:**	Yunhai Guo
**First Author Secondary Information:**	
**Order of Authors:**	Yunhai Guo<br>Yi Zhang<br>Qin Liu<br>Yun Huang

Powered by Editorial Manager® and ProduXion Manager® from Aries Systems Corporation

	Guangyao Mao
	Zhiyuan Yue
	Eniola M. Abe
	Jian Li
	Zhongdao Wu
	Shizhu Li
	Xiaonong Zhou
	Wei Hu
	Ning Xiao
**Order of Authors Secondary Information:**	
**Response to Reviewers:**	Reviewer #1: Regarding RNA samples, the authors commented that "we eventually selected high-quality samples for the sequencing." and showed Bioanalyzer report of only four samples in the supplementary. In the revised manuscript, however, they described that "RNA was extracted from the pallium, liver, foot, spleen, stomach, gut, heart...". Which four samples or tissues were actually used for RNA extraction and sequencing?<br>Reply: Thanks a lot for reminding. We have collected multi samples for pallium, liver, foot, spleen, stomach, gut and heart tissues. The qualities of RNA samples were summarized in the following table. We selected the high-quality samples highlighted by red for the PacBio library construction and sequencing in the Supplementary Table S1, covering all tissues that mentioned above. We have accordingly revised the manuscript and supplementary information.
**Additional Information:**	
**Question**	**Response**
Are you submitting this manuscript to a special series or article collection?	No
**Experimental design and statistics**<br><br>Full details of the experimental design and statistical methods used should be given in the Methods section, as detailed in our Minimum Standards Reporting Checklist. Information essential to interpreting the data presented should be made available in the figure legends.<br><br>Have you included all the information requested in your manuscript?	Yes
**Resources**<br><br>A description of all resources used, including antibodies, cell lines, animals and software tools, with enough information to allow them to be uniquely identified, should be included in the	Yes

Methods section. Authors are strongly encouraged to cite Research Resource Identifiers (RRIDs) for antibodies, model organisms and tools, where possible.<br><br>Have you included the information requested as detailed in our Minimum Standards Reporting Checklist?	
**Availability of data and materials**<br><br>All datasets and code on which the conclusions of the paper rely must be either included in your submission or deposited in publicly available repositories (where available and ethically appropriate), referencing such data using a unique identifier in the references and in the "Availability of Data and Materials" section of your manuscript.<br><br>Have you have met the above requirement as detailed in our Minimum Standards Reporting Checklist?	Yes

Powered by Editorial Manager® and ProduXion Manager® from Aries Systems Corporation

Manuscript
Click here to view linked References
Click here to access/download;Manuscript;006-manuscript_r3--HL.doc

A chromosomal-level genome assembly for the giant African snail *Achatina fulica*

Guo Yunhai[1,2,#], Zhang Yi[1,2,#], Liu Qin[1,2], Huang Yun[1,2], Mao Guangyao[1,2], Yue Zhiyuan[1,2], Eniola M. Abe[1,2], Li Jian[3], Wu Zhongdao[4], Li Shizhu[1,2], Zhou Xiaonong[1,2], Hu Wei[1,2,3,*], Xiao Ning[1,2,*]

[1]National Institute of Parasitic Diseases, Chinese Center for Disease Control and Prevention

[2]Key Laboratory of Parasite and Vector Biology, Ministry of Health, Shanghai, China

[3]Department of Microbiology and Microbial Engineering, School of Life Sciences, Fudan University, Shanghai 200438, China

[4]Department of Parasitology, Zhongshan School of Medicine, Sun Yat-sen University, Guangzhou 510080, China

ORCID:

Ning Xiao, 0000-0002-1361-7013

Eniola Abe: 0000-0002-9573-5506

Abstract

Background:

Achatina fulica (A. fulica), also called the giant African snail, is the largest terrestrial mollusk species. Due to its voracious appetite, wide environmental adaptability, high growth rate and reproductive capacity, it has become an invasive species across the world, mainly in Southeast Asia, Japan, the western Pacific islands and China. *A. fulica* is a pest that is able to damage agricultural crops, as well as an intermediate host of many parasites that can threaten human health. However, genomic information of *A. fulica* is still limited, hindering genetic and genomic studies for invasion control and management of the species.

Finding:

Using a *K*mer-based method, we estimated the *A. fulica* genome size to be 2.12 Gb with a high repeat content up to 71%. Roughly 101.6 Gb genomic long-read data of *A. fulica* were generated from the PacBio sequencing platform and assembled to produce a first *A. fulica* genome of 1.85 Gb with a contig N50 length of 726 kb. Using contact information from the Hi-C sequencing data, we successfully anchored 99.32% contig sequences into 31 chromosomes, leading to the final contig and scaffold N50 length of 721 kb and 59.6 Mb, respectively. The continuity, completeness and accuracy were evaluated by genome comparison with other mollusk genomes, BUSCO assessment and genomic read mapping. 23,726 protein-coding genes were predicted from the assembled genome, among which 96.34% of the genes were functionally annotated. The phylogenetic analysis using whole-genome protein-coding genes revealed that *A. fulica* separated from a common ancestor with *Biomphalaria glabrata* around 182 million years ago.

Conclusion:

To the best of our knowledge, the *A. fulica* genome is the first terrestrial mollusk genome published to date. The chromosome sequence of *A. fulica* will provide the research community with a valuable resource for population genetics and environmental adaptation studies for the species as well as investigations of the chromosome level of evolution within mollusks.

Key Words: Giant African snail, *Achatina fulica*, PacBio, Hi-C, chromosome assembly

Data description

Introduction

The giant African snail, *A. fulica* (NCBI: txid6530), is a Gastropod species (**Figure 1**). It is the largest terrestrial mollusk, with a voracious appetite, strong environmental adaptability, and high growth and reproduction rate[1-3]. Originating in East Africa, *A. fulica* over the last century it has gradually invaded Southeast Asia, Japan and the western Pacific islands [4-6] with the direct and indirect help from humans[7-9]. In mainland China, the first *A. fulica* invasion event was reported in 1931[10]. At present, the snail's natural distribution in the wild has been found in Guangdong, Hainan, Guangxi, southern parts of Yunnan Province and Fujian Province, and a county of Guizhou Province[11]. *A. fulica* was included as the first 16 alien invasive species in China (http://www.mee.gov.cn/gkml/zj/wj/200910/t20091022_172155.htm, in Chinese) in 2003, and was also listed by International Union for Conservation of Nature (IUCN) as among the 100 most threatening alien invasive species[12]. This snail has been recognized as an agricultural and garden pest causing significant damage in both tropical and subtropical regions[9, 12, 13]. In addition, *A. fulica* is also the intermediate host of the parasitic nematode *Angiostrongyl cantonensis*. Human infection with angiostrongyliasis, which occurs mainly through consumption of snails carrying *A. cantonensis* larvae, causes eosinophilic meningoencephalitis[4, 11, 14-18]. As a consequence, *A. fulica* is attracting more and more attention in the fields of agricultural crops protection and human disease control.

To date, a variety of mollusk genomes have been analyzed and published, including two freshwater gastropods snails *Pomacea canaliculata*[19] and *Biomphalaria glabrata*[20]. However, no genome has been reported for terrestrial mollusks. *A. fulica* is considered to be a destructive terrestrial gastropod which poses a significant hazard to agriculture, the environment, biodiversity and human health. A chromosome-level genome of *A. fulica*could would provide crucial resources in the

population genetics and evolution studies based on genomic sequencing data aiming to discover the invasion and adaptation history of *A. fulica*. Furthermore, the genome could also be used to probe gene expression during important biological processes, such as gene expression patterns in various developmental stages and the interaction of *Angiostrongylus* and *A. fulica*. In this study we applied Illumina, PacBio and Hi-C techniques to construct the chromosome of *A. fulica*. The genome is the first terrestrial mollusk genome, providing an important reference for the molecular mechanisms underlying its broad environmental adaptability and the development of control strategy of the world-wide invasion.

Sample and sequencing

An adult snail (**Figure 1**), which was collected in Pingxiang city, Guangxi Autonomous Region, was used for reference genome construction. The snail was dissected and abdominal foot (17.4 g) and liver pancreas (40.4 g) tissues were collected and quickly frozen in liquid nitrogen overnight before transferring to -80 °C for storage. DNA was extracted using the traditional phenol/chloroform extraction method and was quality checked using agarose gel electrophoresis, meeting the requirement for library construction for the Illumina X Ten (Illumina Inc., San Diego, CA, USA) and for the PacBio Sequel (Pacific Biosciences of California, Menlo Park, CA, USA) sequencing platforms.

RNA was extracted from the pallium, liver, foot, spleen, stomach, gut, heart using TRIzol® Reagent (Life Technologies, USA). The RNA quality was checked using the Nanodrop ND-1000 spectrophotometer (LabTech, USA) and 2100 Bioanalyzer (Agilent Technologies, USA) with RNA integrity number lager than 8 (Supplemental Table S1 and Supplemental Figure S1). The RNA from each samples were equally mixed for the RNA sequencing on PacBio Sequel platform. Firstly, mRNA molecules were reversely transcribed to cDNA using Clontech SMARTer cDNA synthesis kit. After cDNA amplification and purification, two SMRTbell libraries of 0-4 kb and 4-10

kb were generated using the size selection in BluePippin Size Selection System (Pacific Biosciences of California, Menlo Park, CA, USA) and protocols suggested by manufacturer. The finale libraries were sequenced in the PacBio SEQUEL platform (Pacific Biosciences of California, Menlo Park, CA, USA), resulting 12,439,996 subreads totaling about 22.5 Gb PacBio long reads with average length longer than 1,801 bps. Subsequently, a total of 782,613 circular consensus sequences (CCS) were generated based on the subreads, and a number of 553,889 Full-length Non-chimeric sequences (FLNC) representing 23,726 gene loci were obtained, eventually. All aforementioned data processing were performed using SMRT Link v5.0 (www.pacb.com). Moreover, about 70.37% of the multi-exon FLNCs were really full-length sequences embracing all the exons of the gene locus predicted from the whole genome sequences.

Using the DNA from the abdominal foot, a library with the insertion length of 300 bp was constructed and sequenced using the Illumina sequencing platform according to the manufacturer’s protocol. About 202.23 Gb short reads were obtained using Illumina X Ten sequencing technology (**Table 1**), which was used for the following genome survey analysis, and for final base-level genome sequence correction. Meanwhile, four 20 kb libraries were constructed for PacBio Sequel sequencing. Using 16 sequencing SMRT cells, 104.6 Gb long reads were generated (**Table 1**). The mean and N50 lengths of the polymerases for sequencing cells ranged from 6.4 kb to 10.4 kb and from 12.3 kb to 20.3 kb for cells, respectively. Those long genomic DNA reads were then used for reference genome construction.

Genome features estimation from *K*mer method

With sequencing data from the Illumina platform, several genome characters could be evaluated for *A. fulica.* To ensure the quality of the analysis, ambiguous bases and low-quality reads were trimmed and filtered using the HTQC package (version

1.92.3)[21]. The following quality control were performed under the framework of HTQC. First, the quality of bases at two read ends were checked. Bases in sliding 5 bp windows were deleted if the average quality of the window was below phred quality score of 20. Second, reads were filtered if the average phred quality score were smaller than 20 or the read length was shorter than 75 bp. Third, the mate reads were also removed if the corresponding reads were filtered.

The quality-controlled reads were used for genome character estimation. We calculated the number of each 17-mer from the sequencing data using the jellyfish software (Jellyfish, RRID:SCR_005491; version 2.0)[22], and the distribution was analyzed with GCE software (GCE, RRID:SCR_017332; version 3)[23] and was shown in Supplemental Figure S2. We estimated the genome size of 2.12 Gb with the heterozygosity of 0.47% and repeat content of 71% in the genome. Previous studies revealed that repeat content varies in mollusks, and that repeat content is correlated with genome size[24]. The large genome size and high proportion of repeat contents of *A. fulica* provided additional supporting data for the statistical analysis. Moreover, 10,000 pairs of short reads were extracted randomly and were compared to the nt database and no obvious external contamination were found.

Genome assembly by third-generation long reads

After removing adaptor sequences in polymerases, 101.6 Gb subreads were generated for the following whole genome assembly. The average and N50 length of subreads reached 5.25 kb and 8.80 kb, respectively. To optimize the genome assembly using the PacBio sequencing data, we applied two packages in the assembly process, Canu v1.8 (Canu, RRID:SCR_015880) [25] and FALCON v0.2.2 (Falcon, RRID:SCR_016089) [26]. Canu package was first applied for the assembly using default parameters. As a result, a 1.93 Gb genome was constructed with 10,417 contigs and a contig N50 length of 662.40 kb. FALCON was also employed using the length_cutoff and pr_length_cutoff parameters of 10 kb and 8 kb, respectively. We

obtained 1.85 Gb genome with 8,585 contigs, with a contig N50 of 726.63 kb. We adopted the FALCON assembly as the reference genome for *A. fulica* (**Table 2**). Compared to the estimated genome size, the assembled version was relatively smaller, which may have resulted from the following two possible scenarios: the high repeat content of the genome, and the probably larger size estimated from the Kmer analysis. The genome sequences were subsequently polished, PacBio long reads utilizing arrow[27] and Illumina short reads using pilon[28] to correct base errors. The corrected genome was further applied for the following chromosome assembly construction using Hi-C data.

In situ **Hi-C library construction and chromosome assembly using Hi-C data**

Liver pancreas tissue of *A. fulica* was used for library construction for Hi-C analysis and the library was constructed using the identical method in previous studies[29]. Finally, the library was sequenced with 150 paired-end mode on the Illumina HiSeq X Ten platform (San Diego, CA, United States). From the Illumina sequencing platform, 1,313.87 million paired-end reads were obtained for the Hi-C library (**Table 1**). The reads were mapped to the above *A. fulica* genome with Bowtie2 [30], with two ends of paired reads being mapped to the genome separately. To increase the interactive Hi-C reads ratio, an iterative mapping strategy was performed as previous studies, and only read pairs with both ends uniquely mapped were used for the following analysis. From the alignment status of two ends, self-ligation, non-ligation and other sorts of invalid reads, including StartNearRsite, PCR amplification, random break, LargeSmallFragments and ExtremeFragments, were filtered out by Hi-Clib[31]. Through the recognition of restriction sites in sequences, contact counts among contigs were calculated and normalized.

According to previous karyotype analyses, *A. fulica* has 31 chromosomes[32]. By clustering the contigs using the contig contact frequency matrix, we were able to

correct some minor errors in the FALCON assembly results. Contigs with errors were broken into shorter contigs. We obtained 8,701 contigs, slightly more than the 8,585 contigs in the FALCON assembly. We successfully clustered these contigs into 31 groups in Lachesis[33] using the agglomerative hierarchical clustering method (**Figure 2**). Lachesis was further applied to order and orient the clustered contigs according to the contact matrix. As a result, 7,106 contigs were reliably anchored, ordered and orientated on chromosomes, accounting for 99.32% of the total genome bases. The first near chromosomal-level assembly of *A. fulica* was obtained with 8,211 contigs, a contig N50 of 721.0 kb and a scaffold N50 of 59.59 Mb (**Table 2** and **Table 3**).

Genome quality evaluation

We assessed the quality of genome of *A. fulica* after the assembly process. The quality evaluation was carried out in three aspects: continuity, completeness and the mapping rate of NGS data.

First of all, we compared the sequence number and contig N50 length of *A. fulica* with public genome of mollusks and found that our assembly has a high quality on contig and scaffold N50 among mollusk genomes. (**Table 3**) Traditional chromosomal genome assembly requires physical maps and genetic maps, which is enormously time- and labor-consuming. With Hi-C data analysis, we successfully assembled *A. fulica* genome into near chromosome-level with just one individual.

Second, the assembled genome was subjected to the BUSCO (version 3.0, metazoa_odb9)[34] to assess the completeness of the genome. About 91.7% of the BUSCO genes were identified in *A. fulica* genome, and more than 84.7% of the BUSCO genes were single-copy completed in our genome, illuminating a high level of completeness of the genome.

Third, NGS short reads were aligned to the genome using BWA package (BWA, RRID:SCR_010910; version 0.7.17)[35], and about 98.7% of paired reads were aligned to the genome, of which 98.24% were reads paired aligned.

Repeat element and gene annotation

Tandem Repeat Finder4.09 (TRF)[36] was used for repetitive element identification in the *A. fulica* genome. A *de novo* method applying RepeatModeler (RepeatModeler, RRID:SCR_015027) was used to detect transposable elements (TEs). The resulted *de novo* data, combined with known repeat library from Repbase[37], were used to identify TEs in the *A. fulica* genome by RepeatMasker4-0-8 (RepeatMasker, RRID:SCR_012954) [38] software. All repetitive elements were masked in the genome before protein-coding gene prediction.

Protein-coding genes in the *A. fulica* genome were annotated using the *de novo* program Augustus0.2.1 (Augustus, RRID:SCR_008417) [39]. Protein sequences of the closely related species including *Aplysia californica*, *Biomphalaria glabrata*，*Crassostrea gigas*，*Lottia gigantea* and *Patinopecten yessoensis*, were downloaded from the Ensembl database, and aligned to the *A. fulica* genome with TBLASTN2.6.0 (TBLASTN, RRID:SCR_011822). Full-length transcripts obtained using Iso-Seq were mapped to the genome using Genewise (GeneWise, RRID:SCR_015054) [40]. Finally, gene models predicted from all above methods were combined by MAKERv2.31.10 (MAKER, RRID:SCR_005309) [41], resulting in 23,726 protein-coding genes. The gene number, gene length, CDS length, exon length and intron length distribution were all comparable with the related mollusks (**Figure 3**).

To functionally annotate protein-coding genes in the *A. fulica* genome, we searched all predicted gene sequences to NCBI non-redundant nucleotide (NT) and protein (NR), Swiss-Prot databases by BLASTN (BLASTN, RRID:SCR_001598) [42] and BLASTX (BLASTX, RRID:SCR_001653) [43] utility. Blast2GO (Blast2GO, RRID:SCR_005828) [44] was also used to assign gene ontology (GO)[45] and Kyoto

Encyclopedia of Genes and Genomes (KEGG)[46] pathways. A threshold of e-value of 1e-5 was used for all BLAST applications. Finally, 22,858 (96.34%) genes were functionally annotated (**Table 4**).

Phylogenetic analysis of *A. fulica* with other mollusks

OrthoMCLv1.2 [47] was used to cluster gene families. First, proteins from *A. fulica* and the closely related mollusks, including *Aplysia californica*, *Biomphalaria glabrata*, *Crassostrea gigas*, *Lingula anatina*, *Lottia gigantea*, *Patinopecten yessoensis*, *Octopus bimaculoides*, *Helobdella robusta*, *Pomacea canaliculata*, and the outgroup, *Drosophila melanogaster*, were all-to-all blasted by BLASTP (BLASTP, RRID:SCR_001010) [43] utility with an e-value threshold of 1e-5. Only proteins from the longest transcript were used for genes with alternative isoforms. We identified 25,448 gene families for *A. fulica* and the related species, among them 675 single-copy orthologs families were detected.

Using single-copy orthologs, we could probe the phylogenetic relationships for the *A. fulica* and other mollusks. To this end, protein sequences of single-copy genes were aligned using CLUSTALX2.0 (Clustal X, RRID:SCR_017055) [48]. Guided by the protein multi-sequence alignment, the alignment of the coding DNA sequences (CDS) for those genes were generated and concatenated for the following analysis. The phylogenetic relationships were constructed using PhyML3.0 (PhyML, RRID:SCR_014629) [49] using the concatenated nucleotide alignment with the JTT+G+F model. The MCMCtree program in PAML4 [49] was used to estimate the species divergent time scales for the mollusks using approximate likelihood method and calibrated according to the fossil records. We found that *A. fulica* was most closely related to *Biomphalaria glabrata*, and the two species diverged from their common ancestor about 242 million years ago (MYA) (**Figure 4**).

Conclusion

We reconstructed the first chromosome level assembly for *A. fulica* using an

integrated sequencing strategy combining PacBio, Illumina and Hi-C technologies. Using the long reads from the PacBio Sequel platform and short reads from the Illumina X Ten platform, we successfully constructed contig assembly for *A. fulica*. Leveraging contact information among contigs from Hi-C technology, we further improved the assembly to near chromosome-level quality (**Table 3** and **Figure 2**). We predicted 23,726 protein-coding genes in the *A. fulica* genome and 22,858 of genes were functionally annotated with putative functions. With 675 single-copy orthologs from *A. fulica* and other related mollusks, we constructed the phylogenetic relationship of these mollusks, and found that *A. fulica* might have diverged from its common ancestor of *Biomphalaria glabrata* around 177.1-187.1 MYA. Given the increasing interest in mollusk genomic evolution and the biological importance of *A. fulica* as an invasive animal, our genomic and transcriptome data will provide valuable genetic resources for follow-on functional genomics investigations by the research community.

Ethics Statement

This study was approved by the Animal Care and Use committee of National Institute of Parasitic Diseases, Chinese Center for Disease Control and Prevention.

Abbreviation

CCS: circular consensus sequences; CDS: coding DNA sequences; FLNC: Full-length Non-chimeric sequences; GO: gene ontology; KEGG: Kyoto Encyclopedia of Genes and Genomes; MYA: million years ago; TE: transposable elements

Availability of supporting data

The Illumina, PacBio and Hi-C sequencing data are available from NCBI via the accession number of SRR8369706, SRR8369311 and SRR8371669, respectively. The Illumina transcriptome sequencing data were deposited to NCBI via the

accession number of SRR8371872 and SRR8371873. The genome, annotation and intermediate files were uploaded to *GigaScience* GigaDB Database [66].

Competing interests

The authors declare that they have no competing interests.

Acknowledgement

This work was supported by the National Key Research and Development Program of China (No. 2016YFC1200500 and 2016YFC1202000). The authors thank Frasergen Bioinformatics for providing technique supports for this work.

Author Contributions

Z.X, H.W and X.N conceived the project. G.Y, Z.Y, L.Q collected the samples and extracted the genomic DNA. G.Y, Z.Y and L.Q performed the genome assembly and data analysis. G.Y, Z.X, H.W and X.N wrote the paper.

References

1. Schreurs J. *Investigations on the biology, ecology and control of Giant African Snail 290 in West New Guinea*. 1963. Manokwari Agricultural Research Station.
2. Albuquerque FS, Peso-Aguiar MC and Assunção-Albuquerque MJ. Distribution,feeding behavior and control strategies of the exotic land snail Achatinafulica (Gastropoda:Pulmonata) in the Northeast of Brazil. BrazJBiol. 2008;68:6.
3. Thiengo SC, Fernandez MA, Torres EJ, Coelho PM and Lanfredi RM. First record of anematode Metastrongyloidea (Aelurostrongylus abstrusus larvae) in Achatina (Lissachatina) fulica (Mollusca,Achatinidae) in Brazil. J Invertebr Pathol. 2008;98:6.
4. Lv S, Zhang Y and Liu HX. Invasive Snails and an Emerging Infectious Disease: Results from the First National Survey on Angiostrongylus cantonensis in China. BioOne. 2009; doi:10.1371/journal.pntd.0000368.
5. Cowie RH. *Non-indigenous land and freshwater molluscs in the islands of the Pacific: Conservation impacts and threats*. 2000.
6. Cowie RH. Can snails ever be effective and safe biocontrol agents? Int J Pest Manage. 2001;47:18.
7. Cowie RH and Robinson DG. Pathways of introduction of nonindigenous land and freshwater snails and slugs. Washington DC: Island Press; 2003.
8. Kotangale JP. Giant African snail (Achatina fulica Bowdich). 2011;J Environ Sci Eng 53:6.
9. Raut SK and Barker GM. Achatina fulica Bowdich and Other Achatinidae as Pests in Tropical Agriculture. UK: CABI International; 2002.
10. Jarreit VHC. The spread of the snail Achatina fulica to south China. Hong Kong Nat. 1931;2:3.

11. Shan L, Yi Z and Peter S. Emerging Angiostrongyliasis in Mainland China. Emerging Infectious Diseases. 2008;14 1:4.

12. Lowe S, Browne SM, Boudjrlas S and De Poorter M. 100 of the world's worst invasive alien species: A selection from the global invasive species database. The Invasive Species Specialists Group of the Species Survival Commission of the World Conservation Union. Auckland: Hollands Printing; 2000.

13. Mead AR. Pulmonates volume 2B. Economic malacology with particular reference to Achatina fulica. London: Academic Press; 1979.

14. Alicata JE. The discovery of Angiostrongylus cantonensis as a cause of human eosinophilic meningitis. Parasitol Today. 1991;7 6:151-3.

15. Prociv P, Spratt DM and Carlisle MS. Neuro-angiostrongyliasis: unresolved issues. Int J Parasitol. 2000;30 12-13:1295-303.

16. Deng ZH, Zhang QM, Huang SY and Jones JL. First provincial survey of Angiostrongylus cantonensis in Guangdong Province, China. Trop Med Int Health. 2012;17:4.

17. Maldonado JA, Simoes RO, Oliveira AP, Motta EM, Fernandez MA, Pereira ZM, et al. First report of Angiostrongylus cantonensis (Nematoda: Metastrongylidae) in Achatina fulica (Mollusca: Gastropoda) from Southeast and South Brazil. Mem Inst Oswaldo Cruz. 2010;105:4.

18. Vitta A, Polseela R, Nateeworanart S and Tattiyapong M. Survey of Angiostrongylus cantonensis in rats and giant African land snails in Phitsanulok Province, Thailand. Asian Pac J Trop Med. 2011;4:3.

19. Liu C, Zhang Y, Ren Y, Wang H, Li S, Jiang F, et al. The genome of the golden apple snail *Pomacea canaliculata* provides insight into stress tolerance and invasive adaptation. GigaScience. 2018;7 9 doi:10.1093/gigascience/giy101.

20. Adema CM, Hillier LW, Jones CS, Loker ES, Knight M, Minx P, et al. Whole genome analysis of a schistosomiasis-transmitting freshwater snail. Nature communications. 2017;8:15451. doi:10.1038/ncomms15451.

21. Neff KL, Argue DP, Ma AC, Lee HB, Clark KJ and Ekker SC. Mojo Hand, a TALEN design tool for genome editing applications. BMC Bioinformatics. 2013;14:1. doi:10.1186/1471-2105-14-1.

22. Marcais G and Kingsford C. A fast, lock-free approach for efficient parallel counting of occurrences of k-mers. Bioinformatics. 2011;27 6:764-70. doi:10.1093/bioinformatics/btr011.

23. Binghang Liu YS, Jianying Yuan,Xuesong Hu,Hao Zhang,Nan Li,Zhenyu Li,Yanxiang Chen,Desheng Mu,Wei Fan. Estimation of genomic characteristics by analyzing k-mer frequency in de novo genome projects. Quantitative Biology. 2013;35:62-7.

24. Murgarella M, Puiu D, Novoa B, Figueras A, Posada D and Canchaya C. A First Insight into the Genome of the Filter-Feeder Mussel Mytilus galloprovincialis. PloS one. 2016;11 3:e0151561. doi:10.1371/journal.pone.0151561.

25. Koren S, Walenz BP, Berlin K, Miller JR, Bergman NH and Phillippy AM. Canu: scalable and accurate long-read assembly via adaptive k-mer weighting and repeat separation. Genome Res. 2017;27 5:722-36. doi:10.1101/gr.215087.116.

26. Chin CS, Peluso P, Sedlazeck FJ, Nattestad M, Concepcion GT, Clum A, et al. Phased diploid genome assembly with single-molecule real-time sequencing. Nat Methods. 2016;13 12:1050-4. doi:10.1038/nmeth.4035.

27. Chin C-S, Alexander DH, Marks P, Klammer AA, Drake J, Heiner C, et al. Nonhybrid, finished microbial genome assemblies from long-read SMRT sequencing data. Nature methods. 2013;10 6:563.
28. Walker BJ, Abeel T, Shea T, Priest M, Abouelliel A, Sakthikumar S, et al. Pilon: an integrated tool for comprehensive microbial variant detection and genome assembly improvement. PloS one. 2014;9 11:e112963.
29. Gong G, Dan C, Xiao S, Guo W, Huang P, Xiong Y, et al. Chromosomal-level assembly of yellow catfish genome using third-generation DNA sequencing and Hi-C analysis. Gigascience. 2018; doi:10.1093/gigascience/giy120.
30. Langmead B, Trapnell C, Pop M and Salzberg SL. Ultrafast and memory-efficient alignment of short DNA sequences to the human genome. Genome Biol. 2009;10 3:R25. doi:10.1186/gb-2009-10-3-r25.
31. Burton JN, Adey A, Patwardhan RP, Qiu R, Kitzman JO and Shendure J. Chromosome-scale scaffolding of de novo genome assemblies based on chromatin interactions. Nature biotechnology. 2013;31 12:1119.
32. Sun T. Chromosomal studies in three land snails. Sinozoologia. 1995;12:154-62.
33. Near TJ, Dornburg A, Eytan RI, Keck BP, Smith WL, Kuhn KL, et al. Phylogeny and tempo of diversification in the superradiation of spiny-rayed fishes. Proceedings of the National Academy of Sciences of the United States of America. 2013;110 31:12738.
34. Simão FA, Waterhouse RM, Ioannidis P, Kriventseva EV and Zdobnov EM. BUSCO: assessing genome assembly and annotation completeness with single-copy orthologs. Bioinformatics. 2015;31 19:3210-2.
35. Li H and Durbin R. Fast and accurate short read alignment with Burrows–Wheeler transform. bioinformatics. 2009;25 14:1754-60.
36. Benson G. Tandem repeats finder: a program to analyze DNA sequences. Nucleic Acids Res. 1999;27 2:573-80.
37. Bao W, Kojima KK and Kohany O. Repbase Update, a database of repetitive elements in eukaryotic genomes. Mob DNA. 2015;6:11. doi:10.1186/s13100-015-0041-9.
38. Chen N. Using RepeatMasker to identify repetitive elements in genomic sequences. Current protocols in bioinformatics. 2004;5 1:4.10. 1-4.. 4.
39. Stanke M, Keller O, Gunduz I, Hayes A, Waack S and Morgenstern B. AUGUSTUS: ab initio prediction of alternative transcripts. Nucleic acids research. 2006;34 suppl_2:W435-W9.
40. Birney E, Clamp M and Durbin R. GeneWise and genomewise. Genome research. 2004;14 5:988-95.
41. Cantarel BL, Korf I, Robb SM, Parra G, Ross E, Moore B, et al. MAKER: an easy-to-use annotation pipeline designed for emerging model organism genomes. Genome research. 2008;18 1:188-96.
42. Gertz EM, Yu YK, Agarwala R, Schaffer AA and Altschul SF. Composition-based statistics and translated nucleotide searches: improving the TBLASTN module of BLAST. BMC Biol. 2006;4:41. doi:10.1186/1741-7007-4-41.
43. Camacho C, Coulouris G, Avagyan V, Ma N, Papadopoulos J, Bealer K, et al. BLAST+: architecture and applications. BMC Bioinformatics. 2009;10:421. doi:10.1186/1471-2105-10-421.
44. Conesa A, Götz S, García-Gómez JM, Terol J, Talón M and Robles M. Blast2GO: a universal

tool for annotation, visualization and analysis in functional genomics research. Bioinformatics. 2005;21 18:3674-6.
45. Consortium GO. The Gene Ontology (GO) database and informatics resource. Nucleic acids research. 2004;32 suppl_1:D258-D61.
46. Kanehisa M and Goto S. KEGG: kyoto encyclopedia of genes and genomes. Nucleic acids research. 2000;28 1:27-30.
47. Li L, Stoeckert CJ and Roos DS. OrthoMCL: identification of ortholog groups for eukaryotic genomes. Genome research. 2003;13 9:2178-89.
48. Thompson JD, Gibson TJ and Higgins DG. Multiple sequence alignment using ClustalW and ClustalX. Current protocols in bioinformatics. 2003; 1:2.3. 1-2.3. 22.
49. Guindon S, Lethiec F, Duroux P and Gascuel O. PHYML Online—a web server for fast maximum likelihood-based phylogenetic inference. Nucleic acids research. 2005;33 suppl_2:W557-W9.
50. Zhang G, Fang X, Guo X, Li L, Luo R, Xu F, et al. The oyster genome reveals stress adaptation and complexity of shell formation. Nature. 2012;490 7418:49-54. doi:10.1038/nature11413.
51. Takeuchi T, Kawashima T, Koyanagi R, Gyoja F, Tanaka M, Ikuta T, et al. Draft genome of the pearl oyster Pinctada fucata: a platform for understanding bivalve biology. DNA research : an international journal for rapid publication of reports on genes and genomes. 2012;19 2:117-30. doi:10.1093/dnares/dss005.
52. Takeuchi T, Koyanagi R, Gyoja F, Kanda M, Hisata K, Fujie M, et al. Bivalve-specific gene expansion in the pearl oyster genome: implications of adaptation to a sessile lifestyle. Zoological letters. 2016;2:3. doi:10.1186/s40851-016-0039-2.
53. Du X, Fan G, Jiao Y, Zhang H, Guo X, Huang R, et al. The pearl oyster *Pinctada fucata martensii* genome and multi-omic analyses provide insights into biomineralization. GigaScience. 2017;6 8:1-12. doi:10.1093/gigascience/gix059.
54. Mun S, Kim YJ, Markkandan K, Shin W, Oh S, Woo J, et al. The Whole-Genome and Transcriptome of the Manila Clam (Ruditapes philippinarum). Genome biology and evolution. 2017;9 6:1487-98. doi:10.1093/gbe/evx096.
55. Wang S, Zhang J, Jiao W, Li J, Xun X, Sun Y, et al. Scallop genome provides insights into evolution of bilaterian karyotype and development. Nature ecology & evolution. 2017;1 5:120. doi:10.1038/s41559-017-0120.
56. Schell T, Feldmeyer B, Schmidt H, Greshake B, Tills O, Truebano M, et al. An annotated draft genome for *Radix auricularia* (Gastropoda, Mollusca). Genome biology and evolution. 2017; doi:10.1093/gbe/evx032.
57. Albertin CB, Simakov O, Mitros T, Wang ZY, Pungor JR, Edsinger-Gonzales E, et al. The octopus genome and the evolution of cephalopod neural and morphological novelties. Nature. 2015;524 7564:220-4. doi:10.1038/nature14668.
58. Simakov O, Marletaz F, Cho SJ, Edsinger-Gonzales E, Havlak P, Hellsten U, et al. Insights into bilaterian evolution from three spiralian genomes. Nature. 2013;493 7433:526-31. doi:10.1038/nature11696.
59. Kenny NJ, Namigai EK, Marletaz F, Hui JH and Shimeld SM. Draft genome assemblies and predicted microRNA complements of the intertidal lophotrochozoans *Patella vulgata* (Mollusca, Patellogastropoda) and Spirobranchus (Pomatoceros) lamarcki (Annelida, Serpulida). Marine genomics. 2015;24 Pt 2:139-46. doi:10.1016/j.margen.2015.07.004.

60. Barghi N, Concepcion GP, Olivera BM and Lluisma AO. Structural features of conopeptide genes inferred from partial sequences of the *Conus tribble*i genome. Molecular genetics and genomics : MGG. 2016;291 1:411-22. doi:10.1007/s00438-015-1119-2.

61. Uliano-Silva M, Dondero F, Dan Otto T, Costa I, Lima NCB, Americo JA, et al. A hybrid-hierarchical genome assembly strategy to sequence the invasive golden mussel, *Limnoperna fortunei*. GigaScience. 2018;7 2 doi:10.1093/gigascience/gix128.

62. Sun J, Zhang Y, Xu T, Zhang Y, Mu H, Zhang Y, et al. Adaptation to deep-sea chemosynthetic environments as revealed by mussel genomes. Nature ecology & evolution. 2017;1 5:121. doi:10.1038/s41559-017-0121.

63. Jiao W, Fu X, Dou J, Li H, Su H, Mao J, et al. High-resolution linkage and quantitative trait locus mapping aided by genome survey sequencing: building up an integrative genomic framework for a bivalve mollusc. DNA research : an international journal for rapid publication of reports on genes and genomes. 2014;21 1:85-101. doi:10.1093/dnares/dst043.

64. Luo YJ, Takeuchi T, Koyanagi R, Yamada L, Kanda M, Khalturina M, et al. The Lingula genome provides insights into brachiopod evolution and the origin of phosphate biomineralization. Nature communications. 2015;6:8301. doi:10.1038/ncomms9301.

65. Li C, Liu X, Liu B, Ma B, Liu F, Liu G, et al. Draft genome of the Peruvian scallop Argopecten purpuratus. GigaScience. 2018;7 4 doi:10.1093/gigascience/giy031.

66. Guo Y; Zhang Y; Liu Q; Huang Y; Mao G; Yue Z; Abe EM; Li J; Wu Z; Li S; Zhou X; Hu W; Xiao N (2019): Supporting data for "A chromosomal-level genome assembly for the giant African snail *Achatina fulica*" GigaScience Database. http://dx.doi.org/10.5524/100647

Tables and Figures

Table 1: Sequencing data generated for *A.fulica* genome assembly and annotation

Library type	Platform	Library size (bp)	Data size (Gb)	Application
Short reads	HiSeq X Ten	350	202.24	Genome survey and genomic base correction
Long reads	PacBio SEQUEL	20,000	101.63	Genome assembly
Hi-C	HiSeq X Ten	300-500	199.73	Chromosome construction

Table 2: Statistics for genome assembly of *A. fulica*

Sample ID	Length		Number	
	Contig** (bp)	Scaffold (bp)	Contig**	Scaffold
Total	1,852,282,574	1,855,883,074	8,211	1,010
Max	5,947,392	116,558,012	-	-
N50	721,038	59,589,303	697	13
N60	538,883	58,013,356	995	16
N70	399,612	53,672,006	1,396	20
N80	268,901	50,673,968	1,957	23
N90	141,756	44,109,545	2,888	27

The two stars (**) means the ultimate contigs since they were probably modified during the Hic step.

Table 3 Summary of the genome of *A. fulica* and other published mollusk genomes.

Species	Size* (Mb)	Contig N50(kb)	Scaffold N50(kb)
Achatina fulica (this study)**	2,120	721	59,590
Pomacea canaliculata[19]**	570	995	38,000
Crassostrea gigas[50]	545	7.5	401
Pinctada fucata[51]	1,150	1.6	14.5
Pinctada fucata new[52]	1,150	21	324
Pinctada fucata V2[53]	1,150	21	167
Biomphalaria glabrata[20]	931	7.3	48
Ruditapes philippinarum[54]	1,370	3.3	32.7
Patinopecten yessoensis[55]**	1,430	38	41,000
Radix auricularia[56]	1,600	0.324	578
Octopus bimaculoides[57]	2,800	5.4	470
Mytilus galloprovincialis[24]	1,600	2.6	2.9
Lottia gigantea[58]	420	96	1,870
Patella vulgata[59]	1,460	3.1	3.1
Aplysia californica	1,760	9.6	917
Conus tribblei[60]	2,760	0.85	215
Limnoperna fortunei[61]	1,600	10	312
Bathymodiolus platifrons[62]	1,640	13.2	343
Modiolus philippinarum[62]	2,380	19.7	100.2
Chlamys farreri[63]	1,200	1.2	1.5
Lingula anatina[64]	463	55	294
Argopecten prupruatus[65]	885	80.1	1,020

* Estimated size of the genome

** Genomes assembled into near chromosomal level

Table 4: Statistics for genome annotation of *A. fulica*

Database	Number	Percent
InterPro	16,252	68.50
GO	12,101	51.00
KEGG ALL	21,325	89.88
KEGG KO	10,161	42.83
Swissprot	17,050	71.86
TrEMBL	22,403	94.42
NR	22,553	95.06
Total	23,726	

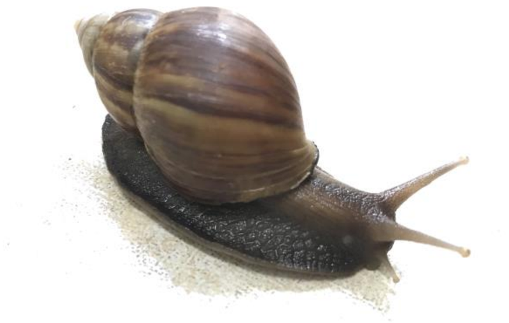

Figure 1. ***A. fulica*** individual **used for genome sequencing and assembly.**

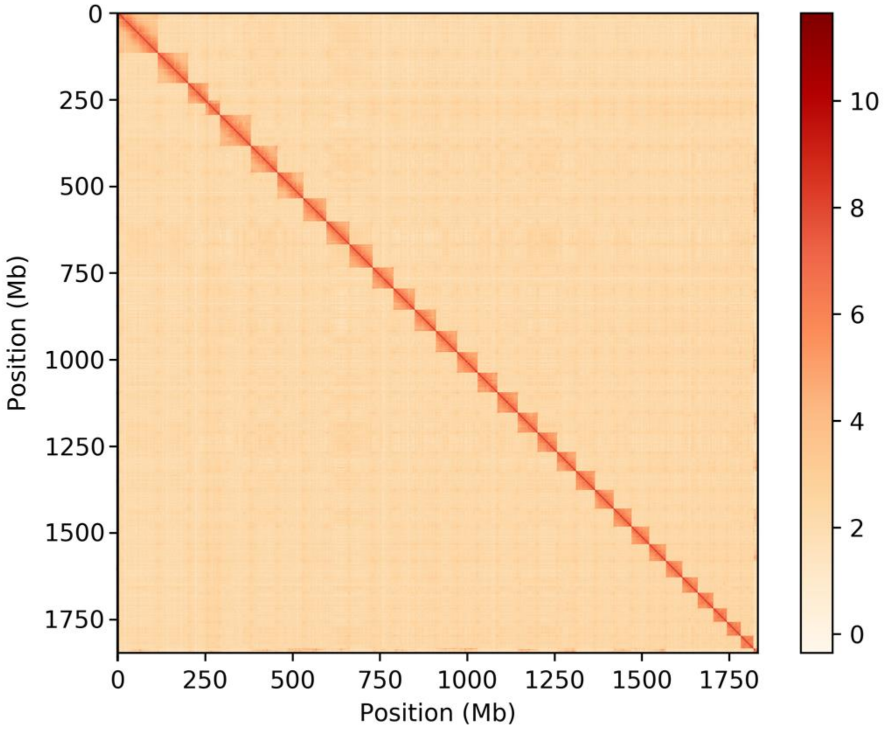

Figure 2. Contact matrix generated from the Hi-C data analysis showing sequence interactions in chromosomes. The logarithm of the contact density were showed in the color bar.

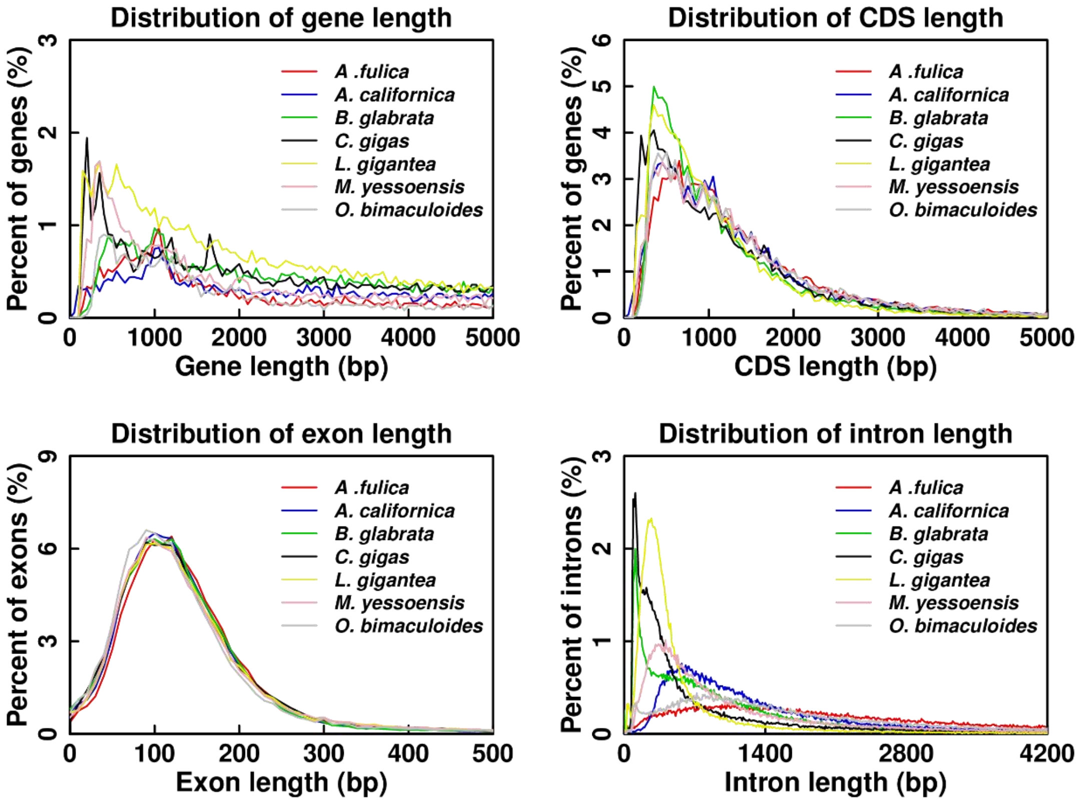

Figure 3. Length distribution comparison on total gene, CDS, exon, and intron of annotated gene models of *A. fulica* with other closely related insect species.The comparison of length distribution of genes (A), CDS (B), exon (C) and intron (D) for *A. fulica* to those in *A.californica* , *B. glabrata* , *C. gigas* , *L. gigantea* , *P. yessoensis*and *O. bimaculoides*.

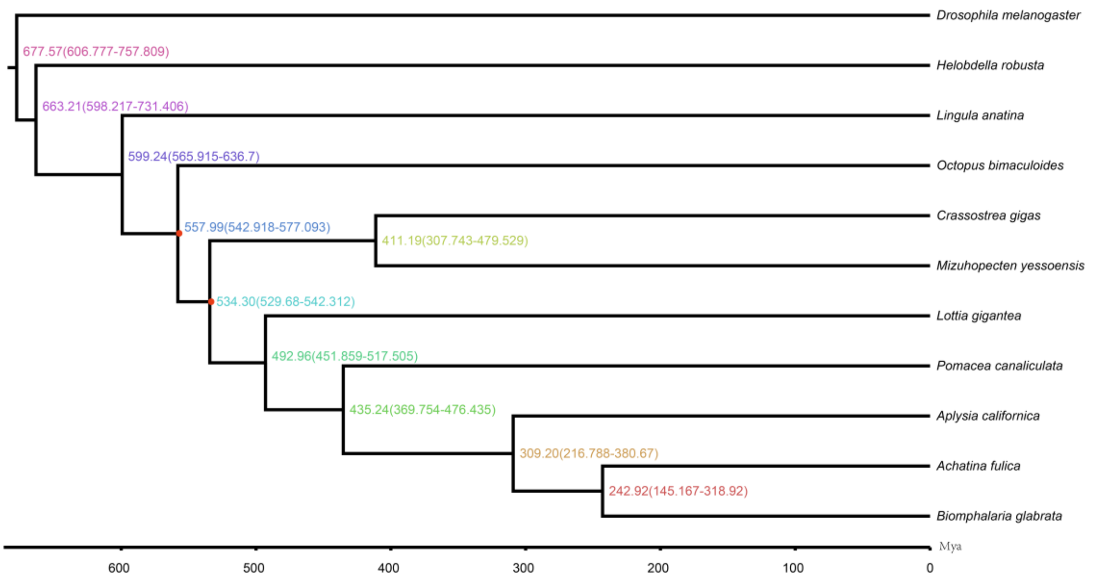

Figure 4. Phylogenetic relationship between *A. fulica* and related species. The divergence time (million years ago, Mya) and the 95% confidential intervals are labeled at branch sites and the red dots in the tree denotes the fossil recalibration sites with the maximum and minimum age of Bivalve/gastropod divergence were 543 and 530 Mya, and the maximum age of Mollusk crown group divergence was 549 Mya.

Click here to access/download
Supplementary Material
supplementary_information.docx
