# Supplementary material for: A chromosomal-level genome assembly for the giant African snail Achatina fulica
Source: Gigascience. 2019 Oct 21;8(10):giz124. doi: 10.1093/gigascience/giz124 (PMC6802634; doi:10.1093/gigascience/giz124)
Supplement: giz124_Supplemental_File [file giz124_supplemental_file.docx]

**Supplementary Information for**

**A chromosomal-level genome assembly for** **the giant African snail *Achatina fulica***

Guo Yunhai^1,2, #^，Zhang Yi^1,2, #^，Liu Qin^1,2^，Huang Yun^1,2^，Mao Guangyao^1,2^，Yue Zhiyuan^1,2^，Eniola M. Abe^1,2^，Li Jian^3^，Wu Zhongdao^4^，Li Shizhu^1,2^，Zhou Xiaonong^1,2^，Hu Wei^1,2,3,^*，Xiao Ning^1,2,^*

^1^National Institute of Parasitic Diseases, Chinese Center for Disease Control and Prevention

^2^Key Laboratory of Parasite and Vector Biology, Ministry of Health, Shanghai, China

^3^Department of Microbiology and Microbial Engineering，School of Life Sciences，Fudan University，Shanghai 200438，China

^4^Department of Parasitology, Zhongshan School of Medicine, Sun Yat-sen University, Guangzhou 510080, China

**Supplemental Table S1. Summary of RNA quality of samples.** The high-quality samples highlighted by red for the PacBio library construction and sequencing.

| **SampleID** | **Tissue** | **RIN** | **28S/18S** | **OD260/280** | **Concentration**  **(ng/ul)** | **Volumn (ul)** | **Total (ug)** |
| --- | --- | --- | --- | --- | --- | --- | --- |
| D2-7 | stomach | 2.8 | 0 | 1.7 | 34.9 | 40 | 1.4 |
| D2-9 | gut | 3.5 | 0 | 1.87 | 111.7 | 40 | 4.5 |
| A2' | whole | 4.2 | 0.7 | 2 | 452.2 | 40 | 18.1 |
| D2-3' | liver | 5 | 0.8 | 1.85 | 673 | 40 | 26.9 |
| D2-1' | foot | 5.5 | 0.7 | 2.14 | 129.5 | 30 | 3.9 |
| D2-5 | gut | 8.6 | 0.1 | 2.12 | 722.4 | 40 | 28.9 |
| C1-1 | foot | 9.1 | 0.1 | 1.96 | 75.6 | 40 | 3.0 |
| B2-1 | foot | 9.4 | 0 | 1.96 | 125.2 | 40 | 5.0 |
| D2-3 | liver | 9.1 | 0.1 | 1.75 | 911.7 | 40 | 36.5 |
| D2-10 | lung | 9.5 | 0 | 2 | 93.9 | 40 | 3.8 |
| D2-6 | stomach | 9 | 0 | 1.66 | 11.7 | 40 | 0.5 |
| D2-14 | stomach | 9.5 | 0.1 | 1.77 | 60.9 | 40 | 2.4 |
| D2-1 | foot | 9.6 | 0.1 | 1.9 | 63.9 | 40 | 2.6 |
| D2-11 | heart | 9.6 | 0 | 1.94 | 72.6 | 40 | 2.9 |
| D2-4 | spleen | 9.7 | 0.1 | 1.69 | 86 | 40 | 3.4 |
| D2-2' | pallium | 9.6 | 0 | 2.01 | 54.2 | 15 | 0.8 |
| D2-2 | pallium | 10 | 0.1 | 2.01 | 67.6 | 40 | 2.7 |

**Supplemental Figure S1. Bioanalyzer summary reports for samples used in the transcriptome sequencing.**


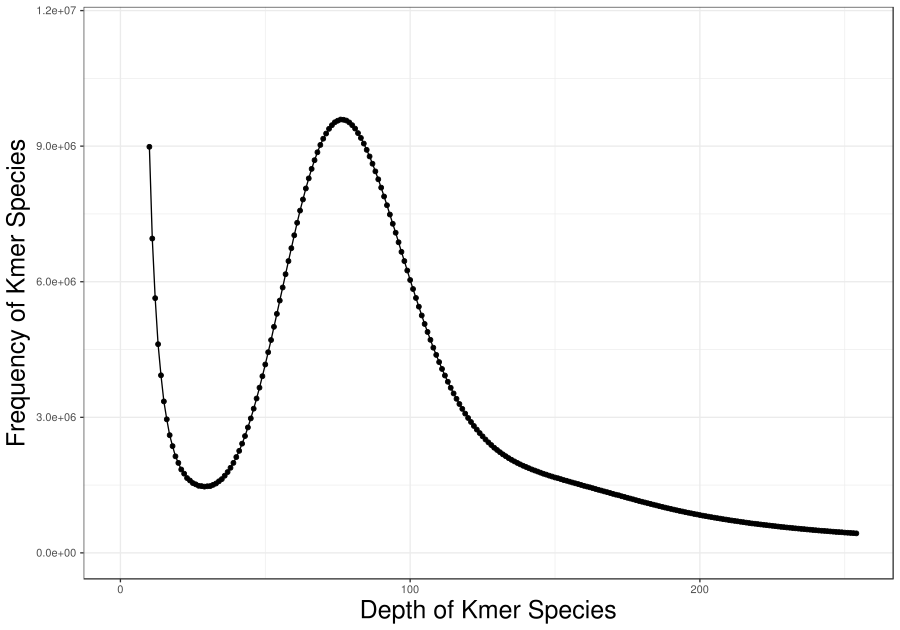


**Supplemental Figure S2. The distribution of kmer species estimated for A. fulica.** The total number of kmer species is 178,847,565,204, with the peak value(depth) is 76.
