# Supplementary material for: A chromosomal-level genome assembly for the giant African snail Achatina fulica
Source: Gigascience. 2019 Oct 21;8(10):giz124. doi: 10.1093/gigascience/giz124 (PMC6802634; doi:10.1093/gigascience/giz124)
Supplement: giz124_Response_to_Reviewer_Comments_Original_Submission [file giz124_response_to_reviewer_comments_original_submission.pdf]

Author's Response To Reviewer Comments

Close

Reviewer reports:

Reviewer #1: In this study the authors sequenced the genome of the giant African snail Achatina fulica using short and lo
read technologies as well as a Hi-C scaffolding method, and succeeded to develop chromosomal-level genome assembly. I
think the data will contribute to our understanding of the biology of the species.
Reply: We thanks a lot for the reviewer's positive comments for our manuscript.

At the same time I found description of methods is not sufficient in the present manuscript, therefore it should be revised
before publication.
In the Introduction the authors mentioned that it is important to study the biology of A. chatina because the species is one
of the most threatening invasive species, and is the intermediate host of Angiostrongylus. However, I could not find how t
present chromosomal-level genome assembly is useful to address these issues. I would like to request the authors to disc
the point more specifically. This will emphasize the importance of the study.
Reply: Thanks a lot for the suggestion. The chromosome genome of A. chatina could provide an important framework in th
following population genetics using next-generation sequencing data. Meanwhile, the predicted genes in the genome of A.
chatina could be used for the transcriptome analysis for the interaction of Angiostrongylus and A. chatina. We have added
the information into the revised manuscript. (lines 85-91 in the revised ms)

The information about transcriptome is absent despite the data might be used for gene model prediction (lines 206-207).
The authors should describe in detail about the transciptome. For example, from which tissues was RNA extracted? How w
the quality of the RNA? How was the stats of RNA-Seq (number of reads, average length, etc.)? In addition, mapping rate
the transcriptome to the genome assembly and gene models will be informative to evaluate the completeness of the
assembly and model prediction, respectively.
Reply: Thanks for the reviewer's reminding. The detailed information for the RNA sequencing has been added in the revise
manuscript. (lines 106-124 in the revised ms)

Lines 178-180
High rate of heterozygosity (>1%) have been reported in bivalve genomes (oysters, scallops, etc.) but not the case in
gastropods.
Reply: Thanks for the reviewer's reminding. Previous genome study of Pomacea canaliculata, belonging to gastropods,
revealed the high heterozygosity among 1%-2%. (doi: 10.1093/gigascience/giy101) To avoid the confusion, we have
deleted the sentence in the manuscript.

Fig. 3
I would suggest to show the genome assembly comparison data in a table, not in a scatter plot.
In general, scatter plot is used to see the correlation between two variables. This figure is not adequate to compare genor
assemblies because 1) correlation between contig and scaffold N50s is not meaningful 2) most of the dots are put at the
lower left and indistinguishable.
In addition, references should be cited when the authors used these genome data in the study.
Reply: Thanks a lot for the suggestion. We have changed the Figure 3 into Table 3 and added the references in the revise
manuscript.

Lines 232-235, Fig. 5
What kinds of fossil record were used for molecular clock calibration? Honestly speaking, I cannot believe the result (Fig.5
showing Spiralia diverged from Ecdysozoa 831 Mya (200 million years before the Ediacaran Period).
Reply: Thank you very much for the reminding. However, we re-estimated the divergence time among these species using
the records for Protostomia and Mollusca downloaded from www.timetree.org and obtained the similar results (the figure
below was downloaded from the place). Thus we believe the results might be reliable. The new results and the calibration
information were updated in the revised ms. (lines 258-261 and fig 5)

Version information of all software used are needed.
Reply: Thank you very much for the reminding. All the version information available has been added in the revised ms.

Reviewer #2: Please see attached Review.
Overall, this appears to be a well put together genome encompassing large amounts of data from different sources, includ long reads from PacBio and additional scaffolding from Hi-C. It is quite well presented and I'm sure this work will be usefu the community as a genomics resource. Nonetheless there are a few issues that I'd like to see resolved before the manuscript can be accepted for publication or the assembly is released into the public repositories.

Major comments
Contamination. There is no mention in the text of filters for possible contamination from non-target organisms in the sequencing data. I consider such an analysis to be a vital and necessary component of any genome project, to eliminate (much as possible) errors from contaminating sequencing reads in sequence databases. Tools such as Blobtools (https://drl.github.io/blobtools/) are easy to implement and are highly informative as to the quality of the raw data and th final genome.
Reply: Thanks for the reviewer's reminding. Actually we did the contamination analysis at the step Survey since the DNA samples in Survey and Assembly was identical. In the survey step, we randomly extracted 10,000 pairs of short reads, an compared them to the nt database, and find no obvious external contamination from other species. This method has been described elsewhere (https://doi.org/10.1016/j.molp.2014.12.011) and we did not mention it since it performed as expected. The result of contamination analysis has been added in the revised ms (lines 154-155 in the revised ms).

Kmer analysis. There is much discussion about estimation of genome size from kmer analysis, but there is no kmer spectr presented. I would find this figure much more informative and useful than some of the figures that are included (e.g. 2 an 3).
Reply: Thank you very much for your suggestions. The kmer spectra has been added in the revised version (Figure 2).

Heterozygosity. Related to the above point: how did the authors resolve any regions containing heterozygous sites in the assembly? E.g., divergent allelic regions that might be co-assembled and both present in the final scaffolds?
Reply: Thank you very much for you reminding. By mapping the subreads back to the genome, we estimated the sequenc depth for each region of the assembly and the results were shown below (the GC content were also shown, 10k window). shows that the distribution of the depth is unimodal, which means that almost all sites were homozygous, actually the heterozygosity of the species is not very high (<0.5%). And if there are too much divergent allelic regions, two peaks will obvious.
-
Transcriptome / RNA-seq. Table 1 shows 22.5Gb of transcriptomic reads but very little information is given about these da How they were generated and filtered, and then how they were used during the annotation process needs more details.
Reply: Thank you very much for your reminding. The information has been added in the revised version (lines 106-124, li 229 in the revised ms).

Language. Overall the manuscript is well written, but there were many cases of grammatical errors and/or small typos, to many to catch them all in the minor comments below (I mostly stopped after the abstract). Thus, the manuscript would benefit from a proof-read to correct these small mistakes in English, it would not be a big task.
Reply: Thank you very much for your reminding. We corrected errors and typos thorough the manuscript in the revised version.

Finally, what is the criteria for "chromosome level assembly", a description that is used throughout the manuscript for the genome? I find it a bit puzzling that the final assembly has ~1000 scaffolds (~8000 contgs) and is described as chromoso level, but we are told there are 31 chromosomes for this species. By all accounts the authors have done a good job with such a large and repeat-rich genome, but to call it chromosome level is perhaps a bit misleading.
Reply: Thank you very much for your reminding. At last, based on the Hic technology, more than 99% of the total length were reliably anchored, ordered and orientated on the 31 chromosomes using Lachesis, and result in a scaffold N50 of 59. Mb of the assembly. This is comparable to the size of the chromosome, thus we call it chromosome level.

Minor comments
Line 28: "also called THE giant African snail…"
Reply: We have added the "the" in the revised ms.

Line 29 and elsewhere: the word "greedy" is a bit casual; suggest to use "extensive", "voracious" or other synonym
Reply: We have changed it into "voracious".

Line 30: "reproductIVE capacity"
Reply: We have corrected the mistake.

Line 30: "caused A world-wide…"
Reply: We have added the "a" in the revised version.

Line 32: “a pest THAT IS ABLE TO damage the agricultural crops”
Reply: We have corrected it according to your instructions.

Line 33: “many parasites THAT CAN threaten”
Reply: We have corrected it according to your instructions.

Line 34: “hindering the genetic”
Reply: We have deleted the “the” in the revised ms.

Line 37: “genome size TO BE 2.12 Gb”
Reply: We have changed it into “to be” in the revised ms.

Line 52: sentence has numerous grammatical errors, please rewrite.
Reply: We have rewritten it in the revised ms.

Line 66: “direct or indirect” – which is it?
Reply: We have changed it into “direct and indirect”, which means both.

Line 71: the link provided is in Chinese and is difficult to navigate to the aforementioned list of invasive species
Reply: We apologize for the inconvenience, however, there is no English version for the list and we have marked the link [illegible]
“in Chinese”.

Line 75: mention what kind of animal Angiostrongylcantonensis is, e.g. “In addition, A. fulica is also the intermediate host [illegible]
THE PARASITIC NEMATODE Angiostrongylcantonensis”
Reply: We have changed it in the revised ms.

Line 83: “…considered to be one of the most serious threat and a destructive terrestrial gastropod…”
Reply: We have deleted it in the revised ms.

Line 87: “molecular mechanismS UNDERLYINGinvestigations for its broad environmental adaptability”
Reply: We have corrected it in the revised ms.

Line 93: why these tissues specifically?
Reply: These tissues were used for DNA extraction and subsequent high-throughput sequencing, they were selected since
these tissues were not easy to be contaminated by exogenous DNA from other species and the relatively high quantity of
DNA.

Line 123: how does this estimate of heterozygosity (0.47%) compare to other mollusks?
Reply: High rate of heterozygosity (>1%) have been reported in bivalve genomes, and a previous genome study of Poma[illegible]
canaliculata revealed a high heterozygosity of 1%-2%. Thus a heterozygosity of 0.47% may be much lower than other
molluscs.

Line 127: “provided additional supporting data for the statically STATISTICAL analysis”
Reply: We have corrected it in the revised ms.

Line 127: what statistical analysis is being referred to here?
Reply: It means the statistical analysis mentioned in the previous sentence, the correlation between repeat content and
genome size.

Line 153: “pairsthat WITH both ends uniquely mapped”
Reply: We have corrected it in the revised ms.

Line 155: “StartNearRsite”, “ExtremeFragments” etc – the detail is good but some of these parameters could be explaine[illegible]
tell readers what filtering was performed and why
Reply: These are parameters regarding invalid read pairs defined by hiclib and can be filtered with default settings. Actual[illegible]
these parameters have been used extensively (https://doi.org/10.1093/molbev/msw108,
https://doi.org/10.1093/gigascience/giy120).
The details are as follows:
ExtremeFragments: removes fragments with most and/or least # counts (the top 0.005 and bottom 0 were removed)
- StartNearRsite:Removes reads that start within x bp near rsite (5 bp near the rsite)
- LargeSmallFragments: removes very large and small fragments (100bp- 100000bp were retained)

Line 159: “had” -> “has”
Reply: We have corrected it in the revised ms.

Line 169: how many scaffolds? From Table 2 there are ~1000, which is way more that 31 expected number of chromosomes, so I suppose “chromosomal level” is a bit misleading? “near chromosomal level” might be more accurate
Reply: We have corrected it according to your instructions in the revised ms.

Line 186: which BUSCO gene set was used here?
Reply: We used the metazoa_odb9, and it has been added in the revised ms (line 210).

Line 188: so ~15% of detected BUSCO genes were found in multiple copy; is this a reflection of unresolved heterozygosit or genuine gene duplications / paralogs? If the former, what has been done to remove these uncollapsed regions from the assembly? For example, their inclusion might upwardly bias the total assembly size or number of genes
Reply: Thank you very much for your reminding. The possibility can not be ruled out. However, as mentioned above, the sequencing depth shows that almost all regions of the assembly are homozygous, together with the fact that we used metazoa_odb9 as reference, we suspect that the detected multiple copy should be genuine gene duplications / paralogs because of lineage-specific duplication. Moreover, a number of published genomes like Sillago sinica, Protosalanx hyalocranius, etc, detected multiple copy of BUSCO genes, which should be lineage-specific duplications, too.

Line 192: “From the NGS reads alignment, we detected 128,998 homologous SNP loci using the GATK pipeline, demonstrating the high base-level accuracy of 99.33%.” I don’t understand this statement: how does variant calling demonstrate a high base-level accuracy? What exactly does the 99.33% pertain to? How is “base-level” accuracy defined?
Reply: Thank you very for your reminding. The “homologous” should be “homozygous” and we are very sorry for the mistake. Generally, homozygous SNP means assembly error and heterozygous SNP means the assembly maybe right, and has been used in many genome projects like Sillago sinica, Glyptosternon maculatum, etc, although the theory is not too serious. To avoid the confusion, we have deleted the sequence in the revised ms.

Line 197: RepeatModeler
Reply: We have corrected it in the revised ms.
Line 200: “All repetitive elements were masked in the genome for the BEFORE protein-coding gene prediction”
Reply: We have corrected it in the revised ms.

Line 206: “Full-length transcripts WERE obtained using Iso-Seq were mapped to the genome using Genewise” Also this sentence is slightly confusing – is Iso-Seq a tool that has generated ‘transcripts’ from the TBLASTN results in the previous sentence? I did not see any mention of RNA-seq data in the text, but there is some mentioned in Table 2. Please explain i more detail.
Reply: Iso-Seq is a technology and its full name is “isoform-sequencing”, which can generate “full-length” isoforms of the transcripts from the same gene locus, and the details have been added in the revised ms. (lines 106-124 in the revised m

Line 221: Drosophila melanogaster is not a mollusc…
Reply: Drosophila melanogaster is used as an outgroup here and we corrected the mistake in the revised ms (lines 245-2[illegible] in the revised ms).

Line 223: “Only proteins from the longest transcript were usedfroFOR genes with alternative splices ISOFORMS”
Reply: We have corrected it in the revised ms.

Line 234: is this phylogenetic relationship unexpected?
Reply: The relationship (Aplysia_californica,(Achatina_fulica,Biomphalaria_glabrata)) is supported by a paper published in THE NAUTILUS (Title:On the phylogenetic relationships of the genus Mexistrophia and of the family Cerionidae (Gastropod Eupulmonata),
https://repository.si.edu/bitstream/handle/10088/27780/Harasewych%20et%20al.%202015.pdf?sequence=1&isAllowed=
and the relationship between other species is in accord with a paper published in Gigascience (Title: The genome of the golden apple snail Pomacea canaliculata provides insight into stress tolerance and invasive adaptation, https://doi.org/10.1093/gigascience/giy101).

Line 243: “We annotatedPREDICTED 23,726 protein-coding genes in the A. fulica genome and 22,858 of genes were annotated WITH PUTATIVE FUNCTIONS.” Functions based on sequence similarity, BLAST etc are of course putative
Reply: We have corrected it in the revised ms.

Table 2: what do the asterisks** represent?
Reply: It means the ultimate contigs since they were probably changed during the Hic step. We have added the statemen

the revised ms.
Figure 1: "Figure 1. A picture of A. fulicathat INDIVIDUAL used for genome sequencing and assembly"
Reply: We have corrected it in the revised ms.

Figure 2: I struggle to extract anything useful from this figure, but I am not familiar with Hi-C data so maybe it's just me
Reply: The assumption of Hic is that the crosslinking signals are more strong as the loci located in a chromosome are mor closer. Thus ideally the contact matrix should be around the diagonal line, just as is shown in the figure (figure3 in the rev ms).

Figure 3: Again, I'm not convinced this figure is very informative, as it currently is. For example, the majority of (unlabelle points all overlap somewhere near the X-Y intercept, with only three outwith this cluster. Then the size of the points and their colour appear to convey the same information – why twice? I think the point of the figure is to demonstrate the high contiguity of A. fulica genome compared to other mollusc genomes, but does plotting scaffold N50 versus contig N50 reall achieve this? Better would be to plot cumulative assembly span curves, i.e. number of scaffolds on X vs cumulative span o Y
Reply: Thank you very much for your suggestions. We have deleted the figure and listed these parameters such as scaffol N50 and contig N50 in Table 3 for comparison in the revised ms.

Figure 4: It is interesting that exon length is so conserved, but intron lengths are much more variable. Is there any eviden that intron lengths are bimodally distributed?
Reply: Bimodal distribution of the intron lengths was rarely reported. It is not surprise that the intron lengths is more variable than exon since the latter one is much more conservative than the former.

Reviewer #3: I thank the authors for the work presented on the manuscript "A chromosomal-level genome assembly for t giant African snail Achatina fulica". It is a great contribution for future studies of mollusk genomics and for the study of th molecular basis of invasiveness. I just have a few recommendations and comments.

1-) I would like to see the kmer distribution plot presented on the manuscript. It helps future researchers to understand t composition of this mollusk genome, and to plan future projects.
Reply: Thank you very much for your suggestions. In the revised ms, we have added the kmer spectra as Figure 2.

2-) On lines 133-137: Canu and Falcon are both good assemblers generating high quality data. After deciding to move forward with the Falcon assembly, I would like to know why the authors have decided not to run FALCON-Unzip on the assembly? The phasing of haplotypes has been shown to help avoid assembly errors in genomic areas of complex structur variation between haplotypes. Even though the further analysis (mapping quality, etc) show the assembled genome to be good shape, it would be a good standard practice to run Falcon-Unzip before HiC scaffolding.
Reply: Thank you very much for your suggestions and we strongly agree with you. We believe that using Falcon-Unzip wil generate a high-quality genome, especially the heterozygosity of the species is very high (>1% for example). However, w used FALCON here by considering that the heterozygosity of the species is not very high (0.47%).

3-) After Lanchesis, around 1000 contigs were not placed into chromosomes. Have you investigated the composition of su contigs? Can you present also the size distribution of them?
Reply: Thank you very much for your suggestions. We found that the average gene length is much shorter for contigs unanchored to chromosomes than the anchored ones (67.6 bp/kb vs 341.5 bp/kb), whereas the average length of repeat length is just the reverse. Out of the 1467 unanchored contigs, a total of 210 are longer than 10kb, with the longest one i 6,839 kb. And the size distribution of the unanchored contigs short than 10 kb is as follows:

4-) The sequencing of the transcriptome with IsoSeq technology was only briefly mentioned. Could you describe the evaluation of such transcripts in a few lines? For example, was it possible to find full-length transcripts sequenced?
Reply: Thank you very much for your suggestions. In this study, a number of 553,889 Full-length Non-chimeric sequence (FLNC) representing 23,726 gene loci were obtained. However, the 5' end of the mRNA might be degraded before sequencing and we could not detect it as we did for the 3' end since a polyA tail is a sign of completeness for the latter on To evaluate the completeness of the isoforms, we compared them to the predicted mRNAs from genome sequences and found that 70.37% of the multi-exon FLNCs were really full-length sequences. ((lines 106-124 in the revised ms))

5-) Finally, just a last read to review the English would be advised. Two examples of misspelling: The tittle on line 409. Ar 'fro' on line 223.
Reply: Thank you very much for your reminding. We hope that all mistakes have been corrected in the revised version.

Close
