# Supplementary material for: A chromosomal-level genome assembly for the giant African snail Achatina fulica
Source: Gigascience. 2019 Oct 21;8(10):giz124. doi: 10.1093/gigascience/giz124 (PMC6802634; doi:10.1093/gigascience/giz124)
Supplement: giz124_Response_to_Reviewer_Comments_Revision_1 [file giz124_response_to_reviewer_comments_revision_1.pdf]

Author's Response To Reviewer Comments

Close

Reviewer #1: I would like to ask the authors for further explanation regarding RNA quality check. For publication, molecular dating should also be re-analized using standard calibration method based on fossil records.

In the revised manuscript, information about transcriptome was added according to reviewers' suggestions. The authors described that "The RNA quality was checked using ... the 2100 Bioanalyzer (...) with RNA integrity number of 8." (lines 107-109). In general, molluscan total RNA does not show such a high RIN value because 28s rRNA peak is very low. Integrity of molluscan total RNA can be evaluated by checking a sharp peak of 18s rRNA around 1800-2000nt, while RIN is typically 3.0-6.0. Is it possible to show Bioanalyzer summary report?

Reply: Thank you very much. We used the samples with RIN values more than 8 before library construction. We rechecked the Bioanalyzer results carefully and parts of them are shown as follows. Indeed, we found samples with low RIN values, but we eventually selected high-quality samples for the sequencing. We have included the summary report into the Supplementary Figure S1.

In addition, still I seriously concern about molecular dating in Fig.5. Unfortunately, I could not find the figure the authors downloaded from the TIMETREE (www.timetree.org). Thus, in order to retrieve Timetree, I searched term "Protostomia" in the website. According to the data (please see attached file "pairwise_divergence_times.xlsx"), divergent time of Insecta and Gastropoda is 753 MA, which is more or less similar to the value in Fig 5 (811.54 MA). Next, I downloaded the "Timetable", which is a list of literatures ("TimeTree The Timescale of Life.xlsx") referred by the TIMETREE. In the Timetable, however, 8 literatures out of 11 show the divergent time of insects/molluscs is 543-670 MA that is consistent with widely accepted dating (about 600 MA). Since calibration date considerably affects the result, researchers should access not only summary database but also original literatures cited by the database.

Another issue of the molecular dating is that calibration using estimated value may cause overestimation or underestimation. The authors should use fossil record data for calibration. For examples, data referred in the following studies should provide reliable fossil information. These studies also show the divergent time of insects/molluscs is 600-650 MA.

Erwin, D. H. et al. The Cambrian conundrum: early divergence and later ecological success in the early history of animals. Science 334, 1091-1097 (2011).
Simakov, O. et al. Hemichordate genomes and deuterostome origins. Nature 527, 459-465 (2015).
Reply: Thank you very much for your creative suggestions.
In the last version, we estimated the divergence time among these species using the calibrations of Protostomia (642 - 864 MYA) and Mollusca (551 - 628 MYA), which were downloaded from www.timetree.org.
To follow your suggestion, we used two fossil calibrations, the maximum and minimum age of Bivalve/gastropod divergence (543 and 530 Mya), and the maximum age of Mollusk crown group divergence (549 Mya) to re-estimate the divergence time. As a result, we obtained the divergence time of insects/mollusks as ~677 Mya, which was comparable with previous literatures. The results suggested that fossil records may be more rational than database summary for the divergence time estimation.
Thank you very much again for the constructive suggestion.
The corresponding contents have been upgraded in the revised ms.

Reviewer #2: Thanks to the authors for their responses to my comments. They have addressed the majority of my concerns, and I have only a few minor suggestions that might improve the ms before publication.

1. Contamination. It's good that the authors checked their raw data for contamination from non-target organisms prior to assembly. I think they should just briefly mention this fact in the main text of the manuscript, as it will increase confidence from colleagues that their data is of high quality.
Reply: Thank you very much for your suggestions.
The short description have been added in the revised version.(lines 154-156 in the revised ms)

2. Kmer spectra / heterozygosity. I think the authors may have tried to supply a supplementary figure here that was not attached to the revised PDF. In any case, I am content that their final assembly does not overly contain coassembled heterozygous regions. I have only a final minor comment: I would say that the kmer spectrum presented does in fact show some evidence for bimodality - look at the 'shoulder' around ~160X, at approximately 2 times the value of the main coverage peak. This is unlikely to be due to heterozygosity, as those regions would manifest as a peak around half the value of the main coverage peak - but it does suggest that there might be an excess of regions present as 2x duplications in the A. fulica genome. Something the authors may wish to investigate in the future!
Reply: Thank you very much for your suggestions.
The Kmer distribution figure have been changed into Supplementary Figure S2.
Moreover, the 'shoulder' in the figure may denote the high repeat contents of the genome, and we discussed this in the ms.(lines 150-154)

Minor edits:
- Line 86: "chromosome-level genome"
Reply: Thank you very much and we've changed it into "chromosome-level genome".

- Line 86, 88, 91: typos with the name: A. chatina?
Reply: We are really very sorry for the mistake and have changed it into "A. fulica".

- Line 149: via kmer analysis, the genome is 2.12 Gb, but the final assembly size is considerably smaller (~1.85 Gb) - can the authors include a brief explanation for this difference?
Reply: The relatively large difference between the estimated and assembled versions may be resulted from the following 2 possible reasons: the high contents of repeats reside in the genome, and the probably larger size estimated from the Kmer analysis. We have added these reasons in the revised ms (lines168-171).

- Line 168: in my own experience, the major error mode with pacbio data is small (usually 1-bp) deletions at both homopolymers and heterozygous sites. If these deletions hit CDS, they can result in fragmented gene models and low-quality gene annotations. They may also influence SNP calling between samples. Since heterozygosity is low, this seems unlikely to be an issue in this case, and anyway should have been corrected by the Pilon polishing with the Illumina data (which do not suffer from such errors), but I encourage the authors to check the results of Pilon to check that indeed such errors are being corrected here.
Reply: Thank you very much for your suggestions.
We counted the corrected sites from the polish result and found the number of fixed SNPs and ambiguous bases were 718,733 and 3,117, respectively. A total of 4,663,931 small insertions totaling 6,129,524 bases and 634,193 small deletions totaling 1,043,123 bases were also corrected. We found that more small insertions were corrected comparing to the small deletion, which was consistent with the result in previous study (https://dx.doi.org/10.1186%2F1471-2164-13-375).

Close
