# Supplementary material for: A chromosomal-level genome assembly for the giant African snail Achatina fulica
Source: Gigascience. 2019 Oct 21;8(10):giz124. doi: 10.1093/gigascience/giz124 (PMC6802634; doi:10.1093/gigascience/giz124)
Supplement: giz124_Response_to_Reviewer_Comments_Revision_2 [file giz124_response_to_reviewer_comments_revision_2.pdf]

Author's Response To Reviewer Comments

Close

Reviewer #1: Regarding RNA samples, the authors commented that "we eventually selected high-quality samples for the sequencing." and showed Bioanalyzer report of only four samples in the supplementary. In the revised manuscript, however, they described that "RNA was extracted from the pallium, liver, foot, spleen, stomach, gut, heart...". Which four samples or tissues were actually used for RNA extraction and sequencing?
Reply: Thanks a lot for reminding. We have collected multi samples for pallium, liver, foot, spleen, stomach, gut and heart tissues. The qualities of RNA samples were summarized in the following table. We selected the high-quality samples highlighted by red for the PacBio library construction and sequencing in the Supplementary Table S1, covering all tissues that mentioned above. We have accordingly revised the manuscript and supplementary information.

Close
