# Supplementary material for: A chromosomal-level genome assembly for the giant African snail Achatina fulica
Source: Gigascience. 2019 Oct 21;8(10):giz124. doi: 10.1093/gigascience/giz124 (PMC6802634; doi:10.1093/gigascience/giz124)
Supplement: giz124_Reviewer_1_Report_Original_Submission — Takeshi Takeuchi, Ph.D -- 2/7/2019 Reviewed [file giz124_reviewer_1_report_original_submission.pdf]

Reviewer Report

Title: A chromosomal-level genome assembly for the giant African snail Achatina fulica

Version: Original Submission **Date:** 2/7/2019

Reviewer name: Takeshi Takeuchi, Ph.D

Reviewer Comments to Author:

In this study the authors sequenced the genome of the giant African snail Achatina fulica using short and long read technologies as well as a Hi-C scaffolding method, and succeeded to develop chromosomal-level genome assembly. I think the data will contribute to our understanding of the biology of the species.
At the same time I found description of methods is not sufficient in the present manuscript, therefore it should be revised before publication.
In the Introduction the authors mentioned that it is important to study the biology of A. chatina because the species is one of the most threatening invasive species, and is the intermediate host of Angiostrongylus. However, I could not find how the present chromosomal-level genome assembly is useful to address these issues. I would like to request the authors to discuss the point more specifically. This will emphasize the importance of the study.
The information about transcriptome is absent despite the data might be used for gene model prediction (lines 206-207). The authors should describe in detail about the transciptome. For example, from which tissues was RNA extracted? How was the quality of the RNA? How was the stats of RNA-Seq (number of reads, average length, etc.)? In addition, mapping rate of the transcriptome to the genome assembly and gene models will be informative to evaluate the completeness of the assembly and model prediction, respectively.
Lines 178-180
High rate of heterozygosity (>1%) have been reported in bivalve genomes (oysters, scallops, etc.) but not the case in gastropods.
Fig. 3
I would suggest to show the genome assembly comparison data in a table, not in a scatter plot.
In general, scatter plot is used to see the correlation between two variables. This figure is not adequate to compare genome assemblies because 1)correlation between contig and scaffold N50s is not meaningful 2) most of the dots are put at the lower left and indistinguishable.
In addition, references should be cited when the authors used these genome data in the study.
Lines 232-235, Fig. 5
What kinds of fossil record were used for molecular clock calibration? Honestly speaking, I cannot believe the result (Fig.5), showing Spiralia diverged from Ecdysozoa 831 Mya (200 million years before the Ediacaran Period).
Version information of all software used are needed.

Level of Interest

Please indicate how interesting you found the manuscript: Choose an item.

Quality of Written English

Please indicate the quality of language in the manuscript: Choose an item.

Declaration of Competing Interests

Please complete a declaration of competing interests, considering the following questions:

- Have you in the past five years received reimbursements, fees, funding, or salary from an organisation that may in any way gain or lose financially from the publication of this manuscript, either now or in the future?
- Do you hold any stocks or shares in an organisation that may in any way gain or lose financially from the publication of this manuscript, either now or in the future?
- Do you hold or are you currently applying for any patents relating to the content of the manuscript?
- Have you received reimbursements, fees, funding, or salary from an organization that holds or has applied for patents relating to the content of the manuscript?
- Do you have any other financial competing interests?
- Do you have any non-financial competing interests in relation to this paper?

If you can answer no to all of the above, write 'I declare that I have no competing interests' below. If your reply is yes to any, please give details below.

I declare that I have no competing interests.

I agree to the open peer review policy of the journal. I understand that my name will be included on my report to the authors and, if the manuscript is accepted for publication, my named report including any attachments I upload will be posted on the website along with the authors' responses. I agree for my report to be made available under an Open Access Creative Commons CC-BY license (http://creativecommons.org/licenses/by/4.0/). I understand that any comments which I do not wish to be included in my named report can be included as confidential comments to the editors, which will not be published.

Choose an item.

To further support our reviewers, we have joined with Publons, where you can gain additional credit to further highlight your hard work (see: https://publons.com/journal/530/gigascience). On publication of this paper, your review will be automatically added to Publons, you can then choose whether or not to claim your Publons credit. I understand this statement.

Yes Choose an item.
