# Supplementary material for: A chromosomal-level genome assembly for the giant African snail Achatina fulica
Source: Gigascience. 2019 Oct 21;8(10):giz124. doi: 10.1093/gigascience/giz124 (PMC6802634; doi:10.1093/gigascience/giz124)
Supplement: giz124_Reviewer_1_Report_Revision_1 — Takeshi Takeuchi, Ph.D -- 5/31/2019 Reviewed [file giz124_reviewer_1_report_revision_1.pdf]

Reviewer Report

Title: A chromosomal-level genome assembly for the giant African snail Achatina fulica

Version: Revision 1 **Date:** 5/31/2019

Reviewer name: Takeshi Takeuchi, Ph.D

Reviewer Comments to Author:

I would like to ask the authors for further explanation regarding RNA quality check. For publication, molecular dating should also be re-analized using standard calibration method based on fossil records.
In the revised manuscript, information about transcriptome was added according to reviewers' suggestions. The authors described that "The RNA quality was checked using ... the 2100 Bioanalyzer (...) with RNA integrity number of 8." (lines 107-109). In general, molluscan total RNA does not show such a high RIN value because 28s rRNA peak is very low. Integrity of molluscan total RNA can be evaluated by checking a sharp peak of 18s rRNA around 1800-2000nt, while RIN is typically 3.0-6.0. Is it possible to show Bioanalyzer summary report?
In addition, still I seriously concern about molecular dating in Fig.5. Unfortunately, I could not find the figure the authors downloaded from the TIMETREE (www.timetree.org). Thus, in order to retrieve Timetree, I searched term "Protostomia" in the website. According to the data (please see attached file "pairwise_divergence_times.xlsx"), divergent time of Insecta and Gastropoda is 753 MA, which is more or less similar to the value in Fig 5 (811.54 MA). Next, I downloaded the "Timetable", which is a list of literatures ("TimeTree The Timescale of Life.xlsx") referred by the TIMETREE. In the Timetable, however, 8 literatures out of 11 show the divergent time of insects/molluscs is 543-670 MA that is consistent with widely accepted dating (about 600 MA). Since calibration date considerably affects the result, researchers should access not only summary database but also original literatures cited by the database.
Another issue of the molecular dating is that calibration using estimated value may cause overestimation or underestimation. The authors should use fossil record data for calibration. For examples, data referred in the following studies should provide reliable fossil information. These studies also show the divergent time of insects/molluscs is 600-650 MA.
Erwin, D. H. et al. The Cambrian conundrum: early divergence and later ecological success in the early history of animals. Science 334, 1091-1097 (2011).
Simakov, O. et al. Hemichordate genomes and deuterostome origins. Nature 527, 459-465 (2015).

Level of Interest

Please indicate how interesting you found the manuscript: Choose an item.

Quality of Written English

Please indicate the quality of language in the manuscript: Choose an item.

Declaration of Competing Interests

Please complete a declaration of competing interests, considering the following questions:

- Have you in the past five years received reimbursements, fees, funding, or salary from an organisation that may in any way gain or lose financially from the publication of this manuscript, either now or in the future?
- Do you hold any stocks or shares in an organisation that may in any way gain or lose financially from the publication of this manuscript, either now or in the future?
- Do you hold or are you currently applying for any patents relating to the content of the manuscript?
- Have you received reimbursements, fees, funding, or salary from an organization that holds or has applied for patents relating to the content of the manuscript?
- Do you have any other financial competing interests?
- Do you have any non-financial competing interests in relation to this paper?

If you can answer no to all of the above, write 'I declare that I have no competing interests' below. If your reply is yes to any, please give details below.

I declare that I have no competing interests.

I agree to the open peer review policy of the journal. I understand that my name will be included on my report to the authors and, if the manuscript is accepted for publication, my named report including any attachments I upload will be posted on the website along with the authors' responses. I agree for my report to be made available under an Open Access Creative Commons CC-BY license (http://creativecommons.org/licenses/by/4.0/). I understand that any comments which I do not wish to be included in my named report can be included as confidential comments to the editors, which will not be published.

Choose an item.

To further support our reviewers, we have joined with Publons, where you can gain additional credit to further highlight your hard work (see: https://publons.com/journal/530/gigascience). On publication of this paper, your review will be automatically added to Publons, you can then choose whether or not to claim your Publons credit. I understand this statement.

Yes Choose an item.
