# Supplementary material for: A chromosomal-level genome assembly for the giant African snail Achatina fulica
Source: Gigascience. 2019 Oct 21;8(10):giz124. doi: 10.1093/gigascience/giz124 (PMC6802634; doi:10.1093/gigascience/giz124)
Supplement: giz124_Reviewer_2_Report_Revision_1 — Reuben William Nowell, Ph.D. -- 6/3/2019 Reviewed [file giz124_reviewer_2_report_revision_1.pdf]

Reviewer Report

Title: A chromosomal-level genome assembly for the giant African snail Achatina fulica

Version: Revision 1 **Date:** 6/3/2019

Reviewer name: Reuben William Nowell, Ph.D.

Reviewer Comments to Author:

Thanks to the authors for their responses to my comments. They have addressed the majority of my concerns, and I have only a few minor suggestions that might improve the ms before publication.

1. Contamination. It's good that the authors checked their raw data for contamination from non-target organisms prior to assembly. I think they should just briefly mention this fact in the main text of the manuscript, as it will increase confidence from colleagues that their data is of high quality.
2. Kmer spectra / heterozygosity. I think the authors may have tried to supply a supplementary figure here that was not attached to the revised PDF. In any case, I am content that their final assembly does not overly contain coassembled heterozygous regions. I have only a final minor comment: I would say that the kmer spectrum presented does in fact show some evidence for bimodality - look at the 'shoulder' around ~160X, at approximately 2 times the value of the main coverage peak. This is unlikely to be due to heterozygosity, as those regions would manifest as a peak around half the value of the main coverage peak - but it does suggest that there might be an excess of regions present as 2x duplications in the A. fulica genome. Something the authors may wish to investigate in the future!

Minor edits:

- Line 86: "chromosome-level genome"
- Line 86, 88, 91: typos with the name: A. chatina?
- Line 149: via kmer analysis, the genome is 2.12 Gb, but the final assembly size is considerably smaller (~1.85 Gb) - can the authors include a brief explanation for this difference?
- Line 168: in my own experience, the major error mode with pacbio data is small (usually 1-bp) deletions at both homopolymers and heterozygous sites. If these deletions hit CDS, they can result in fragmented gene models and low-quality gene annotations. They may also influence SNP calling between samples. Since heterozygosity is low, this seems unlikely to be an issue in this case, and anyway should have been corrected by the Pilon polishing with the Illumina data (which do not suffer from such errors), but I encourage the authors to check the results of Pilon to check that indeed such errors are being corrected here.

Level of Interest

Please indicate how interesting you found the manuscript: Choose an item.

Quality of Written English

Please indicate the quality of language in the manuscript: Choose an item.

Declaration of Competing Interests

Please complete a declaration of competing interests, considering the following questions:

- Have you in the past five years received reimbursements, fees, funding, or salary from an organisation that may in any way gain or lose financially from the publication of this manuscript, either now or in the future?
- Do you hold any stocks or shares in an organisation that may in any way gain or lose financially from the publication of this manuscript, either now or in the future?
- Do you hold or are you currently applying for any patents relating to the content of the manuscript?
- Have you received reimbursements, fees, funding, or salary from an organization that holds or has applied for patents relating to the content of the manuscript?
- Do you have any other financial competing interests?
- Do you have any non-financial competing interests in relation to this paper?

If you can answer no to all of the above, write 'I declare that I have no competing interests' below. If your reply is yes to any, please give details below.

I declare I have no competing interests

I agree to the open peer review policy of the journal. I understand that my name will be included on my report to the authors and, if the manuscript is accepted for publication, my named report including any attachments I upload will be posted on the website along with the authors' responses. I agree for my report to be made available under an Open Access Creative Commons CC-BY license (http://creativecommons.org/licenses/by/4.0/). I understand that any comments which I do not wish to be included in my named report can be included as confidential comments to the editors, which will not be published.

Choose an item.

To further support our reviewers, we have joined with Publons, where you can gain additional credit to further highlight your hard work (see: https://publons.com/journal/530/gigascience). On publication of this paper, your review will be automatically added to Publons, you can then choose whether or not to claim your Publons credit. I understand this statement.

Yes Choose an item.
