# Supplementary material for: A chromosomal-level genome assembly for the giant African snail Achatina fulica
Source: Gigascience. 2019 Oct 21;8(10):giz124. doi: 10.1093/gigascience/giz124 (PMC6802634; doi:10.1093/gigascience/giz124)
Supplement: giz124_Reviewer_3_Report_Original_Submission — Marcela Uliano da Silva, Ph.D -- 2/25/2019 Reviewed [file giz124_reviewer_3_report_original_submission.pdf]

Reviewer Report

Title: A chromosomal-level genome assembly for the giant African snail Achatina fulica

Version: Original Submission **Date:** 2/25/2019

Reviewer name: Marcela Uliano da Silva, Ph.D

Reviewer Comments to Author:

I thank the authors for the work presented on the manuscript "A chromosomal-level genome assembly for the giant African snail Achatina fulica". It is a great contribution for future studies of mollusk genomics and for the study of the molecular basis of invasiveness. I just have a few recommendations and comments.

1-) I would like to see the kmer distribution plot presented on the manuscript. It helps future researchers to understand the composition of this mollusk genome, and to plan future projects.

2-) On lines 133-137: Canu and Falcon are both good assemblers generating high quality data. After deciding to move forward with the Falcon assembly, I would like to know why the authors have decided not to run FALCON-Unzip on the assembly? The phasing of haplotypes has been shown to help avoid assembly errors in genomic areas of complex structural variation between haplotypes. Even though the further analysis (mapping quality, etc) show the assembled genome to be in good shape, it would be a good standard practice to run Falcon-Unzip before HiC scaffolding.

3-) After Lanchesis, around 1000 contigs were not placed into chromosomes. Have you investigated the composition of such contigs? Can you present also the size distribution of them?

4-) The sequencing of the transcriptome with IsoSeq technology was only briefly mentioned. Could you describe the evaluation of such transcripts in a few lines? For example, was it possible to find full-length transcripts sequenced?

5-) Finally, just a last read to review the English would be advised. Two examples of misspelling: The tittle on line 409. And 'fro' on line 223.

Level of Interest

Please indicate how interesting you found the manuscript: Choose an item.

Quality of Written English

Please indicate the quality of language in the manuscript: Choose an item.

Declaration of Competing Interests

Please complete a declaration of competing interests, considering the following questions:

- Have you in the past five years received reimbursements, fees, funding, or salary from an organisation that may in any way gain or lose financially from the publication of this manuscript, either now or in the future?
- Do you hold any stocks or shares in an organisation that may in any way gain or lose financially from the publication of this manuscript, either now or in the future?
- Do you hold or are you currently applying for any patents relating to the content of the manuscript?
- Have you received reimbursements, fees, funding, or salary from an organization that holds or has applied for patents relating to the content of the manuscript?
- Do you have any other financial competing interests?
- Do you have any non-financial competing interests in relation to this paper?

If you can answer no to all of the above, write 'I declare that I have no competing interests' below. If your reply is yes to any, please give details below.

I declare that I have no competing interests

I agree to the open peer review policy of the journal. I understand that my name will be included on my report to the authors and, if the manuscript is accepted for publication, my named report including any attachments I upload will be posted on the website along with the authors' responses. I agree for my report to be made available under an Open Access Creative Commons CC-BY license (http://creativecommons.org/licenses/by/4.0/). I understand that any comments which I do not wish to be included in my named report can be included as confidential comments to the editors, which will not be published.

Choose an item.

To further support our reviewers, we have joined with Publons, where you can gain additional credit to further highlight your hard work (see: https://publons.com/journal/530/gigascience). On publication of this paper, your review will be automatically added to Publons, you can then choose whether or not to claim your Publons credit. I understand this statement.

Yes Choose an item.
